# eHealth Interventions for Dutch Cancer Care: Systematic Review Using the Triple Aim Lens

**DOI:** 10.2196/37093

**Published:** 2022-06-14

**Authors:** Liza van Deursen, Anke Versluis, Rosalie van der Vaart, Lucille Standaar, Jeroen Struijs, Niels Chavannes, Jiska J Aardoom

**Affiliations:** 1 Department of Quality of Care and Health Economics Center for Nutrition, Prevention and Health Services National Institute for Public Health and the Environment Bilthoven Netherlands; 2 National eHealth Living Lab Leiden University Medical Center Leiden Netherlands; 3 Department of Public Health and Primary Care Leiden University Medical Center Leiden Netherlands; 4 Department of Quality and Organization of Care Netherlands Institute for Health Services Research Utrecht Netherlands; 5 Health Campus The Hague Department of Public Health and Primary Care Leiden University Medical Center The Hague Netherlands

**Keywords:** cancer, eHealth, digital care, Triple Aim, population health, quality of care, costs, systematic review, psychosocial, intervention, mobile phone

## Abstract

**Background:**

Globally, the burden of cancer on population health is growing. Recent trends such as increasing survival rates have resulted in a need to adapt cancer care to ensure a good care experience and manageable expenditures. eHealth is a promising way to increase the quality of cancer care and support patients and survivors.

**Objective:**

The aim of this systematic review was 2-fold. First, we aimed to provide an overview of eHealth interventions and their characteristics for Dutch patients with and survivors of cancer. Second, we aimed to provide an overview of the empirical evidence regarding the impact of eHealth interventions in cancer care on population health, quality of care, and per capita costs (the Triple Aim domains).

**Methods:**

The electronic databases Web of Science, PubMed, Cochrane, and Ovid PsycINFO were searched using 3 key search themes: eHealth interventions, cancer care, and the Netherlands. The identified interventions were classified according to predetermined criteria describing the intervention characteristics (eg, type, function, and target population). Their impact was subsequently examined using the Triple Aim framework.

**Results:**

A total of 38 interventions were identified. Most of these were web portals or web applications functioning to inform and self-manage, and target psychosocial factors or problems. Few interventions have been tailored to age, disease severity, or gender. The results of this study indicate that eHealth interventions could positively affect sleep quality, fatigue, and physical activity of patients with and survivors of cancer. Inconclusive results were found regarding daily functioning and quality of life, psychological complaints, and psychological adjustment to the disease.

**Conclusions:**

eHealth can improve outcomes in the Triple Aim domains, particularly in the population health and quality of care domains. Cancer-related pain and common symptoms of active treatment were not targeted in the included interventions and should receive more attention. Further research is needed to fully understand the impact of eHealth interventions in cancer care on participation, accessibility, and costs. The latter can be examined in economic evaluations by comparing eHealth interventions with care as usual.

## Introduction

### Background

Globally, population health is greatly affected by cancer. An estimated 19.3 million new cancer cases and almost 10 million cancer deaths occurred in 2020 [1]. The related health care expenditure amounted to €103 (US $110) billion in Europe in 2018, corresponding to 6.2% of the total health expenditures [2]. The global cancer incidence is estimated to double by 2035 [3]. Owing to better screening and treatment options, survival rates have increased. Hence, cancer is increasingly becoming a chronic disease. Therefore, it is essential to develop and implement interventions to promote the long-term health and well-being of patients and survivors and to support daily disease coping [4].

Increasing attention is being paid to the use of eHealth to improve cancer care and support patients with cancer and survivors in coping with their illness. The World Health Organization defines eHealth as “the use of information and communication technology in support of health and health-related fields” [5]. There are several definitions of cancer survivors. Here, we use the definition of the National Cancer Institute: “persons with cancer post-treatment until the end of life” [6]. Currently, various eHealth interventions are available for patients with cancer and survivors. These interventions show considerable variations in function, target population, and type of eHealth technology. For instance, interventions can provide patients with and survivors of cancer with information about the disease and its treatment [7,8], support decision-making and self-management [9,10], alleviate physical and emotional problems [11,12], or provide peer social support [13,14]. Furthermore, interventions target different groups of patients with or survivors of cancer using various technologies and can be used as unguided self-help or with the support of health care professionals. Several studies have evaluated specific eHealth interventions in cancer care [15-20]. These studies considered a variety of outcomes, such as psychological complaints [15,16], symptom distress [17,19], and insomnia severity [18], and examined the effect of intervention characteristics, such as the amount of support, on intervention efficacy [21].

Currently, a general overview of eHealth interventions in cancer care and their characteristics is lacking. Such an overview would provide insights into the broad range of eHealth interventions available in cancer care, making it easier to compare interventions and their efficacy. In addition, no reviews that investigate the empirical evidence of the impact of eHealth interventions in cancer care are available. The absence of such overviews limits our understanding of the added value of eHealth interventions in cancer care. One way of evaluating interventions is through the Triple Aim framework. This model focuses on (1) improving population health, (2) improving the quality of care and patient experience, and (3) reducing the per capita health care costs [22]. Many areas of health reform can be helped forward and strengthened by Triple Aim framework, including the integration of information technologies such as eHealth. Deploying the Triple Aim lens offers an opportunity for a holistic and versatile evaluation.

### Objective

The aim of this systematic review is 2-fold: (1) to provide an overview of available eHealth interventions in cancer care and their characteristics as described in the scientific literature and (2) to provide an overview of the empirical evidence regarding the impact of eHealth interventions in cancer care on population health, quality of care, and per capita costs—the Triple Aim domains [23]. As eHealth interventions are likely to be context-specific or even context-dependent, we will examine eHealth interventions applied in the Dutch context [24]. The Dutch context has been chosen as a case study and serves as an example for other Western countries.

## Methods

### Search Strategy

The following 4 databases were searched electronically from the earliest available date to June 14, 2021, to identify relevant literature: Web of Science, PubMed, Cochrane, and Ovid PsycINFO. Three key search components were used: eHealth interventions, cancer, and the Netherlands. An overview of the search strategies for each database can be found in Multimedia Appendix 1. Other potentially relevant publications were identified by tracking the reference lists of included articles.

### Eligibility Criteria

Studies were eligible if the following criteria were met:

- Population: the eHealth intervention was offered in the Netherlands and targeted adults (>18 years) diagnosed with cancer who were about to start, are currently undergoing, or have finished treatment (ie, cancer survivors) within the Dutch health care system.
- Intervention: the study focused on eHealth interventions according to the definition of eHealth by the World Health Organization [5]: “the use of information and communication technology in support of health and health-related fields.” Both fully web-based and blended eHealth interventions (ie, interventions combining web-based components with face-to-face contact) were included [25]. The eHealth intervention did not consist of business intelligence and big data solutions, such as analyzing structured and unstructured data to gather information to support decision-making [26].
- Comparison: studies were included independently of the presence and type of control group.
- Outcome: there was no focus on specific research outcomes for the first aim—to provide an overview of available eHealth interventions. The goal was to obtain a broad picture of available eHealth interventions. For the second aim—to provide an overview of empirical evidence regarding the impact of eHealth interventions—only studies that measured one or more of the Triple Aim domains were included.
- Setting: using any study designs except for incomplete trials, editorials, letters, and reviews. Nonetheless, the latter method was used to identify additional relevant studies from the reference lists. We excluded these 3 study designs as they were non–peer-reviewed or did not discuss a specific intervention.
- Time: all years were included as long as the study was published in the Dutch or English language.

### Selection Procedure

The PRISMA (Preferred Reporting Items for Systematic Reviews and Meta-Analyses) 2020 Statement was used to ensure the validity and reliability of the selection procedure [27]. The PRISMA 2020 checklist can be found in Multimedia Appendix 2 [28]. One investigator (LvD) searched for eligible studies. Subsequently, the reference software program Endnote (Endnote X7; Thomson Reuters) was used to remove duplicates. Two investigators (LvD and LS) independently screened the titles and abstracts of the articles to identify relevant studies. Next, full texts of the potentially relevant articles were assessed. Discrepancies between investigators were mutually resolved through discussion until a consensus was reached. Web-based software Covidence (Veritas Health Innovation) [29] was used for the screening process.

### Data Selection and Extraction

The following intervention characteristics were extracted at the application level (Multimedia Appendix 3 [7-13,21,30-106]):

- Summary of the intervention: a short description of the intervention type (eg, web-based training modules) and purpose.
- Functional category: the functional category classification of the interventions was based on CEN (Comité Européen de Normalisation)-ISO (International Organization for Standardization) DTS (Draft Technical Specification) 82304-2:2020 [107]—a document providing quality requirements for health applications. The following categories were distinguished: (1) inform; (2) simple monitoring, to allow users to record health parameters to create health diaries; (3) communicate, to allow 2-way communication; (4) preventive behavior change, to change intended user behavior, such as related to smoking or sexual health; (5) self-management, to help persons with specific health issues to manage their health; (6) treat, to provide treatment for specific health issues or to guide treatment decisions; (7) active monitoring, to automatically record information for remote monitoring; and (8) diagnose, to use data to diagnose health issues.
- Type of eHealth: the classification of the type of eHealth of the intervention was based on the categorization of Nictiz [26], a Dutch knowledge center for national applications of information and communications technology in health care [108]: (1) web application or web portal (offered via a web browser, place, and time-independent), (2) mobile app (available on a smartphone), (3) health sensor (to measure vital bodily functions) or health gateway (to collect and transmit data from health sensors to medical professionals) or wearable devices (health sensors carried on the body), (4) electronic health records or personal health records, and (5) video communication tools.
- Intended setting to use the intervention: primary care, secondary care, or community
- Target population: type of cancer, demographics (gender, age, and nationality), and specific characteristics (eg, smokers)
- Support of health care professional: yes or no, with an explanation
- Use of theory in the development of the intervention: yes or no, with an explanation
- Stakeholder involvement in the development of the intervention: yes or no, with an explanation

Information on research methods and outcomes was extracted at the study level for each empirical evaluation study. More specifically, we extracted information on the study design and objective, the number of participants included at baseline, description of the control group (if applicable), data collection period, study measures, and outcomes. Study outcomes were classified using the Triple Aim [23]. The Triple Aim describes an approach to improve health system performance by focusing on the following:

1. Improving the health of populations
2. Improving patient experience (including quality, patient-centeredness, safety, and timeliness of care)
3. Reducing the per capita cost of health care [23]

We used the framework by Struijs et al [109,110], who elaborated on this model by breaking down the 3 aims into more concrete dimensions (Textbox 1).

Furthermore, a quality appraisal was conducted for each empirical evaluation study using the Effective Public Health Practice Project Quality Assessment Tool for Quantitative Studies [111]. This tool has been reported to have construct and content validity [112,113]. Furthermore, the tool can be used to gain insight into the quality of different study designs, making it easier to compare the results of the quality appraisal in this review. This tool assesses 6 components: (1) selection bias, (2) study design, (3) confounders, (4) blinding, (5) data collection methods, and (6) withdrawals and dropouts. Each component can be rated as strong, moderate, or weak based on the guidelines for the tool. Based on the ratings of each component, the tool allocates an overall methodological score for the study: strong, moderate, or weak.

**Overview of levels in Triple Aim based framework by Struijs et al [109,110].**

**Population health:**

- Health outcomes
- Disease burden
- Behavioral and physiological factors
- Participation
- Functioning and quality of life

**Quality of care:**

- Patient safety
- Effectivity
- Responsiveness
- Timeliness
- Support
- Accessibility

**Per capita costs:**

- Costs of care
- Volume
- Organizational costs
- Productivity loss

Finally, an overview of funding sources per article can be found in Multimedia Appendix 4.

Customized data extraction sheets were developed for the intervention characteristics and the study design, quality appraisal, and study outcomes. To ensure consistency in data extraction, one researcher (LvD) independently subtracted the data of each study and a second researcher (LS) subtracted data of a random sample of 15% of these studies. The interrater agreement was 83.5%, which was considered good. Data were narratively synthesized in 2 sections. The first section discusses the intervention characteristics of the identified interventions. The second section discusses the study design, quality appraisal, and empirical study outcomes.

## Results

### Study Selection and Characteristics

**Figure 1:**
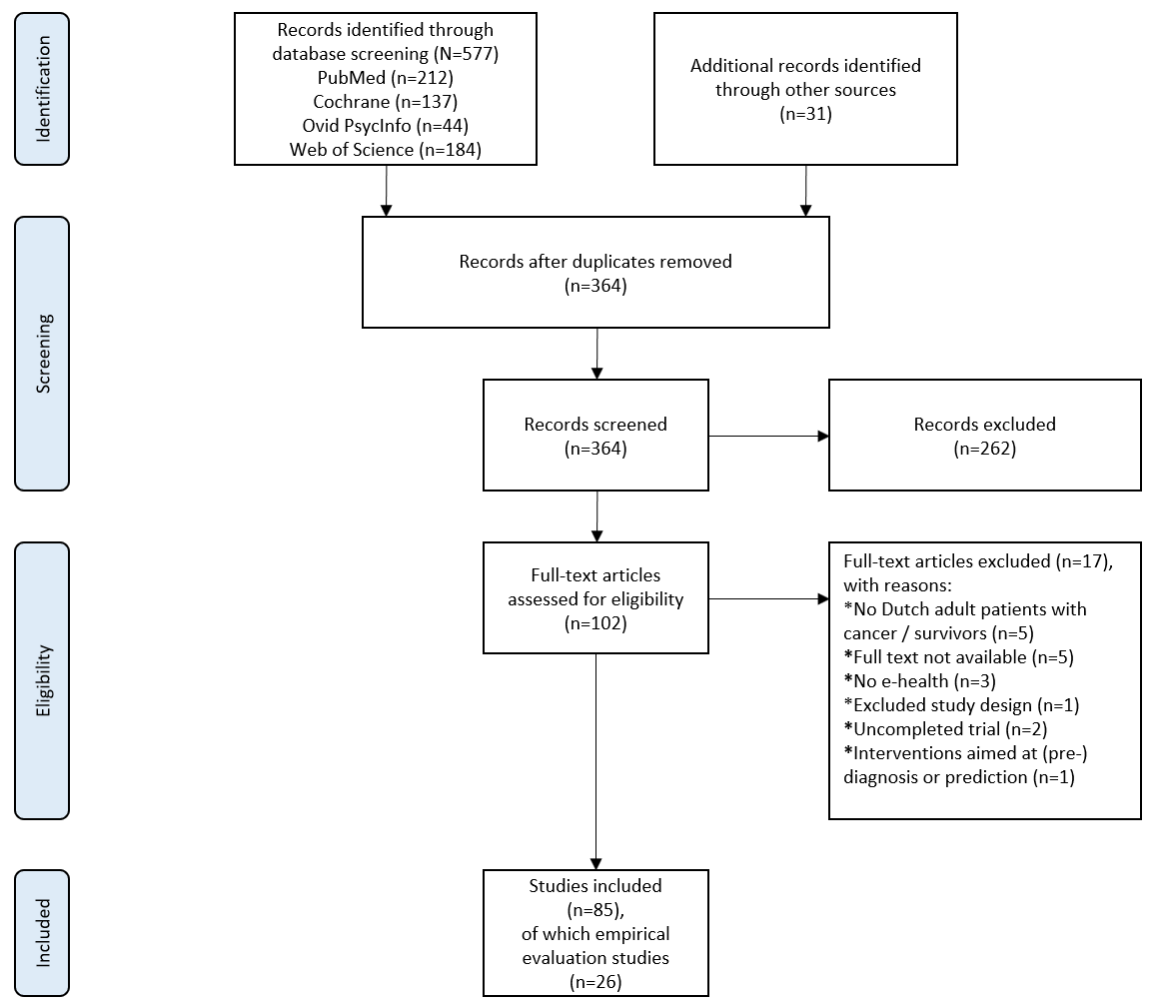
Study selection flow diagram according to PRISMA (Preferred Reporting Items for Systematic Reviews and Meta-Analyses) 2020 [27].

Figure 1 shows the flow diagram of the study selection. We identified 577 articles, and reference tracking yielded an additional 31 peer-reviewed studies. Removal of duplicates resulted in 364 publications. After screening the records and assessing the full-text articles, 85 articles were included in this review. Multimedia Appendix 5 lists excluded studies in the full-text screening stage.

The resulting 85 included articles described 38 unique interventions. An empirical evaluation of eHealth interventions in cancer care was performed in 26 of these 85 articles. These 26 evaluation studies evaluated 18 of the 38 identified eHealth interventions, as in some cases, multiple articles evaluated the same intervention.

The main characteristics of the interventions are described in the subsequent section to provide an overview of available eHealth interventions in cancer care and their characteristics as described in the scientific literature (the first study aim). The described intervention characteristics are purpose, functional category, type of eHealth, setting, target population, support of health care professionals, and the use of theory.

### Intervention Purpose

The included interventions had a broad range of purposes, such as supporting decision-making (eg, decision aids), communicating with health care professionals, monitoring patient-reported outcomes, and participating in online support communities. Almost half of the interventions targeted psychosocial factors (eg, cognitive or sexual functioning and psychological adjustment) or problems (eg, smoking, drinking behavior, depression, and anxiety). Approximately two-thirds of these psychosocial interventions aimed to reduce general psychosocial issues or psychological complaints or foster patients’ self-efficacy or disease coping.

### Functional Category, Type of eHealth Intervention, and Setting

The interventions had various functions, in some cases, more than one. The most common functions were inform (n*=*35), self-manage (n*=*14), treat (n*=*11), and preventive behavior change (n*=*7). Most interventions were web applications or web portals (n*=*34) or mobile apps (n*=*7). Most of the interventions were used in secondary care (n*=*32).

### Target Population

Approximately half (17/38, 45%) of the interventions targeted the general population of patients with cancer or survivors, whereas others targeted a specific type (15/38, 39%) or multiple types (6/38, 16%) of cancer. A total of 14 interventions were aimed at patients or survivors with specific demographics, namely age (eg, young adults or older adult patients; 4/38, 10%), origin (Turkish-Dutch or Moroccan Dutch migrants; 1/38, 3%), or gender (9/38, 24%). The latter interventions were often specifically designed for female patients with or survivors of breast cancer (8/38, 21%). A total of 8 interventions targeted patients or survivors with specific clinical characteristics (eg, smokers and patients with depressive symptoms). Finally, 3 interventions focused on patients with a specific disease severity: stable lower-grade glioma (1/3, 33%) and patients treated with palliative intent (2/3, 67%).

### Support of Health Care Professionals and Use of Theory

Support from a health care professional was possible in 55% (21/38) of the interventions. Support comprised, among others, web-based support from a coach [30,31], weekly feedback from a health care provider [32-34], and teleconsultation with a health care provider [35,36]. Approximately 60% (23/38) of the interventions were theory-based, using, for example, principles from cognitive behavioral theory and the theory of planned behavior.

More details on the intervention characteristics can be found in Multimedia Appendix 3.

Characteristics of the empirical studies and the study results are described in the subsequent sections to provide an overview of the empirical evidence regarding the impact of eHealth interventions in cancer care on population health, quality of care, and per capita costs, the Triple Aim domains (the second study aim).

### Description of Empirical Studies

#### General Characteristics

Table 1 shows the characteristics of the 26 available studies that evaluated 18 different interventions for Dutch patients with or survivors of cancer. Approximately 88% (23/26) of the studies were randomized controlled trials, 8% (2/26) were prospective controlled trials, and 4% (1/26) were a before-and-after design. The control condition involved either usual care (9/26, 35%), being placed on a waiting list to participate after the research period ended (2/26, 8%), a combination of usual care and being placed on a waiting list (9/26, 35%), or receiving another intervention (5/26, 19%). In one study, no control group was used (1/26, 4%). Most studies used 1 (4/26, 15%), 2 (7/26, 27%), or 3 (12/26, 46%) follow-up measurements. One study had 4 follow-up measurements (1/26, 4%) and one did not have follow up measurements (1/26, 4%). The measurement period ranged from 1 week to 1 year after baseline measurement. The average number of patients who participated in the study was 250 (SD 181; range 34-625).

#### Quality Appraisal

A moderate global rating for the quality of evidence was assigned to 16 studies. Six studies were assigned a weak global rating and 4 received a strong global rating. Selection bias was likely present in most studies (18/26, 69%). Most studies were considered to have a low risk of bias concerning the study design, confounders, and data collection. Moderate risk was identified for the majority of studies on the blinding component. Scores for the component withdrawals and dropouts varied considerably. Details can be found in Multimedia Appendix 6 [21,30,32,36-58].

**Table 1:** Characteristics of the empirical evaluation studies.

Intervention			Study design		Participants	Study aim		Description of the control group (CG) usual care (UC)		Data collection period
**Cancer aftercare guide (Kanker Nazorg Wijzer [KNW])**										
	Study 1 [37]	RCT^a^		Total (N=462), IC^b^ (n*=*231), CG (n*=*231)		Present short-term effects of the Cancer Aftercare Guide (KNW) on QoL^c^, anxiety, depression and fatigue	Usual care and a waiting list		BM^d^, follow-up at 3 months, 6 months, and 1 year	
	Study 2 [38]	RCT		Total (N=462), IC (n*=*231), CG (n*=*231)		Explore the influence of gender, age, educational level, and treatment type on intervention effectiveness	Usual care and a waiting list		BM, follow-up at 3 months, 6 months, and 1 year	
	Study 3 [39]	RCT		Total (N=462), IC (n*=*231), CG (n*=*231)		Assess the short-term effects of the KNW on lifestyle outcomes	Usual care and a waiting list		BM, follow-up at 3 months, 6 months, and 1 year	
	Study 4 [40]	RCT		Total (N=462), IC (n*=*231), CG (n*=*231)		Examine the long-term effects of the KNW on moderate physical activity and vegetable consumption	Usual care and a waiting list		BM, follow-up at 3 months, 6 months, and 1 year	
**OncoCompass (OncoKompas)**										
	Study 1 [41]	RCT		Total (N=625), IC (n*=*320), CG (n*=*305)		Evaluate the efficacy of Oncokompas OncoKompas to improve knowledge, skills, and confidence for self-management among survivors of different cancer types	Usual care and a waiting list		BM, follow-up post intervention, and at 3 months and 6 months	
	Study 2 [42]	RCT and economic evaluation		Total (N=625), IC (n*=*320), CG (n*=*305)		Evaluate the cost-utility of Oncokompas compared with usual care among cancer survivors	Usual care and a waiting list		BM, post intervention, and 3 months and 6 months follow-up	
**Transmural Oncological Support (TOS)**										
	Study 1 [43]	PCT^e^		Total (N=36)		Determine the use, appreciation, and effectiveness of an eHealth information support system in head and neck cancer care	N/A^f^		BM, follow-up at 6 weeks	
	Study 2 [44]	PCT		Total (N=184), IC (n*=*145), CG (n*=*39)		Investigate whether telemedicine could be beneficial to the quality of life of patients with cancer	Usual care		BM, follow-up at 6 weeks and 3 months	
Everything under control (Alles onder controle) [31]			RCT		Total (N=115), glioma intervention group (n*=*45), glioma waiting list control group (GWL; n*=*44), non–central nervous system (CNS) cancer control group (n*=*26)	Evaluate the effects of the intervention on depressive symptoms in adult patients with glioma		GWL patients: a waiting list. Non-CNS cancer control group patients: regular intervention		BM, follow-up at 6 and 12 weeks, 6 months, and 12 months
Prostate cancer decision aid (Prostaatkanker keuzehulp) [45]			RCT		Total (N=336), IC (n*=*235), CG (n*=*101)	Compare patients’ evaluation of treatment decision-making process in localized prostate cancer between counseling including an online decision aid (DA) and standard counseling		Usual care		BM, follow-up 1 week after the indicated date of the next consultation
Less tired (Minder Moe) [32]			RCT		Total (N=167), IC 1 (ambulant activity feedback [AAF]; n*=*62), IC 2 (Minder Moe; n*=*55), CG (psychoeducation; n*=*50)	Report on the clinical effectiveness of AAF and eMBCT in reducing fatigue severity and improving mental health in severely fatigued cancer survivors, compared with psychoeducation		Other intervention: psycho-educational mails		BM, follow-up at 2 weeks, 3 months, 6 months, and 12 months
Less tired for anxiety and depression complaints [46]			RCT		Total (N=245), IC 1 (mindfulness based cognitive therapy [MBCT]; n*=*77), IC 2 (eMBCT; n*=*90), CG (treatment as usual [TAU]); n*=*78)	Compare MBCT and eMBCT with treatment as usual for psychological distress in patients with cancer		Usual care		BM, posttreatment, 3 months and 9 months posttreatment
BREATH [47]			RCT		Total (N=150), IC (n*=*70), CG (n*=*80)	Study whether care as usual plus BREATH^g^ can effectively target negative and positive adjustment		Usual care		BM, follow-up at 4, 6, and 10 months
Less fear after cancer (Minder angst bij kanker) [48]			RCT		Total (N=262), IC (n*=*130), CG (n*=*132)	Evaluate the cost-effectiveness of a web-based CBT^h^-based self-help training in reducing fear of cancer recurrence (FCR) in women with curatively treated BC		Usual care		BM, follow-up at 3 months and 9 months
OncoActive [49]			RCT		Total (N=478), IC (n*=*249), CG (n*=*229)	Gain insight into the efficacy of the intervention to increase PA		Usual care and a waiting list		BM, follow-up at 3 and 6 months
PatientTIME [50]			RCT		Total (N=97), IC (n*=*63), CG (n*=*34)	Evaluate if and in what way patients benefit from PatientTIME and if it enhances their confidence in clinical communication		A waiting list		BM, follow-up at T1 (exact timing unclear) and 3 months after participation
ENCOURAGE [51]			RCT		Total (N=138), IC (n*=*70), CG (n*=*69)	Examine the effectiveness of the intervention to empower BC patients to take control over prevailing problems		Usual care		BM, follow-up at 6 and 12 weeks
**Cancer, intimacy, and sexuality (kanker, intimiteit en seksualiteit)**										
	Study 1 [52]	RCT		Total (N=169); IC (n*=*84), CG (n*=*85)		Evaluate the effect of the intervention on sexual functioning and relationship intimacy in BC survivors with sexual dysfunction	Other intervention: receive an information booklet on sexuality issues after BC treatment		BM, follow-up at 10 weeks after the start of therapy and post therapy, at 3 and 9 months	
	Study 2 [53]	RCT		Total (N=169). Only the IC group is taken into account in this study: n*=*84		Evaluate the long-term efficacy of the intervention for sexual dysfunctions in BC survivors	Other intervention: receive an information booklet on sexuality issues after BC treatment		BM, follow-up at 10 weeks after the start of therapy and post therapy, at 3 and 9 months	
Home monitoring tool for adequate pain treatment [54]			Before-and-after design		Total (N=108), IC (n*=*54), CG (n*=*54)	Assess whether home telemonitoring increased registration of pain in medical records of patients visiting a Dutch teaching hospital		Usual care		The authors analyzed medical records from the first 3 visits (a total of 162 visits)
**EvaOnline**										
	Study 1 [21]	RCT		Total (N=254), IC 1 (n*=*85), IC 2 (n*=*85), CG (n*=*84)		Evaluate the efficacy of an iCBT program in women with BC treatment-induced menopausal symptoms	Usual care and a waiting list		BM, follow-up at 10 weeks and 24 weeks	
	Study 2 [55]	RCT and economic evaluation		Total (N=254), IC 1 (n*=*85), IC 2 (n*=*85), CG (n*=*84)		Evaluate the cost-utility, cost-effectiveness, and budget impact of both iCBT formats compared with a waiting list control group	Usual care and a waiting list		BM, follow-up at 10 weeks and 24 weeks	
**Home-based exercise intervention**										
	Study 1 [56]	RCT		Total (N=34), IC (n*=*23), CG (n*=*11)		Present a detailed evaluation of the intervention regarding accrual, attrition, adherence, safety and patient satisfaction	Other intervention: 2 brochures with lifestyle advise		BM, follow-up at 6 months	
	Study 2 [57]	RCT		Total (N=34), IC (n*=*23), CG (n*=*11)		Explore the possible impact of an exercise intervention on cognitive test performance and patient-reported outcomes in patients with glioma	Other intervention: 2 brochures with lifestyle advice		BM, follow-up at 6 months	
My-GMC [58]			RCT		Total (N=109), IC (n*=*59), CG (n*=*50)	Evaluate the efficacy of the intervention		Usual care		BM, follow-up at 1 week, 3 months, and 6 months
Teleconsultation for patients receiving palliative home care [36]			RCT		Total (N=74), IC (n*=*38), CG (n*=*36)	Determine whether weekly teleconsultations improved patient-experienced symptom burden compared with “care as usual”		Usual care		BM, at 4 weeks, 8 weeks, and 12 weeks

^a^RCT: randomized controlled trial.

^b^IC: intervention condition.

^c^QoL: quality of life.

^d^BM: baseline measurement.

^e^PCT: prospective clinical trial.

^f^N/A: not applicable.

^g^BREATH: breast cancer eHealth.

^h^CBT: cognitive behavioral therapy.

#### Study Outcomes

Most studies measured at least one dimension within either the population health or quality of care domain (23 and 24 studies, respectively).

Three studies measured at least one dimension within the per capita costs domain (Table 2 and Multimedia Appendix 7 [21,30,32,36-58]). An overview of the domains and dimensions measured per study can be found in Multimedia Appendix 8 [21,30,32,36-58]. The outcomes are described by dimension in subsequent sections. Unless stated otherwise, significant between-group differences were described by comparing the intervention and control groups.

**Table 2:** Overview of the found effects per empirical evaluation study (randomized controlled trial [RCT] studies, prospective clinical trial [PCT] studies, and before-and-after design studies are study designs).

Intervention				Results^a^
**RCT studies**				
	**Cancer aftercare guide (Kanker Nazorg Wijzer)**			
		Study 1 [37]	e: After 6 months: Emotional functioning sig*^b^. Social functioning *sig*;^*^ MT^c^ *sig.* g: After 6 months: Depression sig**; MT *sig;* ITT sig*. Fatigue sig*; MT *sig;* ITT^d^ sig*. h*:* Participants in the IC who completed the 6-month measurement on average used 2.2 modules. Loss to follow-up in the IC was 16.2%.	
		Study 2 [38]	e: After 12 months: Emotional functioning *n.s. S*ocial functioning *n.s.* g: After 12 months: Depression *n.s.* Fatigue *n.s.* h*:* Overall appreciation of the KNW is 7.48 (10-point scale)*.*	
		Study 3 [39]	c: After 6 months: Moderate PA *sig*;^*^ MT *n.s.* vegetable consumption sig;^*^ MT *n.s.* other PA outcomes *n.s.;* MT *n.s.* other dietary outcomes *n.s.* smoking behavior *n.s.* h: Loss to follow-up after 6 months was low (11.5%) vs mean percentage of dropouts (19.7%) of web-based trials for cancer survivors.	
		Study 4 [40]	c: After 12 months: moderate physical activity sig**. Vegetable consumption *n.s.* h: Loss to follow-up in the IC was 45.5%.	
	**OncoCompass (OncoKompas)**			
		Study 1 [41]	b: The course of symptoms in head and neck cancer survivors, colorectal cancer survivors and high-grade non-Hodgkin lymphoma survivors sig*. The course of symptoms in BC survivors *n.s.* e*:* HRQoL sig*. g: Course of mental adjustment to cancer *n.s.* h*:* Course of supportive care needs *n.s.* Patient-physician interaction over time *n.s.* Self-efficacy *n.s.* Personal control *n.s.* Patient activation *n.s.* In the IC, 78% activated their account and 52% used the intervention as intended.	
		Study 2 [42]	h: The loss to follow up in the IC was 36%. l: OncoCompass is likely to be equally effective on utilities and not more expensive than usual care.	
	Everything under control (Alles onder controle) [31]		e: Physical health after 12 months ITT and protocol analysis n*.s.* g: After 6 weeks: Depression (GI vs GWL group and Total glioma group vs non-CNS cancer group) *n.s.* Fatigue (GI vs GWL group) sig*. After 12 weeks: depression *n.s.* Fatigue *n.s.* Other measures (GI vs GWL group) *n.s.* h: Most patients said they had benefitted from participating (73% glioma; 67% non-CNS), and the program was useful (92% in both groups) and informative (86% glioma; 92% non-CNS). The participation rate was 40%. The adherence of the IC was 85% for the introduction and 77%, 52%, 40%, 37%, and 35% for modules 1 through 5, respectively.	
	Prostate cancer decision aid (Prostaatkanker keuzehulp) [45]		h: Satisfaction with information sig*. Involvement *n.s.* Decisional conflict *n.s.* Knowledge scores *n.s.* Subjective knowledge sig**. Objective knowledge *n.s.*	
	Less tired (Minder Moe) [32]		g: Fatigue severity sig*. Psychic complaints *n.s.* Positive and negative affect *n.s.* h: The proportion of participants who dropped out before completing 6 weeks of the protocol was 18% in the AAF condition, 38% in the eMBCT, and 6% in the psychoeducation condition.	
	Less tired for anxiety and depression complaints [46]		b: Psychiatric diagnosis *n.s.* c: Mindfulness skills sig*. e: Mental HRQoL sig*. Positive mental health sig*. Physical HRQoL *n.s.* g: Psychological distress sig**. Fear of cancer recurrence sig*. Rumination sig*. h*:* 90.9% started MBCT and 92.2% completed ≥4 sessions. 91.1% started eMBCT and 71 completed ≥4 sessions. The dropout rate was higher in eMBCT than in the MBCT.	
	BREATH [47]		g: At T1: Distress sig*. 5 out of 7 negative adjustment variables (general and cancer-specific distress, fatigue, and 2 fear of cancer recurrence outcomes) and 3 out of 10 positive adjustment variables (self-efficacy, remoralization, new ways of living) sig*. Clinically significant improvement sig*. At T2 and T3*:* Distress *n.s.* One negative adjustment variable (Fear of cancer recurrence) sig*. One positive adjustment outcome (Acceptance) sig**. All other outcomes *n.s.* h: At T1: Empowerment *n.s.* The frequency of logins ranged from 0 to 45. Total duration ranged from 0 to 2.324 minutes.	
	Less fear after cancer (Minder angst bij kanker) [48]		g: Fear of cancer recurrence *ns* h: The dropout rate in the IC was 30%*.*	
	OncoActive [49]		c: At 3 months: PA sig;^*^ ITT *sig.* e: At 3 months: Physical functioning sig;^**^ ITT *sig.* HRQoL *n.s.* At 6 months follow-up: physical functioning sig;^*^ ITT *n.s.* HRQoL *n.s.* g: At 3 months follow-up: Fatigue sig*. At 6 months follow-up: Fatigue sig**. Depression sig,^**^ ITT *sig.* Anxiety *n.s.* h: Dropout rates were 4.4% at 3-month follow-up and 7.3% at 6-month follow-up.	
	PatientTIME [50]		h: System usability scale: 73 points (100-point scale), considered “good.” At T1 and T2*:* PEPPI score *n.s.* The participation rate was 90%.	
	ENCOURAGE [51]		e: At T2: QoL *n.s.* g: At T1: Increased acceptance *n.s.* Other primary outcomes *n.s.* At T2: All outcomes *n.s.* h: Usefulness score of the program 3.75 (5-point scale). At T1: Being better-informed sig*. At T2*: n.s.* 61% of the patients logged in more than once.	
	**Cancer, intimacy, and sexuality (Kanker, intimiteit en seksualiteit)**			
		Study 1 [52]	e: At T1: Sexual desire sig**. Sexual pleasure sig**. Discomfort during sex sig**. Orgasmic function *n.s.* Sexual satisfaction *n.s.* Sex frequency *n.s.* Relationship intimacy *n.s.* Marital functioning *n.s.* Health-related quality of life *n.s.* At T2: Overall sexual functioning sig*. Sexual desire sig**. Sexual arousal sig**. Vaginal lubrication sig*. Sexual pleasure. Discomfort during sex sig**. Orgasmic function *n.s.* Sexual satisfaction *n.s.* Sex frequency *n.s.* Relationship intimacy *n.s.* Marital functioning *n.s.* Health-related quality of life *n.s.* g: At T1: Menopausal symptoms sig**. Body image sig**. Psychological distress *n.s.* At T2: Menopausal symptoms *n.s.* Body image sig**. Psychological distress *n.s.* h: The CBT was completed by 61.9% of women.	
		Study 2 [53]	a: *Only time effect was taken into account as T3 and T4 assessments were completed only by the IC*. At T3 and T4: general health *positive* *effect was maintained.* e: At T3 and T4: Sexual functioning, sexual desire, vaginal lubrication, sexual satisfaction, discomfort during sex, sexual distress, marital sexual satisfaction *positive* *effect maintained.* Sex frequency, intellectual intimacy, and sexual pleasure *decreased over time*. Marital satisfaction and other health-related quality of life domains *n.s. time effect.* g*:* At T3 and T4: Menopausal symptoms and body image *positive effect maintained,* quadratic effect *n.s. time effect.* Distress *n.s. time effect.* h*:* The CBT was completed by 61.9% of women.	
	**EvaOnline**			
		Study 1 [21]	e: Sexual functioning *n.s.* HRQoL *n.s.* g: At T1: Both IC groups’ (guided and self-managed) perceived impact of HF and NS sig**. Guided group overall levels of menopausal symptoms sig**. Both IC groups sleep quality sig**. Guided hot flush frequency *sig.* Guided group night sweats frequency sig**. Psychological distress *n.s.* *h:* Minimum compliance rate was 90.6% for the guided and 78.8% for the self-managed IC’s.	
		Study 2 [55]	l: The guided and self-managed iCBT are cost-effective. Self-managed iCBT is the most cost-effective strategy.	
	**Home-based exercise intervention**			
		Study 1 [56]	c: Self-reported physical activity at 6 months sig*. BMI at 6 months *n.s.* Mean absolute VO_2_ peak at 6 months *n.s.* Aerobic fitness at 6 months *sig.* h*:* 16 (84%) patients evaluated the physical exercise program as good or excellent, and 4 as moderately or sufficiently satisfactory. Mean adherence was 79%.	
		Study 2 [57]	e: For attention, 4 measures (attentional inhibition, attention span, auditory selective attention, and working memory) *sig.* Information processing speed *sig*. Sustained selective attention *n.s.* For memory, immediate verbal recall *sig.* Two measures of executive function (auditory working memory and alternating attention) *sig.* One of 2 measures of cognitive functioning *sig.* Mood *sig.* Mental health-related quality of life *sig.* Brain cancer-specific health-related quality of life scales *n.s.* h*:* Loss to follow-up in the IC was 8.7%. g: Two scales of fatigue (physical fatigue and reduced activity) *sig.* Sleep *sig.*	
	My-GMC [58]		c: Medication adherence at T2 *sig.* e: Quality of life at all time points *n.s.* g*:* Distress at all time points *n.s.* Cancer worry at all time points *n.s.* h: Satisfaction with the online app was rated 2.8 (5-point scale). Professional satisfaction with the video GMCs was 2.7 (5-point scale). Empowerment at all time points *n.s.* The participation rate was 35%.	
	Teleconsultation for patients receiving palliative home care [36]		b: Symptom burden *n.s.* g: Anxiety *n.s.* Depression *n.s.* All 3 subscales for continuity of care *n.s.* h: Study outcome measures regarding GP contacts and complex interventions *n.s.* Mean number of unmet needs *n.s.* The attrition rates were 61% in the IC and 53% in the CG. m: Mean number of hospital admissions *n.s.*	
**PCT studies**				
	**Transmural oncological support**			
		Study 1 [43]	h: The average score of all patients for the monitoring function was 8.0 (10-point scale). The average score rated by 7 GPs of the electronic health information support system was 5.6 (10-point scale). The participation rate was 66%. All patients used the system.	
		Study 2 [44]	e: After the intervention: 5 of the 22 QoL subscales (state anxiety, fear related to specific head and neck problems, physical self-efficacy, perceived abilities in swallowing and food intake, and general physical complaints) *sig.* At 3 months: 1 subscale (physical self-efficacy) sig*. Other subscales *n.s.* h*:* The participation rate in the IC was 66%, and 35 out of 39 patients completed all questionnaires.	
**Before-and-after design studies**				
	Home monitoring tool for adequate pain treatment [54]		g: Total number of “pain registrations” in the medical records sig*.	

^a^Triple Aim domains: a=health outcomes, b=disease burden, c=behavioral and physiological factors, d=Participation, e=Functioning and quality of life, f=Patient safety, g=Effectivity, h=Responsiveness, I=Timeliness, j=Support, k=Accessibility, l=Costs of care, m=Volume, n=Organizational costs, o=Productivity loss.

^b^*sig*=significant positive between-group difference in favor of IC, *P* value unknown; sig*=significant positive between-group difference in favor of IC, α≤.05; sig^**^=significant positive between-group difference in favor of IC, α≤.01; *ns*=nonsignificant between-group difference in favor of IC.

^c^*MT*=controlling for multiple testing or comparisons;

^d^*ITT*=intention-to-treat analysis.

#### Population Health

A total of 23 studies measured at least one dimension within the *population health* domain, and 6 studies measured the dimension *behavioral and physiological factors* [39,40,46,49,56,58]. Positive effects were found for aerobic fitness [56] and physical activity [39,49,56]; however, these effects did not always hold after controlling for multiple testing [39] or in follow-up studies [40]. There were also significant effects on mindfulness skills [46] and medication adherence [58]. No effects were found for smoking behavior [39,40], physical fitness level [56], and changes in BMI [56]. A total of 13 studies measured the dimension *functioning and quality of life* [21,31,37,38,41,44,46,49,51-53,57,58]. Six studies focused on daily functioning. The studies showed positive effects for emotional and social functioning [37]; however, these effects were not significant at follow-up [38]. Furthermore, positive effects were found for physical functioning [49]; however, these effects were not significant after multiple testing [49]. One study demonstrated positive effects on cognitive functioning [57]. Mixed effects were found in terms of sexual functioning [21,53]. Most studies measuring health-related quality of life did not find positive effects (4/6, 67%) [21,41,44,49,51,58]. Positive effects were found for mental health-related quality of life [46,57] but not for physical health [31,46]. The dimensions *health outcomes* (*n=*1) [53] and *disease burden* (*n=*3) [36,41,46] were less prevalent, and the dimension *participation* was not studied at all.

#### Quality of Care

A total of 24 studies measured at least one dimension within the domain *quality of care*. Furthermore, 17 studies measured the dimension *effectivity* [21,31,32,36-38,41,46-49,51-54,57,58]. Most of these studies examined the effect of eHealth interventions on psychological complaints (n*=*12; eg, depression, anxiety, and psychological distress). Of these 12 studies, more than half (7/12, 58%) did not find positive effects [21,31,32,36,52,53,58]. Four studies found positive effects [37,46,47,49]; however, no significant results were found in 2 studies that measured the follow-up effects [38,47]. Six studies assessed positive or negative adjustment to cancer (eg, fear of cancer recurrence, mental adjustment, and acceptance), and half of them (3/6, 50%) found positive effects [41,46-48,51,58]. Except for one study, all studies measuring fatigue and sleep quality found positive effects (6/7, 86%) [21,31,32,37,38,49,57]; however, in both studies, where follow-up effects were measured, no significant results were found [31,38]. All studies measuring menopausal symptoms or body image found positive effects [21,52,53]. In total, 7 studies measured outcomes within the dimension *responsiveness* [36,41,45,47,50,51,58]. Mixed effects were found in studies measuring responsiveness in the form of patient-physician interaction (eg, satisfaction with information, patient-physician interaction over time) [36,41,45,51]: 2 found positive effects [45,51] and 2 did not [36,41]. In addition, 80% (4/5) studies measuring patient involvement in the care process (eg, empowerment, patient activation, self-efficacy, shared decision-making, and being better informed) found positive effects [41,45,47,50,58]. The interventions used different scales and outcome measures to measure patients’ and health care providers’ experiences with the intervention. The outcome measures were satisfaction rate, usability, and overall appreciation. Overall, users were fairly positive about their experiences with the intervention and gave satisfactory ratings [31,37,43,50,51,56,58]. Participation in the intervention was also assessed using several outcome measures. The most frequently used measurements were loss to follow-up and participation rate. The loss to follow-up ranged from 8.7% to 45.5% and the participation rate ranged from 35% to 90% [21,31,32,36-53,56-58]. None of the studies measured the dimensions *patient safety*, *timeliness*, *support*, or *accessibility*.

#### Per Capita Costs

Three studies measured a dimension within the domain *per capita costs* [42,54,55]. Two studies [42,55] measured the dimension *costs of care*, and both found through economic evaluation that the intervention was likely to be equally cost-effective compared with care as usual. One study [54] measured the dimension *volume* and did not find significant effects. None of the studies measured the dimensions *organizational costs* or *productivity loss*.

## Discussion

### Principal Findings

This systematic review is the first to provide an overview of eHealth interventions in Dutch cancer care and use the Triple Aim framework to examine the empirical evidence of these interventions on population health, quality of care, and per capita costs (the Triple Aim domains). The review focused on Dutch cancer care; however, the results are also relevant to other Western countries involved in digital care for patients with and survivors of cancer. A total of 38 interventions were identified, and the results showed that most eHealth interventions targeted psychosocial factors or problems. In addition, interventions were aimed at many different target groups, including the general population of patients with and survivors of cancer, patients with a specific type of cancer, or patients who experienced a specific problem, such as cancer-related fatigue or smoking behavior. Few interventions were tailored to age, gender, or disease severity. The most common intervention types studied were web portals or web applications. These function to inform and facilitate self-management. Other types of interventions (eg, electronic health records or video communication tools), functions (eg, communication or diagnosis), and target outcomes (eg, communication with health care professionals or access to electronic health records) were rarely found.

Most outcome measures could be related to the Triple Aim domains *population health* and *quality of care*, whereas the *per capita costs* domain was largely neglected. Within the population health domain, mixed effects were found regarding the impact of eHealth on functioning and quality of life. Most studies measuring behavioral and physiological factors found positive effects. More specifically, there was preliminary evidence for the positive effects of eHealth interventions on physical activity and aerobic fitness. None of the studies considered the dimension *participation*, including outcome measures such as social inclusion. Within the quality of care domain, eHealth interventions seemed effective in increasing sleep quality and decreasing fatigue, in line with a meta-analysis showing that eHealth interventions effectively manage fatigue in highly fatigued cancer survivors [114]. Findings in terms of positive and negative adjustment to cancer and psychological complaints were inconsistent. One of the measures that was not considered was accessibility, which is worthy of mention as there is increasing global awareness that eHealth should be equally accessible to different populations [115]. The per capita cost dimension was largely neglected in the evaluation studies; only 3 studies considered dimensions within this domain.

This study yielded several interesting findings. With 38 interventions in Dutch cancer care, there appears to be a wide range of eHealth interventions for patients with and survivors of cancer. It seems valuable that most interventions targeting psychosocial factors or problems were aimed at general psychosocial issues, psychological complaints, patients’ self-efficacy, and disease coping. Recent research shows that almost all cancer survivors are affected by fatigue [116], 1 in 2 patients with cancer is significantly distressed, and 47% have problems *getting around* [117]. In contrast, few interventions focused on pain from cancer, which is experienced by half of the patients with cancer during active treatment and 65% of the patients with advanced disease [118]. Some common symptoms of active treatment, such as vomiting, nausea, and constipation [119], were not considered. The lack of tailored interventions according to age, gender, or disease severity is noteworthy as subgroups within these categories are likely to have different preferences and needs. For example, older patients may find it more challenging to use eHealth interventions [120]. In addition, patients in different stages of the disease may have different needs as far as information and support are concerned [14].

We found that most interventions consisted of a specific type (web portals or web applications), function (information provision or facilitation of self-management), and target outcome (psychosocial factors or problems). We assume that besides the interventions we identified, more eHealth interventions are being developed and used by patients with or survivors of cancer. These interventions are likely to be designed or evaluated for a broader target population than patients with and survivors of cancer alone. For example, multiple studies have evaluated the general use of electronic health records and patient portals in academic hospitals without targeting a specific patient population [121-124]. Our search strategy included only patients with or survivors of cancer as a critical criterion; therefore, our search results did not include these interventions. As a result, the number of interventions available for patients with and survivors of cancer may be more significant and versatile than the results of this review.

Another interesting finding is that the results of the evaluation of study outcomes are mainly in line with the literature. For example, several meta-analyses have been conducted to examine the effect of eHealth on the quality of life of patients with or survivors of cancer do find a statistically significant effect [114,125], while others do not [126,127]. These mixed findings, which we also found in the review, can be explained by the fact that quality of life is a multidimensional variable influenced by multiple factors [128]. The current inconsistent findings for psychological complaints and adjustment to cancer were also found in a previous meta-review, which found inconsistent results for the effect of eHealth on psychological well-being, depression, and anxiety in patients with cancer [14]. When interpreting the study results, it is important to remember that many eHealth interventions are not implemented in daily practice. In addition, many expected benefits of such interventions are not realized in daily clinical practice [129,130] as they are not being used as intended [131,132]. The latter has several root causes such as lack of trust and digital literacy [133]. The suboptimal use of eHealth interventions in daily practice is a significant problem that future research needs to address.

Finally, it is notable that some domains and dimensions are primarily omitted from the studies, such as per capita costs and participation. The scarcity of per capita cost-related study outcomes is in line with previous research on the effectiveness of eHealth interventions in cancer detection, treatment, and survivorship care [134]. As health care costs are increasing in most countries, organizations are actively trying to develop solutions to curb health care expenditures while maintaining access to and harnessing the quality and safety of health care [135]. Digital health care is often viewed as a solution to increasing health care costs. Evaluating eHealth interventions is relevant for adequate resource allocation decisions and designing services for competing health interventions and limited resources. Participation is also an essential theme for eHealth because eHealth interventions can either foster social inclusion or create new risks of social exclusion (eg, for digitally illiterate patients) [136]. In future studies, it will be essential to consider the needs of patients at risk of social exclusion when developing and evaluating eHealth interventions.

### Limitations

This review had some limitations. First, this review may not have included all available eHealth interventions, as not all available interventions have been scientifically evaluated. Gray literature and ongoing studies in trial registries were not included in this review, nor were experts consulted nor the authors contacted. Second, the Triple Aim framework used in this review provides a comprehensive overview of the domains and dimensions. However, creating an objective distinction between different dimensions was not always possible. For example, an outcome measure such as improved sleep quality could be classified as *effectiveness* or *behavioral or physiological factors*. Hence, categorizing outcome measures into different dimensions was, to some extent, subjective. Third, for each category of study outcomes, we examined only a small number of studies that evaluated the impact of the intervention on the outcome. Publication bias was not investigated in this study. Therefore, we should be cautious about the conclusions drawn regarding the impact of eHealth interventions on certain subdimensions. Finally, the study protocol was not registered.

### Future Research

Future research should examine the dimensions of the Triple Aim that have rarely or not been taken into account in previous research, such as participation and accessibility. Furthermore, studies should examine in further detail what explains the mixed results for studies measuring specific dimensions such as functioning and quality of life. This could be done, for example, in experimental studies examining the effect of particular intervention characteristics on the Triple Aim domains. Further research is needed to increase our understanding of how different intervention characteristics influence intervention outcomes and the underlying causal mechanisms that cause an intervention to be effective. Interventions aimed at coping with pain were rarely found. eHealth interventions such as digital training to develop pain coping skills and pain management apps custom-made for patients with cancer have proven feasible and effective in decreasing pain [137,138]. Future research should explore the potential of such interventions in the Dutch context. Furthermore, this review may be repeated in other countries to compare the intervention characteristics and outcomes of eHealth interventions in cancer care internationally, facilitating learning and sharing best practices. Finally, this review focused on specific eHealth interventions in cancer care. Research on the structural embedding of eHealth interventions in care processes is essential for optimally deploying these interventions. Therefore, future research can examine local care pathways to identify new possibilities for eHealth to address challenges and needs across existing care pathways. Potentially, these insights may lead to new care pathways to optimize cancer care quality. *Conclusions*

Most of the 38 interventions in this review included eHealth interventions for patients with or survivors of cancer in the Dutch health care system consisting of a specific type (web portals or web applications), function (information provision and facilitation of self-management), and target outcome (psychosocial factors or problems). Almost none of the interventions were tailored to the needs of patients with or survivors of cancer based on age group, gender, or disease severity. The Triple Aim domains *population health* and *quality of care* have been studied thoroughly, whereas the domain *per capita costs* is understudied. Most of the included evaluation studies were assigned a moderate quality appraisal score, and selection bias was likely present in most studies. Our results indicate that eHealth could benefit patients and survivors by improving sleep quality, reducing fatigue, and increasing physical activity. Further research is needed to fully understand the effect of eHealth on aspects such as participation (in the form of social inclusion), accessibility, and the effect on quality of life, patient behavior, physiological health, psychological well-being, and per capita costs. Finally, more economic evaluation of eHealth interventions is required. Overall, continuing a holistic evaluation of eHealth interventions in cancer care will be critical to improve population health, enhance the quality of care, and decrease per capita costs.

## Appendix

Multimedia Appendix 1 Overview of search strategies per database.

Multimedia Appendix 2 PRISMA (Preferred Reporting Items for Systematic Reviews and Meta-Analyses) 2020 checklist.

Multimedia Appendix 3 Characteristics of eHealth interventions for cancer care in the Netherlands.

Multimedia Appendix 4 Overview of funding sources per included study.

Multimedia Appendix 5 List of excluded studies in the full-text screening stage.

Multimedia Appendix 6 Quality appraisal of the empirical evaluation studies.

Multimedia Appendix 7 Overview of outcome measurements and found effects per empirical evaluation study.

Multimedia Appendix 8 Overview of measured study outcomes per empirical study.

## Abbreviations

PRISMA: Preferred Reporting Items for Systematic Reviews and Meta-Analyses

## References

[1] Sung H, Ferlay J, Siegel RL, Laversanne M, Soerjomataram I, Jemal A, Bray F (2021). Global Cancer Statistics 2020: GLOBOCAN estimates of incidence and mortality worldwide for 36 cancers in 185 countries. CA Cancer J Clin.

[2] Hofmarcher T, Lindgren P, Wilking N, Jönsson B (2020). The cost of cancer in Europe 2018. Eur J Cancer.

[3] Pilleron S, Sarfati D, Janssen-Heijnen M, Vignat J, Ferlay J, Bray F, Soerjomataram I (2019). Global cancer incidence in older adults, 2012 and 2035: a population-based study. Int J Cancer.

[4] Grassi L, Spiegel D, Riba M (2017). Advancing psychosocial care in cancer patients. F1000Res.

[5] (2019). WHO guideline: recommendations on digital interventions for health system strengthening: web supplement 2: summary of findings and GRADE tables. World Health Organization.

[6] Mayer DK, Nasso SF, Earp JA (2017). Defining cancer survivors, their needs, and perspectives on survivorship health care in the USA. Lancet Oncol.

[7] Schook RM, Linssen C, Festen J, Schramel FM, Lammers E, Zaanen P, Postmus PE (2013). Website visitors asking questions online to lung cancer specialists: what do they want to know?. Interact J Med Res.

[8] Linssen C, Schook RM, The AM, Lammers E, Festen J, Postmus PE (2007). A web site on lung cancer: who are the users and what are they looking for?. J Thorac Oncol.

[9] Keikes L, de Vos-Geelen J, de Groot JW, Punt CJ, Simkens LH, Trajkovic-Vidakovic M, Portielje JE, Vos AH, Beerepoot LV, Hunting CB, Koopman M, van Oijen MG (2019). Implementation, participation and satisfaction rates of a Web-based decision support tool for patients with metastatic colorectal cancer. Patient Educ Couns.

[10] van den Berg M, van der Meij E, Bos AM, Boshuizen MC, Determann D, van Eekeren RR, Lok CA, Schaake EE, Witteveen PO, Wondergem MJ, Braat DD, Beerendonk CC, Hermens RP (2021). Development and testing of a tailored online fertility preservation decision aid for female cancer patients. Cancer Med.

[11] Drijver AJ, Reijneveld JC, Wesselman LM, Klein M (2021). A Web-based lifestyle intervention aimed at improving cognition in patients with cancer returning to work in an outpatient setting: protocol for a randomized controlled trial. JMIR Res Protoc.

[12] Hummel SB, van Lankveld JJ, Oldenburg HS, Hahn DE, Broomans E, Aaronson NK (2015). Internet-based cognitive behavioral therapy for sexual dysfunctions in women treated for breast cancer: design of a multicenter, randomized controlled trial. BMC Cancer.

[13] Kaal SE, Husson O, van Dartel F, Hermans K, Jansen R, Manten-Horst E, Servaes P, van de Belt TH, Engelen LJ, Prins JB, Verberne S, van der Graaf WT (2018). Online support community for adolescents and young adults (AYAs) with cancer: user statistics, evaluation, and content analysis. Patient Prefer Adherence.

[14] Slev VN, Mistiaen P, Pasman HR, Verdonck-de Leeuw IM, van Uden-Kraan CF, Francke AL (2016). Effects of eHealth for patients and informal caregivers confronted with cancer: a meta-review. Int J Med Inform.

[15] Børøsund E, Cvancarova M, Moore SM, Ekstedt M, Ruland CM (2014). Comparing effects in regular practice of e-communication and Web-based self-management support among breast cancer patients: preliminary results from a randomized controlled trial. J Med Internet Res.

[16] David N, Schlenker P, Prudlo U, Larbig W (2013). Internet-based program for coping with cancer: a randomized controlled trial with hematologic cancer patients. Psychooncology.

[17] Kearney N, McCann L, Norrie J, Taylor L, Gray P, McGee-Lennon M, Sage M, Miller M, Maguire R (2009). Evaluation of a mobile phone-based, advanced symptom management system (ASyMS) in the management of chemotherapy-related toxicity. Support Care Cancer.

[18] Ritterband LM, Bailey ET, Thorndike FP, Lord HR, Farrell-Carnahan L, Baum LD (2012). Initial evaluation of an Internet intervention to improve the sleep of cancer survivors with insomnia. Psychooncology.

[19] Donovan HS, Ward SE, Sereika SM, Knapp JE, Sherwood PR, Bender CM, Edwards RP, Fields M, Ingel R (2014). Web-based symptom management for women with recurrent ovarian cancer: a pilot randomized controlled trial of the WRITE Symptoms intervention. J Pain Symptom Manage.

[20] Denis F, Basch E, Septans AL, Bennouna J, Urban T, Dueck AC, Letellier C (2019). Two-year survival comparing Web-based symptom monitoring vs routine surveillance following treatment for lung cancer. JAMA.

[21] Atema V, van Leeuwen M, Kieffer JM, Oldenburg HS, van Beurden M, Gerritsma MA, Kuenen MA, Plaisier PW, Lopes Cardozo AM, van Riet YE, Heuff G, Rijna H, van der Meij S, Noorda EM, Timmers GJ, Vrouenraets BC, Bollen M, van der Veen H, Bijker N, Hunter MS, Aaronson NK (2019). Efficacy of Internet-based cognitive behavioral therapy for treatment-induced menopausal symptoms in breast cancer survivors: results of a randomized controlled trial. J Clin Oncol.

[22] IHI Triple Aim Initiative. Institute for Healthcare Improvement.

[23] Berwick DM, Nolan TW, Whittington J (2008). The triple aim: care, health, and cost. Health Aff (Millwood).

[24] Coles E, Wells M, Maxwell M, Harris FM, Anderson J, Gray NM, Milner G, MacGillivray S (2017). The influence of contextual factors on healthcare quality improvement initiatives: what works, for whom and in what setting? Protocol for a realist review. Syst Rev.

[25] van der Vaart R, Witting M, Riper H, Kooistra L, Bohlmeijer ET, van Gemert-Pijnen LJ (2014). Blending online therapy into regular face-to-face therapy for depression: content, ratio and preconditions according to patients and therapists using a Delphi study. BMC Psychiatry.

[26] Krijgsman J, Wolterink GK (2012). Ordening in de wereld van e-health. Nictiz.

[27] Moher D, Liberati A, Tetzlaff J, Altman DG, PRISMA Group (2009). Preferred reporting items for systematic reviews and meta-analyses: the PRISMA statement. PLoS Med.

[28] Page MJ, Moher D, Bossuyt PM, Boutron I, Hoffmann TC, Mulrow CD, Shamseer L, Tetzlaff JM, Akl EA, Brennan SE, Chou R, Glanville J, Grimshaw JM, Hróbjartsson A, Lalu MM, Li T, Loder EW, Mayo-Wilson E, McDonald S, McGuinness LA, Stewart LA, Thomas J, Tricco AC, Welch VA, Whiting P, McKenzie JE (2021). PRISMA 2020 explanation and elaboration: updated guidance and exemplars for reporting systematic reviews. BMJ.

[29] Covidence for systematic reviews. covidence.

[30] Boele FW, Verdonck-de Leeuw IM, Cuijpers P, Reijneveld JC, Heimans JJ, Klein M (2014). Internet-based guided self-help for glioma patients with depressive symptoms: design of a randomized controlled trial. BMC Neurol.

[31] Boele FW, Klein M, Verdonck-de Leeuw IM, Cuijpers P, Heimans JJ, Snijders TJ, Vos M, Bosma I, Tijssen CC, Reijneveld JC, Dutch Society for Neuro-Oncology (LWNO) (2018). Internet-based guided self-help for glioma patients with depressive symptoms: a randomized controlled trial. J Neurooncol.

[32] Bruggeman-Everts FZ, Wolvers MD, van de Schoot R, Vollenbroek-Hutten MM, Van der Lee ML (2017). Effectiveness of two Web-based interventions for chronic cancer-related fatigue compared to an active control condition: results of the "Fitter na kanker" randomized controlled trial. J Med Internet Res.

[33] Wolvers MD, Bruggeman-Everts FZ, Van der Lee ML, Van de Schoot R, Vollenbroek-Hutten MM (2015). Effectiveness, mediators, and effect predictors of Internet interventions for chronic cancer-related fatigue: the design and an analysis plan of a 3-armed randomized controlled trial. JMIR Res Protoc.

[34] Schellekens M, Wolvers M, Compen F, Bisseling E, Bruggeman-Everts F, Vollenbroek-Hutten M, Speckens A, van der Lee M (2021). Online mindfulness-based cognitieve therapie bij kanker. Tijdschrift voor Gedragstherapie.

[35] Duursma F, Schers HJ, Vissers KC, Hasselaar J (2011). Study protocol: optimization of complex palliative care at home via telemedicine. A cluster randomized controlled trial. BMC Palliat Care.

[36] Hoek PD, Schers HJ, Bronkhorst EM, Vissers KC, Hasselaar JG (2017). The effect of weekly specialist palliative care teleconsultations in patients with advanced cancer -a randomized clinical trial. BMC Med.

[37] Willems RA, Bolman CA, Mesters I, Kanera IM, Beaulen AA, Lechner L (2017). Short-term effectiveness of a Web-based tailored intervention for cancer survivors on quality of life, anxiety, depression, and fatigue: randomized controlled trial. Psychooncology.

[38] Willems RA, Mesters I, Lechner L, Kanera IM, Bolman CA (2017). Long-term effectiveness and moderators of a Web-based tailored intervention for cancer survivors on social and emotional functioning, depression, and fatigue: randomized controlled trial. J Cancer Surviv.

[39] Kanera IM, Bolman CA, Willems RA, Mesters I, Lechner L (2016). Lifestyle-related effects of the Web-based Kanker Nazorg Wijzer (Cancer Aftercare Guide) intervention for cancer survivors: a randomized controlled trial. J Cancer Surviv.

[40] Kanera IM, Willems RA, Bolman CA, Mesters I, Verboon P, Lechner L (2017). Long-term effects of a Web-based cancer aftercare intervention on moderate physical activity and vegetable consumption among early cancer survivors: a randomized controlled trial. Int J Behav Nutr Phys Act.

[41] van der Hout A, van Uden-Kraan CF, Holtmaat K, Jansen F, Lissenberg-Witte BI, Nieuwenhuijzen GA, Hardillo JA, Baatenburg de Jong RJ, Tiren-Verbeet NL, Sommeijer DW, de Heer K, Schaar CG, Sedee RJ, Bosscha K, van den Brekel MW, Petersen JF, Westerman M, Honings J, Takes RP, Houtenbos I, van den Broek WT, de Bree R, Jansen P, Eerenstein SE, Leemans CR, Zijlstra JM, Cuijpers P, van de Poll-Franse LV, Verdonck-de Leeuw IM (2020). Role of eHealth application Oncokompas in supporting self-management of symptoms and health-related quality of life in cancer survivors: a randomised, controlled trial. Lancet Oncol.

[42] van der Hout A, Jansen F, van Uden-Kraan CF, Coupé VM, Holtmaat K, Nieuwenhuijzen GA, Hardillo JA, de Jong RJ, Tiren-Verbeet NL, Sommeijer DW, de Heer K, Schaar CG, Sedee RJ, Bosscha K, van den Brekel MW, Petersen JF, Westerman M, Honings J, Takes RP, Houtenbos I, van den Broek WT, de Bree R, Jansen P, Eerenstein SE, Leemans CR, Zijlstra JM, Cuijpers P, van de Poll-Franse LV, Verdonck-de Leeuw IM (2021). Cost-utility of an eHealth application 'Oncokompas' that supports cancer survivors in self-management: results of a randomised controlled trial. J Cancer Surviv.

[43] van den Brink JL, Moorman PW, de Boer MF, Pruyn JF, Verwoerd CD, van Bemmel JH (2005). Involving the patient: a prospective study on use, appreciation and effectiveness of an information system in head and neck cancer care. Int J Med Inform.

[44] van den Brink JL, Moorman PW, de Boer MF, Hop WC, Pruyn JF, Verwoerd CD, van Bemmel JH (2007). Impact on quality of life of a telemedicine system supporting head and neck cancer patients: a controlled trial during the postoperative period at home. J Am Med Inform Assoc.

[45] Cuypers M, Lamers RE, Kil PJ, van de Poll-Franse LV, de Vries M (2018). Impact of a Web-based prostate cancer treatment decision aid on patient-reported decision process parameters: results from the Prostate Cancer Patient Centered Care trial. Support Care Cancer.

[46] Compen F, Bisseling E, Schellekens M, Donders R, Carlson L, van der Lee M, Speckens A (2018). Face-to-face and Internet-based mindfulness-based cognitive therapy compared with treatment as usual in reducing psychological distress in patients with cancer: a multicenter randomized controlled trial. J Clin Oncol.

[47] van den Berg SW, Gielissen MF, Custers JA, van der Graaf WT, Ottevanger PB, Prins JB (2015). BREATH: Web-based self-management for psychological adjustment after primary breast cancer--results of a multicenter randomized controlled trial. J Clin Oncol.

[48] van Helmondt SJ, van der Lee ML, van Woezik RA, Lodder P, de Vries J (2020). No effect of CBT-based online self-help training to reduce fear of cancer recurrence: first results of the CAREST multicenter randomized controlled trial. Psychooncology.

[49] Golsteijn RH, Bolman C, Volders E, Peels DA, de Vries H, Lechner L (2018). Short-term efficacy of a computer-tailored physical activity intervention for prostate and colorectal cancer patients and survivors: a randomized controlled trial. Int J Behav Nutr Phys Act.

[50] van Bruinessen IR, van Weel-Baumgarten EM, Gouw H, Zijlstra JM, van Dulmen S (2016). An integrated process and outcome evaluation of a Web-based communication tool for patients with malignant lymphoma: randomized controlled trial. J Med Internet Res.

[51] Admiraal JM, van der Velden AW, Geerling JI, Burgerhof JG, Bouma G, Walenkamp AM, de Vries EG, Schröder CP, Reyners AK (2017). Web-based tailored psychoeducation for breast cancer patients at the onset of the survivorship phase: a multicenter randomized controlled trial. J Pain Symptom Manage.

[52] Hummel SB, van Lankveld JJ, Oldenburg HS, Hahn DE, Kieffer JM, Gerritsma MA, Kuenen MA, Bijker N, Borgstein PJ, Heuff G, Lopes Cardozo AM, Plaisier PW, Rijna H, van der Meij S, van Dulken EJ, Vrouenraets BC, Broomans E, Aaronson NK (2017). Efficacy of Internet-based cognitive behavioral therapy in improving sexual functioning of breast cancer survivors: results of a randomized controlled trial. J Clin Oncol.

[53] Hummel SB, van Lankveld JJ, Oldenburg HS, Hahn DE, Kieffer JM, Gerritsma MA, Kuenen MA, Bijker N, Borgstein PJ, Heuff G, Cardozo AM, Plaisier PW, Rijna H, van der Meij S, van Dulken EJ, Vrouenraets BC, Broomans E, Aaronson NK (2018). Internet-based cognitive behavioral therapy realizes long-term improvement in the sexual functioning and body image of breast cancer survivors. J Sex Marital Ther.

[54] Knegtmans MF, Wauben LS, Wagemans MF, Oldenmenger WH (2020). Home telemonitoring improved pain registration in patients with cancer. Pain Pract.

[55] Verbeek JG, Atema V, Mewes JC, van Leeuwen M, Oldenburg HS, van Beurden M, Hunter MS, van Harten WH, Aaronson NK, Retèl VP (2019). Cost-utility, cost-effectiveness, and budget impact of Internet-based cognitive behavioral therapy for breast cancer survivors with treatment-induced menopausal symptoms. Breast Cancer Res Treat.

[56] Gehring K, Kloek CJ, Aaronson NK, Janssen KW, Jones LW, Sitskoorn MM, Stuiver MM (2018). Feasibility of a home-based exercise intervention with remote guidance for patients with stable grade II and III gliomas: a pilot randomized controlled trial. Clin Rehabil.

[57] Gehring K, Stuiver MM, Visser E, Kloek C, van den Bent M, Hanse M, Tijssen C, Rutten GJ, Taphoorn MJ, Aaronson NK, Sitskoorn MM (2020). A pilot randomized controlled trial of exercise to improve cognitive performance in patients with stable glioma: a proof of concept. Neuro Oncol.

[58] Visser A, Prins JB, Jansen L, Radema SA, Schlooz MS, van Dalen T, van Laarhoven HW (2018). Group medical consultations (GMCs) and tablet-based online support group sessions in the follow-up of breast cancer: a multicenter randomized controlled trial. Breast.

[59] Willems RA, Lechner L, Verboon P, Mesters I, Kanera IM, Bolman CA (2017). Working mechanisms of a Web-based self-management intervention for cancer survivors: a randomised controlled trial. Psychol Health.

[60] Willems RA, Bolman CA, Mesters I, Kanera IM, Beaulen AA, Lechner L (2015). The Kanker Nazorg Wijzer (Cancer Aftercare Guide) protocol: the systematic development of a Web-based computer tailored intervention providing psychosocial and lifestyle support for cancer survivors. BMC Cancer.

[61] Kanera IM, Willems RA, Bolman CA, Mesters I, Zambon V, Gijsen BC, Lechner L (2016). Use and appreciation of a tailored self-management eHealth intervention for early cancer survivors: process evaluation of a randomized controlled trial. J Med Internet Res.

[62] Duineveld LA, Wieldraaijer T, Wind J, Verdonck-de Leeuw IM, van Weert HC, van Uden-Kraan CF (2016). Primary care-led survivorship care for patients with colon cancer and the use of eHealth: a qualitative study on perspectives of general practitioners. BMJ Open.

[63] Duman-Lubberding S, van Uden-Kraan CF, Peek N, Cuijpers P, Leemans CR, Verdonck-de Leeuw IM (2015). An eHealth application in head and neck cancer survivorship care: health care professionals' perspectives. J Med Internet Res.

[64] Duman-Lubberding S, van Uden-Kraan CF, Jansen F, Witte BI, van der Velden LA, Lacko M, Cuijpers P, Leemans CR, Verdonck-de Leeuw IM (2016). Feasibility of an eHealth application "OncoKompas" to improve personalized survivorship cancer care. Support Care Cancer.

[65] Lubberding S, van Uden-Kraan CF, Te Velde EA, Cuijpers P, Leemans CR, Verdonck-de Leeuw IM (2015). Improving access to supportive cancer care through an eHealth application: a qualitative needs assessment among cancer survivors. J Clin Nurs.

[66] van der Hout A, van Uden-Kraan CF, Witte BI, Coupé VM, Jansen F, Leemans CR, Cuijpers P, van de Poll-Franse LV, Verdonck-de Leeuw IM (2017). Efficacy, cost-utility and reach of an eHealth self-management application 'Oncokompas' that helps cancer survivors to obtain optimal supportive care: study protocol for a randomised controlled trial. Trials.

[67] Schuit A, Holtmaat K, Hooghiemstra N, Jansen F, Lissenberg-Witte B, Coupé V, van Linde M, Becker-Commissaris A, Reijneveld J, Zijlstra-Baalbergen J, Sommeijer D, Eerenstein S, Verdonck-de Leeuw I (2019). Efficacy and cost-utility of the eHealth self-management application 'Oncokompas' tailored to patients with incurable cancer: study protocol of a randomized controlled trial. Support Care Cancer.

[68] Duineveld LA, Wieldraaijer T, van Asselt KM, Nugteren IC, Donkervoort SC, van de Ven AW, Smits AB, van Geloven AA, Bemelman WA, Beverdam FH, van Tets WF, Govaert MJ, Bosmans JE, Verdonck-de Leeuw IM, van Uden-Kraan CF, van Weert HC, Wind J (2015). Improving care after colon cancer treatment in The Netherlands, personalised care to enhance quality of life (I CARE study): study protocol for a randomised controlled trial. Trials.

[69] Boele FW, van Uden-Kraan CF, Hilverda K, Reijneveld JC, Cleijne W, Klein M, Verdonck-de Leeuw IM (2016). Attitudes and preferences toward monitoring symptoms, distress, and quality of life in glioma patients and their informal caregivers. Support Care Cancer.

[70] Melissant HC, Verdonck-de Leeuw IM, Lissenberg-Witte BI, Konings IR, Cuijpers P, Van Uden-Kraan CF (2018). 'Oncokompas', a Web-based self-management application to support patient activation and optimal supportive care: a feasibility study among breast cancer survivors. Acta Oncol.

[71] van der Hout A, Holtmaat K, Jansen F, Lissenberg-Witte BI, van Uden-Kraan CF, Nieuwenhuijzen GA, Hardillo JA, Baatenburg de Jong RJ, Tiren-Verbeet NL, Sommeijer DW, de Heer K, Schaar CG, Sedee RJ, Bosscha K, van den Brekel MW, Petersen JF, Westerman M, Honings J, Takes RP, Houtenbos I, van den Broek WT, de Bree R, Jansen P, Eerenstein SE, Leemans CR, Zijlstra JM, Cuijpers P, van de Poll-Franse LV, Verdonck-de Leeuw IM (2021). The eHealth self-management application 'Oncokompas' that supports cancer survivors to improve health-related quality of life and reduce symptoms: which groups benefit most?. Acta Oncol.

[72] van den Brink JL, Moorman PW, de Boer MF, van Bemmel JH, Pruyn JF, Verwoerd CD (2003). An information system to support the care for head and neck cancer patients. Support Care Cancer.

[73] Mujcic A, Blankers M, Boon B, Engels R, van Laar M (2018). Internet-based self-help smoking cessation and alcohol moderation interventions for cancer survivors: a study protocol of two RCTs. BMC Cancer.

[74] van de Wiel HJ, Stuiver MM, May AM, van Grinsven S, Aaronson NK, Retèl VP, Oldenburg HS, van der Poel HG, Horenblas S, van Harten WH, Groen WG (2018). (Cost-)effectiveness of an Internet-based physical activity support program (with and without physiotherapy counselling) on physical activity levels of breast and prostate cancer survivors: design of the PABLO trial. BMC Cancer.

[75] Kuijpers W, Groen WG, Loos R, Oldenburg HS, Wouters MW, Aaronson NK, van Harten WH (2015). An interactive portal to empower cancer survivors: a qualitative study on user expectations. Support Care Cancer.

[76] Groen WG, Kuijpers W, Oldenburg HS, Wouters MW, Aaronson NK, van Harten WH (2017). Supporting lung cancer patients with an interactive patient portal: feasibility study. JMIR Cancer.

[77] Kuijpers W, Groen WG, Oldenburg HS, Wouters MW, Aaronson NK, van Harten WH (2016). eHealth for breast cancer survivors: use, feasibility and impact of an interactive portal. JMIR Cancer.

[78] Kuijpers W, Groen WG, Oldenburg HS, Wouters MW, Aaronson NK, van Harten WH (2015). Development of MijnAVL, an interactive portal to empower breast and lung cancer survivors: an iterative, multi-stakeholder approach. JMIR Res Protoc.

[79] Cuypers M, Lamers RE, Kil PJ, The R, Karssen K, van de Poll-Franse LV, de Vries M (2019). A global, incremental development method for a Web-based prostate cancer treatment decision aid and usability testing in a Dutch clinical setting. Health Informatics J.

[80] Lamers RE, Cuypers M, de Vries M, van de Poll-Franse LV, Ruud Bosch J, Kil PJ (2017). How do patients choose between active surveillance, radical prostatectomy, and radiotherapy? The effect of a preference-sensitive decision aid on treatment decision making for localized prostate cancer. Urol Oncol.

[81] Cuypers M, Lamers RE, Kil PJ, van de Poll-Franse LV, de Vries M (2015). Impact of a Web-based treatment decision aid for early-stage prostate cancer on shared decision-making and health outcomes: study protocol for a randomized controlled trial. Trials.

[82] Cuypers M, Lamers RE, Kil PJ, van Tol-Geerdink JJ, van Uden-Kraan CF, van de Poll-Franse LV, de Vries M (2019). Uptake and usage of an online prostate cancer treatment decision aid in Dutch clinical practice: a quantitative analysis from the Prostate Cancer Patient Centered Care trial. Health Informatics J.

[83] Compen FR, Bisseling EM, Van der Lee ML, Adang EM, Donders AR, Speckens AE (2015). Study protocol of a multicenter randomized controlled trial comparing the effectiveness of group and individual internet-based Mindfulness-Based Cognitive Therapy with treatment as usual in reducing psychological distress in cancer patients: the BeMind study. BMC Psychol.

[84] Wolvers MD, Vollenbroek-Hutten MM (2015). An mHealth intervention strategy for physical activity coaching in cancer survivors. Personalization and Adaptation in Technology for Health Workshop held in conjunction with 23rd Conference on User Modelling, Adaptation and Personalization.

[85] van den Berg SW, Peters EJ, Kraaijeveld JF, Gielissen MF, Prins JB (2013). Usage of a generic Web-based self-management intervention for breast cancer survivors: substudy analysis of the BREATH trial. J Med Internet Res.

[86] van den Berg SW, Gielissen MF, Ottevanger PB, Prins JB (2012). Rationale of the BREAst cancer e-healTH [BREATH] multicentre randomised controlled trial: an Internet-based self-management intervention to foster adjustment after curative breast cancer by decreasing distress and increasing empowerment. BMC Cancer.

[87] van Helmondt SJ, van der Lee ML, de Vries J (2016). Study protocol of the CAREST-trial: a randomised controlled trial on the (cost-) effectiveness of a CBT-based online self-help training for fear of cancer recurrence in women with curatively treated breast cancer. BMC Cancer.

[88] Abrahams HJ, Gielissen MF, Goedendorp MM, Berends T, Peters ME, Poort H, Verhagen CA, Knoop H (2015). A randomized controlled trial of Web-based cognitive behavioral therapy for severely fatigued breast cancer survivors (CHANGE-study): study protocol. BMC Cancer.

[89] Ter Stege JA, Woerdeman LA, Hahn DE, van Huizum MA, van Duijnhoven FH, Kieffer JM, Retèl VP, Sherman KA, Witkamp AJ, Oldenburg HS, Bleiker EM (2019). The impact of an online patient decision aid for women with breast cancer considering immediate breast reconstruction: study protocol of a multicenter randomized controlled trial. BMC Med Inform Decis Mak.

[90] Garvelink MM, ter Kuile MM, Fischer MJ, Louwé LA, Hilders CG, Kroep JR, Stiggelbout AM (2013). Development of a decision aid about fertility preservation for women with breast cancer in The Netherlands. J Psychosom Obstet Gynaecol.

[91] Golsteijn RH, Bolman C, Volders E, Peels DA, de Vries H, Lechner L (2017). Development of a computer-tailored physical activity intervention for prostate and colorectal cancer patients and survivors: OncoActive. BMC Cancer.

[92] van Bruinessen IR, van Weel-Baumgarten EM, Snippe HW, Gouw H, Zijlstra JM, van Dulmen S (2014). Active patient participation in the development of an online intervention. JMIR Res Protoc.

[93] Tamminga SJ, van Hezel S, de Boer AG, Frings-Dresen MH (2016). Enhancing the return to work of cancer survivors: development and feasibility of the nurse-led eHealth intervention Cancer@Work. JMIR Res Protoc.

[94] Tamminga SJ, Hoving JL, Frings-Dresen MH, de Boer AG (2016). Cancer@Work - a nurse-led, stepped-care, e-health intervention to enhance the return to work of patients with cancer: study protocol for a randomized controlled trial. Trials.

[95] Noordman J, Driesenaar JA, van Bruinessen IR, Portielje JE, van Dulmen S (2019). Evaluation and implementation of ListeningTime: a Web-based preparatory communication tool for elderly patients with cancer and their health care providers. JMIR Cancer.

[96] Noordman J, Driesenaar JA, van Bruinessen IR, van Dulmen S (2017). ListeningTime; participatory development of a Web-based preparatory communication tool for elderly cancer patients and their healthcare providers. Internet Interv.

[97] Arts LP, van de Poll-Franse LV, van den Berg SW, Prins JB, Husson O, Mols F, Brands-Nijenhuis AV, Tick L, Oerlemans S (2017). Lymphoma InterVEntion (LIVE) - patient-reported outcome feedback and a Web-based self-management intervention for patients with lymphoma: study protocol for a randomised controlled trial. Trials.

[98] Arts L, Oerlemans S, van den Berg S, Prins J, van de Poll-Franse L (2016). Participation and characterization of patients with lymphoma in a Web-based self-management intervention. Psycho Oncol.

[99] van Eenbergen MC, van den Hurk C, Mols F, van de Poll-Franse LV (2019). Usability of an online application for reporting the burden of side effects in cancer patients. Support Care Cancer.

[100] den Bakker CM, Huirne JA, Schaafsma FG, de Geus C, Bonjer HJ, Anema JR (2019). Electronic health program to empower patients in returning to normal activities after colorectal surgical procedures: mixed-methods process evaluation alongside a randomized controlled trial. J Med Internet Res.

[101] den Bakker CM, Schaafsma FG, van der Meij E, Meijerink WJ, van den Heuvel B, Baan AH, Davids PH, Scholten PC, van der Meij S, van Baal WM, van Dalsen AD, Lips DJ, van der Steeg JW, Leclercq WK, Geomini PM, Consten EC, Schraffordt Koops SE, de Castro SM, van Kesteren PJ, Cense HA, Stockmann HB, Ten Cate AD, Bonjer HJ, Huirne JA, Anema JR (2019). Electronic health program to empower patients in returning to normal activities after general surgical and gynecological procedures: intervention mapping as a useful method for further development. J Med Internet Res.

[102] Atema V, van Leeuwen M, Oldenburg HS, van Beurden M, Hunter MS, Aaronson NK (2017). An Internet-based cognitive behavioral therapy for treatment-induced menopausal symptoms in breast cancer survivors: results of a pilot study. Menopause.

[103] Atema V, van Leeuwen M, Oldenburg HS, Retèl V, van Beurden M, Hunter MS, Aaronson NK (2016). Design of a randomized controlled trial of Internet-based cognitive behavioral therapy for treatment-induced menopausal symptoms in breast cancer survivors. BMC Cancer.

[104] van Veen MR, Beijer S, Adriaans AM, Vogel-Boezeman J, Kampman E (2015). Development of a website providing evidence-based information about nutrition and cancer: fighting fiction and supporting facts online. JMIR Res Protoc.

[105] Sungur H, Yılmaz NG, Chan BM, van den Muijsenbergh ME, van Weert JC, Schouten BC (2020). Development and evaluation of a digital intervention for fulfilling the needs of older migrant patients with cancer: user-centered design approach. J Med Internet Res.

[106] Yılmaz NG, Sungur H, van Weert JC, van den Muijsenbergh ME, Schouten BC (2020). Enhancing patient participation of older migrant cancer patients: needs, barriers, and eHealth. Ethn Health (forthcoming).

[107] (2020). Health Software — Part 2 Health and wellness apps - Quality and reliability. International Organization for Standardization.

[108] Over Nictiz. Nictiz.

[109] Struijs JN, Drewes HW, Heijink R, Baan CA (2015). How to evaluate population management? Transforming the Care Continuum Alliance population health guide toward a broadly applicable analytical framework. Health Policy.

[110] Hendrikx RJ, Drewes HW, Spreeuwenberg M, Ruwaard D, Struijs JN, Baan CA (2016). Which Triple Aim related measures are being used to evaluate population management initiatives? An international comparative analysis. Health Policy.

[111] (2018). Quality Assessment Tool for Quantitative Studies. Effective Public Healthcare Panacea Project.

[112] Thomas BH, Ciliska D, Dobbins M, Micucci S (2004). A process for systematically reviewing the literature: providing the research evidence for public health nursing interventions. Worldviews Evid Based Nurs.

[113] Jackson N, Waters E, Guidelines for Systematic Reviews in Health Promotion and Public Health Taskforce (2005). Criteria for the systematic review of health promotion and public health interventions. Health Promot Int.

[114] Seiler A, Klaas V, Tröster G, Fagundes CP (2017). eHealth and mHealth interventions in the treatment of fatigued cancer survivors: a systematic review and meta-analysis. Psychooncology.

[115] Kleinpeter E (2017). Four ethical issues of “E-Health”. IRBM.

[116] Mock V, Atkinson A, Barsevick A, Cella D, Cimprich B, Cleeland C, Donnelly J, Eisenberger MA, Escalante C, Hinds P, Jacobsen PB, Kaldor P, Knight SJ, Peterman A, Piper BF, Rugo H, Sabbatini P, Stahl C, National Comprehensive Cancer Network (2000). NCCN practice guidelines for cancer-related fatigue. Oncology (Williston Park).

[117] Mehnert A, Hartung TJ, Friedrich M, Vehling S, Brähler E, Härter M, Keller M, Schulz H, Wegscheider K, Weis J, Koch U, Faller H (2018). One in two cancer patients is significantly distressed: prevalence and indicators of distress. Psychooncology.

[118] van den Beuken-van Everdingen MH, Hochstenbach LM, Joosten EA, Tjan-Heijnen VC, Janssen DJ (2016). Update on prevalence of pain in patients with cancer: systematic review and meta-analysis. J Pain Symptom Manage.

[119] Penedo FJ, Oswald LB, Kronenfeld JP, Garcia SF, Cella D, Yanez B (2020). The increasing value of eHealth in the delivery of patient-centred cancer care. Lancet Oncol.

[120] Xie B (2011). Older adults, e-health literacy, and collaborative learning: an experimental study. J Am Soc Inf Sci Technol.

[121] Hoogenbosch B, Postma J, de Man-van Ginkel JM, Tiemessen NA, van Delden JJ, van Os-Medendorp H (2018). Use and the users of a patient portal: cross-sectional study. J Med Internet Res.

[122] Vreugdenhil MM, Ranke S, de Man Y, Haan MM, Kool RB (2019). Patient and health care provider experiences with a recently introduced patient portal in an academic hospital in the Netherlands: mixed methods study. J Med Internet Res.

[123] Verstraete E, Koehorst AM, van Os-Medendorp H (2016). [Does the patient benefit from real-time access to one's electronic record? Evaluation of the patient portal in University Medical Centre Utrecht, the Netherlands]. Ned Tijdschr Geneeskd.

[124] Spil TA, Katsma CP, Stegwee RA, Albers EF, Freriks A, Ligt E (2010). Value, participation and quality of electronic health records in the Netherlands. Proceedings of the 43rd Hawaii International Conference on System Sciences.

[125] Chen YY, Guan BS, Li ZK, Li XY (2018). Effect of telehealth intervention on breast cancer patients' quality of life and psychological outcomes: a meta-analysis. J Telemed Telecare.

[126] Xu A, Wang Y, Wu X (2019). Effectiveness of e-health based self-management to improve cancer-related fatigue, self-efficacy and quality of life in cancer patients: systematic review and meta-analysis. J Adv Nurs.

[127] Wang Y, Lin Y, Chen J, Wang C, Hu R, Wu Y (2020). Effects of Internet-based psycho-educational interventions on mental health and quality of life among cancer patients: a systematic review and meta-analysis. Support Care Cancer.

[128] Pingree S, Hawkins R, Baker T, duBenske L, Roberts LJ, Gustafson DH (2010). The value of theory for enhancing and understanding e-health interventions. Am J Prev Med.

[129] Griebel L, Kolominsky-Rabas P, Schaller S, Siudyka J, Sierpinski R, Papapavlou D, Simeonidou A, Prokosch H, Sedlmayr M (2017). Acceptance by laypersons and medical professionals of the personalized eHealth platform, eHealthMonitor. Inform Health Soc Care.

[130] Ossebaard HC, Van Gemert-Pijnen L (2016). eHealth and quality in health care: implementation time. Int J Qual Health Care.

[131] Huygens MW (2018). A patient perspective on eHealth in primary care: critical reflections on the implementation and use of online care services. Universitaire Pers Maastricht.

[132] van Gemert-Pijnen L, Kip H, Kelders SM, Sanderman R, van Gemert-Pijnen L, Kelders SM, Kip H, Sanderman R (2018). Introducing eHealth. eHealth Research, Theory and Development: A Multidisciplinary Approach.

[133] Ehrismann M, Stegwee R (2015). Trust in eHealth Services - A Practice Driven Review of the Literature. Capgemini Consulting.

[134] Soloe C, Burrus O, Subramanian S (2021). The effectiveness of mHealth and eHealth tools in improving provider knowledge, confidence, and behaviors related to cancer detection, treatment, and survivorship care: a systematic review. J Cancer Educ.

[135] Dávalos ME, French MT, Burdick AE, Simmons SC (2009). Economic evaluation of telemedicine: review of the literature and research guidelines for benefit-cost analysis. Telemed J E Health.

[136] Casal JP, Ramos AC (2017). E-government policies in health care: the social cost of digitalization. Proceedings of 17th European Conference on Digital Government.

[137] Somers TJ, Kelleher SA, Westbrook KW, Kimmick GG, Shelby RA, Abernethy AP, Keefe FJ (2016). A small randomized controlled pilot trial comparing mobile and traditional pain coping skills training protocols for cancer patients with pain. Pain Res Treat.

[138] Jibb LA, Stevens BJ, Nathan PC, Seto E, Cafazzo JA, Johnston DL, Hum V, Stinson JN (2017). Implementation and preliminary effectiveness of a real-time pain management smartphone app for adolescents with cancer: a multicenter pilot clinical study. Pediatr Blood Cancer.

