# Supplementary material for: eHealth Interventions for Dutch Cancer Care: Systematic Review Using the Triple Aim Lens
Source: JMIR Cancer. 2022 Jun 14;8(2):e37093. doi: 10.2196/37093 (PMC9240931; doi:10.2196/37093)
Supplement: Multimedia Appendix 1 [file cancer_v8i2e37093_app1.docx]

# **Multimedia Appendix 1.** **Overview of search strategies per database**

**PubMed (results from 10-06-2021)**

("Telemedicine"[majr] OR "telemed*"[ti] OR "teleconference"[ti] OR "teleconsult*"[ti] OR "telecommunication"[ti] OR "telehealth"[ti] OR "tele-health"[ti] OR "tele health"[ti] OR "telecare"[ti] OR "tele-care"[ti] OR "tele care"[ti] OR "electronic health"[ti] OR "mobile health"[ti] OR "mHealth"[ti] OR "eHealth"[ti] OR "m-Health"[ti] OR "e-Health"[ti] OR "telephone"[ti] OR "mobile phone*"[ti] OR "cell phone*"[ti] OR "cellular phone*"[ti] OR "smartphone*"[ti] OR "smart phone*"[ti] OR "mobile technology" OR "wireless"[ti] OR "internet"[ti] OR "Internet"[majr] OR "internet-based"[ti] OR "computer*"[ti] OR "computer-assisted instruction"[ti] OR "multimedia"[ti] OR "email*"[ti] OR "e-mail*"[ti] OR "web"[ti] OR "website*"[ti] OR "web based"[ti] OR "web-based"[ti] OR "online"[ti] OR "on-line"[ti] OR "app"[ti] OR "apps"[ti] OR "digital"[ti] OR "text messag*"[ti] OR "SMS"[ti] OR "short message service"[ti] OR "remote consult*"[ti] OR "telemonitoring"[ti] OR "iphone*"[ti] OR "i-phone*"[ti] OR "virtual community"[ti] OR "home monitor*"[ti] OR "health information technology"[ti] OR "health information systems"[ti] OR "interactive health communication"[ti] OR "patient portal"[ti] OR "webbased"[ti] OR "web-based"[ti] OR "webpage*"[ti] OR "digital decision*"[ti]) AND ("Neoplasms"[Mesh] OR "Neoplas*"[tw] OR "Tumor*"[tw] OR "Tumour*"[tw] OR "Cancer*"[tw] OR "malignan*"[tw] OR "oncolog*"[tw] OR "carcinoma*"[tw] OR "adenoma*"[tw] OR "Medical Oncology"[Mesh]) AND ("Netherlands"[Mesh] OR "Netherlands"[tiab] OR "Holland"[tiab] OR "Dutch"[tiab] OR "Benelux"[tw])

**Cochrane (results from 10-06-2021)**

Record title:

("telemed*" OR "teleconference" OR "teleconsult*" OR "telecommunication" OR "telehealth" OR "tele-health" OR "tele health" OR "telecare" OR "tele-care" OR "tele care" OR "electronic health" OR "mobile health" OR "mHealth" OR "eHealth" OR "m-Health" OR "e-Health" OR "telephone" OR "mobile phone*" OR "cell phone*" OR "cellular phone*" OR "smartphone*" OR "smart phone*" OR "mobile technology" OR "wireless" OR "internet" OR "internet-based" OR "computer*" OR "computer-assisted instruction" OR "multimedia" OR "email*" OR "e-mail*" OR "web" OR "website*" OR "web based" OR "web-based" OR "online" OR "on-line" OR "app" OR "apps" OR "digital" OR "text messag*" OR "SMS" OR "short message service" OR "remote consult*" OR "telemonitoring" OR "iphone*" OR "i-phone*" OR "virtual community" OR "home monitor*" OR "health information technology" OR "health information systems" OR "interactive health communication" OR "patient portal" OR "webbased" OR "web-based" OR "webpage*" OR "digital decision*")

AND

Title, abstract, keywords :

("Neoplas*" OR "Tumor*" OR "Tumour*" OR "Cancer*" OR "malignan*" OR "oncolog*" OR "carcinoma*" OR "adenoma*") AND ("Netherlands" OR "Holland" OR "Dutch" OR "Benelux")

(DE ("Telemedicine" OR "Online Therapy" OR "Teleconferencing" OR "Teleconsultation" OR "Telepsychiatry" OR "Telepsychology" OR "Telerehabilitation" OR "Internet" OR "Online Therapy" OR "Smartphones" OR "Mobile Phones" OR "Text Messaging" OR "Websites" OR "Health Information Technology" OR "Decision Support Systems") OR TI ("telemed*" OR "teleconference" OR "teleconsult*" OR "telecommunication" OR "telehealth" OR "tele-health" OR "tele health" OR "telecare" OR "tele-care" OR "tele care" OR "electronic health" OR "mobile health" OR "mHealth" OR "eHealth" OR "m-Health" OR "e-Health" OR "telephone" OR "mobile phone*" OR "cell phone*" OR "cellular phone*" OR "smartphone*" OR "smart phone*" OR "mobile technology" OR "wireless" OR "internet" OR "internet-based" OR "computer*" OR "computer-assisted instruction" OR "multimedia" OR "email*" OR "e-mail*" OR "web" OR "website*" OR "web based" OR "web-based" OR "online" OR "on-line" OR "app" OR "apps" OR "digital" OR "text messag*" OR "SMS" OR "short message service" OR "remote consult*" OR "telemonitoring" OR "iphone*" OR "i-phone*" OR "virtual community" OR "home monitor*" OR "health information technology" OR "health information systems" OR "interactive health communication" OR "patient portal" OR "webbased" OR "web-based" OR "webpage*" OR "digital decision*")) AND (DE ("Neoplasms" OR "Benign Neoplasms" OR "Breast Neoplasms" OR "Endocrine Neoplasms" OR "Leukemias" OR "Melanoma" OR "Metastasis" OR "Nervous System Neoplasms" OR "Terminal Cancer") OR TX ("Neoplas*" OR "Tumor*" OR "Tumour*" OR "Cancer*" OR "malignan*" OR "oncolog*" OR "carcinoma*" OR "adenoma*"))

**PsychInfo (results from 14-06-2021)**

(DE ("Telemedicine" OR "Online Therapy" OR "Teleconferencing" OR "Teleconsultation" OR "Telepsychiatry" OR "Telepsychology" OR "Telerehabilitation" OR "Internet" OR "Online Therapy" OR "Smartphones" OR DE "Mobile Phones" OR "Text Messaging" OR "Websites" OR "Health Information Technology" OR "Decision Support Systems") OR TI ("telemed*" OR "teleconference" OR "teleconsult*" OR "telecommunication" OR "telehealth" OR "tele-health" OR "tele health" OR "telecare" OR "tele-care" OR "tele care" OR "electronic health" OR "mobile health" OR "mHealth" OR "eHealth" OR "m-Health" OR "e-Health" OR "telephone" OR "mobile phone*" OR "cell phone*" OR "cellular phone*" OR "smartphone*" OR "smart phone*" OR "mobile technology" OR "wireless" OR "internet" OR "internet-based" OR "computer*" OR "computer-assisted instruction" OR "multimedia" OR "email*" OR "e-mail*" OR "web" OR "website*" OR "web based" OR "web-based" OR "online" OR "on-line" OR "app" OR "apps" OR "digital" OR "text messag*" OR "SMS" OR "short message service" OR "remote consult*" OR "telemonitoring" OR "iphone*" OR "i-phone*" OR "virtual community" OR "home monitor*" OR "health information technology" OR "health information systems" OR "interactive health communication" OR "patient portal" OR "webbased" OR "web-based" OR "webpage*" OR "digital decision*")) AND (DE ("Neoplasms" OR "Benign Neoplasms" OR "Breast Neoplasms" OR "Endocrine Neoplasms" OR "Leukemias" OR "Melanoma" OR "Metastasis" OR "Nervous System Neoplasms" OR "Terminal Cancer") OR TX ("Neoplas*" OR "Tumor*" OR "Tumour*" OR "Cancer*" OR "malignan*" OR "oncolog*" OR "carcinoma*" OR "adenoma*")) AND (TI ("Netherlands" OR "Holland" OR "Dutch" OR "Benelux") OR AB ("Netherlands" OR "Holland" OR "Dutch" OR "Benelux"))

**WebofScience (results from 10-06-2021)**

TI=("telemed*" OR "teleconference" OR "teleconsult*" OR "telecommunication" OR "telehealth" OR "tele-health" OR "tele health" OR "telecare" OR "tele-care" OR "tele care" OR "electronic health" OR "mobile health" OR "mHealth" OR "eHealth" OR "m-Health" OR "e-Health" OR "telephone" OR "mobile phone*" OR "cell phone*" OR "cellular phone*" OR "smartphone*" OR "smart phone*" OR "mobile technology" OR "wireless" OR "internet" OR "internet-based" OR "computer*" OR "computer-assisted instruction" OR "multimedia" OR "email*" OR "e-mail*" OR "web" OR "website*" OR "web based" OR "web-based" OR "online" OR "on-line" OR "app" OR "apps" OR "digital" OR "text messag*" OR "SMS" OR "short message service" OR "remote consult*" OR "telemonitoring" OR "iphone*" OR "i-phone*" OR "virtual community" OR "home monitor*" OR "health information technology" OR "health information systems" OR "interactive health communication" OR "patient portal" OR "webbased" OR "web-based" OR "webpage*" OR "digital decision*") AND TS=("Neoplas*" OR "Tumor*" OR "Tumour*" OR "Cancer*" OR "malignan*" OR "oncolog*" OR "carcinoma*" OR "adenoma*") AND TS=("Netherlands" OR "Holland" OR "Dutch" OR "Benelux")
