# Supplementary material for: eHealth Interventions for Dutch Cancer Care: Systematic Review Using the Triple Aim Lens
Source: JMIR Cancer. 2022 Jun 14;8(2):e37093. doi: 10.2196/37093 (PMC9240931; doi:10.2196/37093)
Supplement: Multimedia Appendix 3 [file cancer_v8i2e37093_app3.docx]

# Multimedia Appendix 3. Characteristics of e-health interventions for cancer care in the Netherlands

| **Intervention** | **Intervention summary** | **Function** | **Type of  e-health** | **Support of healthcare professionals** | **Stakeholders involvement** | **Setting^1^** | **Target population** | **Scientific theories** |
| --- | --- | --- | --- | --- | --- | --- | --- | --- |
| Cancer Aftercare Guide (Kanker Nazorg Wijzer) [1-7] | Online training modules to provide cancer survivors personalised information on psychosocial and lifestyle issues to improve QoL | Inform and treat | Web applications/ web portals | No | Focus group interviews and a survey study during the development phase [4] | 2 | All cancer survivors who completed primary treatment | PST, SCT, self-regulation and behavioural change strategies |
| OncoCompass (OncoKompas) [8-19] | A web-based self-management application to monitor health-related QoL and support cancer survivors in finding and obtaining optimal supportive care | Inform, simple monitoring, and self-manage | Web applications/ web portals | No | Interviews with cancer survivors on unmet needs during follow-up care [11] and with HCP’s on the expected value of the intervention [9]. Cognitive walkthroughs with HCP’s to investigate perceived usability. Pretest-posttest study with cancer survivors to test feasibility [10] | 2 | All cancer survivors | Biopsychosocial model; chronic care self-management model |
| Transmural Oncologal Support (TOS) [20-22] | An electronic health information support system to support the care of H&N cancer patients after their hospital discharge. The four functions of the intervention are (1) communication amongst HCP’s and between HCP’s and patients, (2) information for HCP’s and patients, (3) contact with fellow sufferers and (4) monitoring of discharged patients with electronic surveys | Inform, communicate, and simple monitoring | Web applications/ web portals | Patients are regularly prompted to complete an electronic survey after logging in. If needed, the system automatically generates alerts. Alerts are sent to a hospital support team. The team responds to alerts following a protocol | No information provided | 2 | Head and neck cancer patients | No |
| No name. *Decision aid for metastatic colorectal cancer patients in palliative setting* [23] | A DA to assist in treatment decisions, patients' perspective and balancing harm and benefit before starting first or subsequent lines of palliative systemic treatment or best supportive care | Inform and treat | Web applications/ web portals | No | Interviews with patients and survey among HCP’s survey on information needs. Patients’ and providers’ participation and satisfaction were measured through rating and comments [23] | 2 | Metastatic colorectal cancer patients, treated with palliative intent | No |
| Everything under control (Alles onder controle) [24, 25] | An internet-based guided self-help intervention to decrease depressive symptoms | Inform and treat | Web applications/ web portals | Online support from a coach, nurse, or psychology student, consisting of feedback on completed exercises and additional support on request. | No information provided | 2 | Glioma patients and patients with hematological malignancies with depressive symptoms | CBT |
| Lungcancer.info (Longkanker .info) [26, 27] | A website to inform patients and their relatives about lung cancer | Inform and communicate | Web applications/ web portals | An interactive section on the website 'Ask the physician' supports questioners, clarifies and indicates where possibilities can be found with their specialist | No information provided | 3 | Lung cancer patients |  |
| MyCourse – Moderate Drinking (MijnKoers – Minderen met Drinken) [28] | An internet-based self-help intervention to assist cancer survivors in alcohol moderation | Inform and preventative behaviour change | Web applications/ web portals | No | Focus groups and interviews in the development phase with cancer survivors and experts on eHealth, smoking cessation and cancer survivors to suit target group’s needs [28] | 3 | All cancer survivors, excessive drinkers | CBT, motivational interviewing (MI) and ACT |
| MyCourse – Quit Smoking (MijnKoers – Stoppen met Roken) [28] | An internet-based self-help intervention to assist cancer survivors in smoking cessation | Inform and preventative behaviour change | Web applications/ web portals | No | Focus groups and interviews in the development phase with cancer survivors and experts on eHealth, alcohol misuse and cancer survivors to suit the target group’s needs [28] | 3 | All cancer survivors, smokers | CBT, MI and ACT |
| Internet-based Physical Activity Support intervention (IPAS) [29, 30] | An internet-based program to promote PA and enhance psychosocial functioning among breast and prostate cancer survivors | Inform and preventative behaviour change | Web applications/ web portals | No | Feasibility study with focus groups with cancer survivors and HCP’s to explore expectations of possible features [30] | 2 | Breast or prostate cancer survivors | Transtheoretical model (TTM) and aspects from the Theory of Planned behaviour and SCT |
| MyAVL (MijnAVL). *Antonie van Leeuwenhoek ziekenhuis is a Dutch hospital* [31-33] | An interactive portal to empower cancer patients by (1) patient education, (2) overview of past and future appointments, (3) access to the electronic medical record, (4) patient-reported outcomes and related feedback, (5) physical activity support program | Inform and self-manage | Web applications/ web portals and EHR or PHR | No | Input from professionals to the content and procedures. Interviews and usability tests with cancer survivors to evaluate first draft and prototype [33]. Two feasibility studies among breast [32] and lung cancer survivors [31] with focus groups, log data analysis and a survey | 2 | Breast and lung cancer survivors | No |
| Prostate cancer decision aid (Prostaatkanker keuzehulp) [34-38] | A DA to support the process of shared decision-making between prostate cancer patients and their clinicians | Inform and treat | Web applications/ web portals | Introduction of DA to the patient by urologist or nurse | Multidisciplinary development team. Observations and role-playing to understand treatment decision-making consultations and usability testing [34] | 2 | Prostate cancer patients | No |
| Less tired (Minder Moe) [39-41] | A web-based, therapist-guided, individual eMBCT to reduce severe fatigue in cancer survivors through mindfulness exercises | Inform, treat and communicate | Web applications/ web portals | Weekly feedback from the care provider through e-mail or telephone calls | No information provided | 2 | All cancer survivors | Mindfulness-based cognitive therapy (MBCT) |
| Less tired for anxiety/ depression complaints [39, 42, 43] | A web-based, therapist-guided, individual eMBCT to reduce anxiety or depression complaints in cancer survivors | Inform, treat, and communicate | Web applications/ web portals | No | No information provided | 2 | All cancer survivors | MBCT |
| Ambulant Activity Feedback therapy (AAF) [39, 40, 44] | A mobile intervention with ambulant activity coaching system to change PA for participants with chronic fatigue syndrome | Inform, preventative behaviour change, and communicate | Mobile applications | Supported weekly by a physiotherapist through e-mail | Interviews with HCP’s to define recommendations for the first draft [44] | 2 | All cancer patients with chronic fatigue syndrome | Cognitive Behavioural Change Principles |
| BREATH [45-47] | A web-based self-management intervention to support the psychological adjustment of women after primary treatment | Inform and self-manage | Web applications/ web portals |  | No information provided | 2 | Female BC survivors | CBT, Transactional model of stress and model of psychological well-being in cancer survivors. |
| Less fear after cancer (Minder angst bij kanker) [48, 49] | An online tailored self-help training to reduce FCR in women with curatively treated BC. | Inform, treat, and self-manage | Web applications/ web portals |  | No information provided | 2 | Female BC survivors | CBT |
| My Fit Brain (Mijn Fitte Brein) [50] | A web-based lifestyle intervention to improve cognitive functioning in patients with cancer returning to work | Inform, simple monitoring, and self-manage | Web applications/ web portals |  | No information provided | 3 | All cancer survivors who experience cognitive impairment after cancer treatment | Behaviour change techniques and GAS |
| On the road to recovery [51] | A web-based CBT to reduce fatigue severity among severely fatigued BC survivors | Inform and treat | Web applications/ web portals | Patients start with two face-to-face sessions with their therapist. Therapists will contact patients two-weekly by e-mail to give feedback on their progress, answer questions, and initiate video sessions with a secured video consultation system | Development with a team of experts. Test pilot to test usability among BC survivors [51] | 2 | Female BC survivors, severely fatigued | CBT |
| Breast Reconstruction Decision Aid (Borst-reconstructie Keuzehulp) [52] | An online DA to support decision making in women considering immediate breast reconstruction | Inform and treat | Web applications/ web portals | No | Developed with experts, ZorgkeuzeLab, and a multidisciplinary working group. Needs assessment and usability testing among patients and HCP’s [52] | 2 | Female BC patients | No |
| Breast cancer and desire for children (borstkankeren-kinderwens.nl) [53] | An online DA to inform patients about fertility preservation (FP), prepare patients for a counselling consultation about FP and enable decision-making about FP | Inform and self-manage | Web applications/ web portals | No | Consulting of patients before and after concept development. Assessment of acceptability and understandability of draft version among patients, physicians and healthy volunteers [53] | 2 | Female BC patients | No |
| OncoActive [54, 55] | A computer-tailored PA program providing PA advice to increase awareness, initiation and maintenance of PA in prostate and colorectal cancer patients and survivors | Inform and preventative behaviour change | Web applications/ web portals | No. | Interviews with the target group and HCP’s on physical activity (PA) advantages, barriers to PA and intervention preferences. Pre- and pilot tests among patients and HCP’s [54] | 3 | Prostate and colorectal cancer patients and survivors | Behavioural change theories such as social cognitive models |
| PatientTIME [56, 57] | A web-based intervention to boost patients’ self-efficacy in patient-professional communication | Inform and self-manage | Web applications/ web portals | No | Two patients were research partners. Input from patients was gathered for a needs assessment, intervention design and evaluation [56] | 2 | Patients with malignant lymphoma | Modelling, tailoring information, previsit goal setting, and listening to visit recordings |
| [Cancer@Work](mailto:Cancer@Work) [58, 59] | A personal and secure website containing various functionalities for cancer survivors and a public website for employers, occupational physicians, and general physicians to enhance the return to work | Inform and self-manage | Web applications/ web portals | The specialised nurse will (1) answer questions, (2) monitor and supervise use, (3) provide personal feedback on assignments and (4) encourage patients to comply with the intervention | Feasibility study with interviews and a questionnaire among cancer survivors, employers and occupational physicians to evaluate the intervention [58] | 2 | All cancer survivors returning to work | Problem-solving and cognitive behavioural techniques |
| ListeningTIME (Luistertijd) [60, 61] | A web-based preparatory communication tool to help cancer patients prepare clinical encounters and overcome communication barriers | Inform and self-manage | Web applications/ web portals | No. | Participatory development process with constant feedback loop [61] and usability testing with patients and HCP’s [60] | 2 | All cancer patients, 65 years or older | Principles used in PatientTime. |
| Living with lymphoma [62, 63] | A web-based self-management intervention to increase self-management skills and satisfaction with information and reduce psychological distress | Inform and self-manage | Web applications/ web portals |  | No information provided. | 2 | (non-) Hodgkin lymphoma patients | CBT |
| ENCOURAGE [64] | A web-based tailored psycho-educational program to empower patients to take control over prevailing problems | Inform and self-manage | Web applications/ web portals | Patients could contact the research psychologist (telephone/e-mail) to discuss any questions or problems | No information provided | 2 | Female BC patients in the re-entry phase | Approach-oriented coping strategies and problem-solving therapy |
| Cancer, Intimacy and Sexuality (Kanker, Intimiteit en Seksualiteit)[65-67] | An internet-based CBT program to alleviate problems with sexuality and intimacy in women who have been treated for BC | Inform and preventative behaviour change | Web applications/ web portals | The therapist and client formulated goals before the CBT, and the sexologist selected modules that best fit the problems. After each module, participants got feedback from the sexologist via e-mail. Two evaluation interviews were held by telephone | No information provided | 2 | Treated BC patients, females, 18 - 65 years, diagnosed with sexual dysfunction | CBT |
| Alongside Cancer (BijKanker) [68] | A web application which provides (1) access for patients to information on possible side effects, (2) opportunity for patients to report the burden of side effects in a digital diary, (3) patient communication with oncology nurses and (4) feedback of data to patients, oncology nurses and clinicians in the form of a personalised graph showing the course of their side effects | Inform, simple monitoring, and communicate | Web applications/ web portals | Contact with HCP's through e-consults | Usability study among patients [68] | 2 | All cancer patients | No |
| I recover (Ikherstel) [69, 70] | A care program consisting of a website, a mobile app, an activity tracker, and the possibility of an electronic consult to optimise perioperative care by providing tailored information, increasing patients’ self-management and delivering interactive communication features | Inform, self-manage, and communicate | Web applications/ web portals, mobile application and health-sensor/ health-gateway/ wearable devices | Possibility to ask questions to HCP’s of their hospital via an e-consult | Intervention mapping study to guide the development with a survey study and focus groups among stakeholders [70]. Evaluation study to evaluate implementation [69] | 2 | All patients scheduled for a laparoscopic or abdominal colorectal resection or hysterectomy (i.e. not exclusively cancer patients) | The Attitude-Social Influence-Self-Efficacy model |
| No name. *Home monitoring tool for adequate pain treatment* [71] | An automatic computer-based monitoring system for adequate pain treatment in patients with cancer by early detection of moderate to severe pain and increased adequacy of patients’ pain treatment | Simple monitoring | EHR/ PHR | When the reported NRS pain score was ≥5, a specialised oncology nurse adapted the pain treatment when necessary |  | 2 | All cancer patients | No |
| EvaOnline [72-75] | An internet-based CBT program to alleviate or reduce the impact of menopausal symptoms in BC survivors who have experienced treatment-induced menopause | Inform, self-manage, and treat | Web applications/ web portals and mobile applications | Women can receive a scheduled 30 min telephone interview prior to the program start and weekly feedback per e-mail by a trained therapist during the program | A pilot study among BC survivors and counsellors to develop, evaluate the feasibility and generate intervention efficacy data with a survey and interviews [73] | 2 | Female BC survivors | CBT-based |
| All information you've asked for (AYA4) [76] | A secure online support community to help AYA cancer patients express feelings, exchange information, address peer support, and help cope with cancer | Communicate | Web applications/ web portals and mobile applications |  | Developed with experts and cancer patients of the Radboud UMC. Study to analyse user statistics, content analysis and user evaluation [76] | 2 | Adolescent and young adult (AYA) cancer patients | No |
| Nutrition and cancer info (Voedingenkankerinfo.nl) [77] | A website to make scientific information available for the general public, cancer patients, and cancer survivors through a website | Inform and self-manage | Web applications/ web portals | No | Pretest and evaluation with a survey among cancer survivors, HCP’s and communication experts. User statistics analysis post-launch [77] | 3 | Cancer patients and survivors (and the general public) | No |
| No name. *Home-based exercise intervention* [78, 79] | A home-based exercise intervention with remote guidance to maintain or improve cognitive function by improving aerobic fitness | Self-manage, simple monitoring, and preventative behaviour change | Web applications/ web portals, mobile applications and health-sensor/ health-gateway/ wearable devices | The physiotherapist visited participants at home at the start of the intervention to give an individualised exercise prescription and instructions on how to use a sports watch with a heart rate monitor and upload training data at least once a week. The physiotherapist weekly monitored the training data on the platform and provided feedback by e-mail | No information provided | 2 | Stable, lower grade glioma patients | No |
| My-GMC [80] | A blended care intervention, combining a face-to-face GMC and an online app. Connecting patients, linking patients to professionals, and informing patients to decrease the disease’s long-term physical and psychological side effects among BC patients | Inform and communicate | Mobile applications | Blended e-health: face-to-face GMC's and three online support group sessions, where patients met the other GMC participants in the presence of a clinical nurse specialist | No information provided | 2 | BC patients | No |
| No name. *Teleconsultation for patients receiving palliative home care* [81, 82] | Weekly teleconsultations between patients receiving palliative home care and a hospital-based palliative care team to provide palliative care to home-dwelling patients to improve the patient-experienced symptom burden compared to usual care | Inform and communicate | Video-communication and mobile applications | Weekly teleconsultations with HCP's | No information provided | 2 | All cancer patients receiving palliative care | No |
| No name. *Cancer-specific Decision Aids* [83] | A web-based DA to support patients in decision making regarding future fertility | Inform and treat | Web applications/ web portals |  | Developed by a multidisciplinary steering group. Alpha testing with cancer survivors and professionals [83] | 2 | Females of reproductive age, several types of cancer patients | No |
| The conversation starter [84, 85] | A module of a web-based digital tool to resolve language barriers between non-Western patients with low Dutch language proficiency and their HCP’s to enhance patient participation | Inform and communicate | Web applications/ web portals |  | Needs assessment study [84] with focus groups, interviews and a pilot study to evaluate the intervention, both with HCP’s and Turkish-Dutch and Moroccan-Dutch cancer survivors [85] | 2 | Cancer patients with a Turkish-Dutch and Moroccan background | The Spiral Technology Action Research mode |

1: Setting 1= primary care, 2= secondary care, 3 = community setting.

## References

1. Willems, R.A., et al., *Long-term effectiveness and moderators of a web-based tailored intervention for cancer survivors on social and emotional functioning, depression, and fatigue: randomized controlled trial.* Journal of cancer survivorship, 2017. **11**(6): p. 691‐703.

2. Willems, R.A., et al., *Working mechanisms of a web-based self-management intervention for cancer survivors: A randomised controlled trial.* Psychology & Health, 2017. **32**(5): p. 605-625.

3. Willems, R.A., et al., *Short-term effectiveness of a web-based tailored intervention for cancer survivors on quality of life, anxiety, depression, and fatigue: randomized controlled trial.* Psychooncology, 2017. **26**(2): p. 222-230.

4. Willems, R.A., et al., *The Kanker Nazorg Wijzer (Cancer Aftercare Guide) protocol: the systematic development of a web-based computer tailored intervention providing psychosocial and lifestyle support for cancer survivors.* BMC Cancer, 2015. **15**: p. 580.

5. Kanera, I.M., et al., *Lifestyle-related effects of the web-based Kanker Nazorg Wijzer (Cancer Aftercare Guide) intervention for cancer survivors: a randomized controlled trial.* Journal of cancer survivorship, 2016. **10**(5): p. 883‐897.

6. Kanera, I.M., et al., *Long-term effects of a web-based cancer aftercare intervention on moderate physical activity and vegetable consumption among early cancer survivors: a randomized controlled trial.* International journal of behavioral nutrition and physical activity, 2017. **14**(1): p. 19.

7. Kanera, I.M., et al., *Use and Appreciation of a Tailored Self-Management eHealth Intervention for Early Cancer Survivors: process Evaluation of a Randomized Controlled Trial.* Journal of medical Internet research, 2016. **18**(8): p. e229.

8. Duineveld, L.A., et al., *Primary care-led survivorship care for patients with colon cancer and the use of eHealth: a qualitative study on perspectives of general practitioners.* BMJ Open, 2016. **6**(4): p. e010777.

9. Duman-Lubberding, S., et al., *An eHealth application in head and neck cancer survivorship care: health care professionals' perspectives.* Journal of medical Internet research, 2015. **17**(10): p. e4870.

10. Duman-Lubberding, S., et al., *Feasibility of an eHealth application “OncoKompas” to improve personalized survivorship cancer care.* Supportive care in cancer, 2016. **24**(5): p. 2163-2171.

11. Lubberding, S., et al., *Improving access to supportive cancer care through an e H ealth application: a qualitative needs assessment among cancer survivors.* Journal of clinical nursing, 2015. **24**(9-10): p. 1367-1379.

12. van der Hout, A., et al., *Cost-utility of an eHealth application ‘Oncokompas’ that supports cancer survivors in self-management: results of a randomised controlled trial.* Journal of Cancer Survivorship, 2021. **15**(1): p. 77-86.

13. van der Hout, A., et al., *Efficacy, cost-utility and reach of an eHealth self-management application 'Oncokompas' that helps cancer survivors to obtain optimal supportive care: study protocol for a randomised controlled trial.* Trials, 2017. **18**(1): p. 228.

14. Schuit, A., et al., *Efficacy and cost-utility of the ehealth selfmanagement application 'oncokompas' tailored to patients with incurable cancer: study protocol of a randomized controlled trial.* Supportive care in cancer, 2019. **27**(1): p. S183‐.

15. Duineveld, L.A., et al., *Improving care after colon cancer treatment in The Netherlands, personalised care to enhance quality of life (I CARE study): study protocol for a randomised controlled trial.* Trials, 2015. **16**(1): p. 1-9.

16. Boele, F.W., et al., *Attitudes and preferences toward monitoring symptoms, distress, and quality of life in glioma patients and their informal caregivers.* Supportive Care in Cancer, 2016. **24**(7): p. 3011-3022.

17. Melissant, H.C., et al., *'Oncokompas', a web-based self-management application to support patient activation and optimal supportive care: a feasibility study among breast cancer survivors.* Acta Oncol, 2018. **57**(7): p. 924-934.

18. van der Hout, A., et al., *Role of eHealth application Oncokompas in supporting self-management of symptoms and health-related quality of life in cancer survivors: a randomised, controlled trial.* Lancet Oncol, 2020. **21**(1): p. 80-94.

19. Van der Hout, A., et al., *The eHealth self-management application ‘Oncokompas’ that supports cancer survivors to improve health-related quality of life and reduce symptoms: which groups benefit most?* Acta Oncologica, 2021. **60**(4): p. 403-411.

20. van den Brink, J.L., et al., *Impact on quality of life of a telemedicine system supporting head and neck cancer patients: a controlled trial during the postoperative period at home.* J Am Med Inform Assoc, 2007. **14**(2): p. 198-205.

21. van den Brink, J.L., et al., *Involving the patient: a prospective study on use, appreciation and effectiveness of an information system in head and neck cancer care.* International journal of medical informatics, 2005. **74**(10): p. 839-849.

22. van den Brink, J.L., et al., *An information system to support the care for head and neck cancer patients.* Supportive care in cancer, 2003. **11**(7): p. 452-459.

23. Keikes, L., et al., *Implementation, participation and satisfaction rates of a web-based decision support tool for patients with metastatic colorectal cancer.* Annals of Oncology, 2017. **28**: p. v201-v202.

24. Boele, F.W., et al., *Internet-based guided self-help for glioma patients with depressive symptoms: a randomized controlled trial.* Journal of neuro-oncology, 2018. **137**(1): p. 191‐203.

25. Boele, F.W., et al., *Internet-based guided self-help for glioma patients with depressive symptoms: design of a randomized controlled trial.* BMC neurology, 2014. **14**: p. 81.

26. Schook, R.M., et al., *Website visitors asking questions online to lung cancer specialists: what do they want to know?* Interactive journal of medical research, 2013. **2**(2): p. e1749.

27. Linssen, C., et al., *A web site on lung cancer: who are the users and what are they looking for?* J Thorac Oncol, 2007. **2**(9): p. 813-8.

28. Mujcic, A., et al., *Internet-based self-help smoking cessation and alcohol moderation interventions for cancer survivors: a study protocol of two RCTs.* BMC cancer, 2018. **18**(1) (no pagination).

29. van de Wiel, H.J., et al., *(Cost-)effectiveness of an internet-based physical activity support program (with and without physiotherapy counselling) on physical activity levels of breast and prostate cancer survivors: design of the PABLO trial.* BMC Cancer, 2018. **18**(1): p. 1073.

30. Kuijpers, W., et al., *An interactive portal to empower cancer survivors: a qualitative study on user expectations.* Supportive care in cancer, 2015. **23**(9): p. 2535-2542.

31. Groen, W.G., et al., *Supporting lung cancer patients with an interactive patient portal: feasibility study.* JMIR cancer, 2017. **3**(2): p. e7443.

32. Kuijpers, W., et al., *eHealth for breast cancer survivors: use, feasibility and impact of an interactive portal.* JMIR cancer, 2016. **2**(1): p. e5456.

33. Kuijpers, W., et al., *Development of MijnAVL, an interactive portal to empower breast and lung cancer survivors: an iterative, multi-stakeholder approach.* JMIR research protocols, 2015. **4**(1): p. e3796.

34. Cuypers, M., et al., *A global, incremental development method for a web-based prostate cancer treatment decision aid and usability testing in a Dutch clinical setting.* Health Informatics J, 2019. **25**(3): p. 701-714.

35. Cuypers, M., et al., *Impact of a web-based prostate cancer treatment decision aid on patient-reported decision process parameters: results from the Prostate Cancer Patient Centered Care trial.* Supportive Care in Cancer, 2018. **26**(11): p. 3739-3748.

36. Lamers, R.E., et al. *How do patients choose between active surveillance, radical prostatectomy, and radiotherapy? The effect of a preference-sensitive decision aid on treatment decision making for localized prostate cancer*. in *Urologic Oncology: Seminars and Original Investigations*. 2017. Elsevier.

37. Cuypers, M., et al., *Impact of a web-based treatment decision aid for early-stage prostate cancer on shared decision-making and health outcomes: study protocol for a randomized controlled trial.* Trials, 2015. **16**: p. 231.

38. Cuypers, M., et al., *Uptake and usage of an online prostate cancer treatment decision aid in Dutch clinical practice: A quantitative analysis from the Prostate Cancer Patient Centered Care trial.* Health Informatics J, 2019. **25**(4): p. 1498-1510.

39. van der Lee, M., *Online mindfulness-based cognitieve therapie bij kanker.* Tijdschrift voor Gedragstherapie, 2021. **2019**(2).

40. Wolvers, M., et al., *Effectiveness, Mediators, and Effect Predictors of Internet Interventions for Chronic Cancer-Related Fatigue: The Design and an Analysis Plan of a 3-Armed Randomized Controlled Trial.* JMIR Res Protoc, 2015. **4**(2): p. e77.

41. Bruggeman-Everts, F.Z., et al., *Effectiveness of two web-based interventions for chronic cancer-related fatigue compared to an active control condition: results of the “Fitter na kanker” randomized controlled trial.* Journal of medical Internet research, 2017. **19**(10): p. e336.

42. Compen, F., et al., *Face-to-face and internet-based mindfulness-based cognitive therapy compared with treatment as usual in reducing psychological distress in patients with cancer: a multicenter randomized controlled trial.* 2018.

43. Compen, F., et al., *Study protocol of a multicenter randomized controlled trial comparing the effectiveness of group and individual internet-based Mindfulness-Based Cognitive Therapy with treatment as usual in reducing psychological distress in cancer patients: the BeMind study.* BMC psychology, 2015. **3**(1): p. 1-10.

44. Wolvers, M. and M.M. Vollenbroek-Hutten. *An mHealth Intervention Strategy for Physical Activity Coaching in Cancer Survivors*. in *UMAP Workshops*. 2015.

45. van den Berg, S.W., et al., *BREATH: web-based self-management for psychological adjustment after primary breast cancer--results of a multicenter randomized controlled trial.* 2015.

46. van den Berg, S.W., et al., *Usage of a generic web-based self-management intervention for breast cancer survivors: substudy analysis of the BREATH trial.* Journal of medical internet research, 2013. **15**(8): p. e170.

47. van den Berg, S.W., et al., *Rationale of the BREAst cancer e-healTH [BREATH] multicentre randomised controlled trial: an internet-based self-management intervention to foster adjustment after curative breast cancer by decreasing distress and increasing empowerment.* BMC cancer, 2012. **12**: p. 394.

48. van Helmondt, S.J., et al., *No effect of CBT‐based online self‐help training to reduce fear of cancer recurrence: First results of the CAREST multicenter randomized controlled trial.* Psycho‐Oncology, 2020. **29**(1): p. 86-97.

49. van Helmondt, S.J., M.L. van der Lee, and J. de Vries, *Study protocol of the CAREST-trial: a randomised controlled trial on the (cost-) effectiveness of a CBT-based online self-help training for fear of cancer recurrence in women with curatively treated breast cancer.* BMC cancer, 2016. **16**(1).

50. Drijver, A.J., et al., *A Web-Based Lifestyle Intervention Aimed at Improving Cognition in Patients With Cancer Returning to Work in an Outpatient Setting: Protocol for a Randomized Controlled Trial.* JMIR Res Protoc, 2021. **10**(4): p. e22670.

51. Abrahams, H.J., et al., *A randomized controlled trial of web-based cognitive behavioral therapy for severely fatigued breast cancer survivors (CHANGE-study): study protocol.* BMC cancer, 2015. **15**: p. 765.

52. Ter Stege, J.A., et al., *The impact of an online patient decision aid for women with breast cancer considering immediate breast reconstruction: study protocol of a multicenter randomized controlled trial.* BMC medical informatics and decision making, 2019. **19**(1): p. 1-12.

53. Garvelink, M.M., et al., *Development of a decision aid about fertility preservation for women with breast cancer in the Netherlands.* Journal of Psychosomatic Obstetrics & Gynecology, 2013. **34**(4): p. 170-178.

54. Golsteijn, R.H.J., et al., *Development of a computer-tailored physical activity intervention for prostate and colorectal cancer patients and survivors: oncoActive.* BMC cancer, 2017. **17**(1).

55. Golsteijn, R.H.J., et al., *Short-term efficacy of a computer-tailored physical activity intervention for prostate and colorectal cancer patients and survivors: a randomized controlled trial.* International journal of behavioral nutrition and physical activity, 2018. **15**(1): p. 106.

56. van Bruinessen, I.R., et al., *Active patient participation in the development of an online intervention.* JMIR Res Protoc, 2014. **3**(4): p. e59.

57. van Bruinessen, I.R., et al., *An Integrated Process and Outcome Evaluation of a Web-Based Communication Tool for Patients With Malignant Lymphoma: randomized Controlled Trial.* Journal of medical Internet research, 2016. **18**(7): p. e206.

58. Tamminga, S.J., et al., *Enhancing the Return to Work of Cancer Survivors: Development and Feasibility of the Nurse-Led eHealth Intervention Cancer@Work.* JMIR Res Protoc, 2016. **5**(2): p. e118.

59. Tamminga, S.J., et al., *Cancer@Work - a nurse-led, stepped-care, e-health intervention to enhance the return to work of patients with cancer: study protocol for a randomized controlled trial.* Trials, 2016. **17**(1): p. 453.

60. Noordman, J., et al., *Evaluation and Implementation of ListeningTime: A Web-Based Preparatory Communication Tool for Elderly Patients With Cancer and Their Health Care Providers.* JMIR Cancer, 2019. **5**(1): p. e11556.

61. Noordman, J., et al., *ListeningTime; participatory development of a web-based preparatory communication tool for elderly cancer patients and their healthcare providers.* Internet interventions, 2017. **9**: p. 51-56.

62. Arts, L.P.J., et al., *Lymphoma InterVEntion (LIVE) - patient-reported outcome feedback and a web-based self-management intervention for patients with lymphoma: study protocol for a randomised controlled trial.* Trials, 2017. **18**(1): p. 199.

63. Arts, L., et al., *Participation and characterization of patients with lymphoma in a web-based selfmanagement intervention.* Psycho-oncology. Conference: 2016 world congress of psycho-oncology. Ireland. Conference start: 20161017. Conference end: 20161021, 2016. **25**: p. 108.

64. Admiraal, J.M., et al., *Web-Based Tailored Psychoeducation for Breast Cancer Patients at the Onset of the Survivorship Phase: A Multicenter Randomized Controlled Trial.* Journal of Pain and Symptom Management, 2017. **54**(4): p. 466-475.

65. Hummel, S.B., et al., *Efficacy of internet-based cognitive behavioral therapy in improving sexual functioning of breast cancer survivors: results of a randomized controlled trial.* Journal of Clinical Oncology, 2017. **35**(12): p. 1328-1340.

66. Hummel, S.B., et al., *Internet-based cognitive behavioral therapy realizes long-term improvement in the sexual functioning and body image of breast cancer survivors.* Journal of sex & marital therapy, 2018. **44**(5): p. 485-496.

67. Hummel, S.B., et al., *Internet-based cognitive behavioral therapy for sexual dysfunctions in women treated for breast cancer: design of a multicenter, randomized controlled trial.* BMC cancer, 2015. **15**(1): p. 1-12.

68. van Eenbergen, M.C., et al., *Usability of an online application for reporting the burden of side effects in cancer patients.* Support Care Cancer, 2019. **27**(9): p. 3411-3419.

69. den Bakker, C.M., et al., *Electronic Health Program to Empower Patients in Returning to Normal Activities After Colorectal Surgical Procedures: Mixed-Methods Process Evaluation Alongside a Randomized Controlled Trial.* J Med Internet Res, 2019. **21**(1): p. e10674.

70. den Bakker, C.M., et al., *Electronic health program to empower patients in returning to normal activities after general surgical and gynecological procedures: Intervention mapping as a useful method for further development.* Journal of medical Internet research, 2019. **21**(2): p. e9938.

71. Knegtmans, M.F., et al., *Home Telemonitoring Improved Pain Registration in Patients With Cancer.* Pain Pract, 2020. **20**(2): p. 122-128.

72. Atema, V., et al., *Efficacy of internet-based cognitive behavioral therapy for treatment-induced menopausal symptoms in breast cancer survivors: results of a randomized controlled trial.* Journal of Clinical Oncology, 2019. **37**(10): p. 809-822.

73. Atema, V., et al., *An Internet-based cognitive behavioral therapy for treatment-induced menopausal symptoms in breast cancer survivors: results of a pilot study.* Menopause, 2017. **24**(7): p. 762-767.

74. Atema, V., et al., *Design of a randomized controlled trial of Internet-based cognitive behavioral therapy for treatment-induced menopausal symptoms in breast cancer survivors.* BMC cancer, 2016. **16**(1): p. 1-12.

75. Verbeek, J.G.E., et al., *Cost-utility, cost-effectiveness, and budget impact of Internet-based cognitive behavioral therapy for breast cancer survivors with treatment-induced menopausal symptoms.* Breast cancer research and treatment, 2019.

76. Kaal, S.E., et al., *Online support community for adolescents and young adults (AYAs) with cancer: user statistics, evaluation, and content analysis.* Patient Prefer Adherence, 2018. **12**: p. 2615-2622.

77. van Veen, M.R., et al., *Development of a Website Providing Evidence-Based Information About Nutrition and Cancer: Fighting Fiction and Supporting Facts Online.* JMIR Res Protoc, 2015. **4**(3): p. e110.

78. Gehring, K., et al., *A pilot randomized controlled trial of exercise to improve cognitive performance in patients with stable glioma: a proof of concept.* Neuro-oncology, 2020. **22**(1): p. 103-115.

79. Gehring, K., et al., *Feasibility of a home-based exercise intervention with remote guidance for patients with stable grade II and III gliomas: a pilot randomized controlled trial.* Clin Rehabil, 2018. **32**(3): p. 352-366.

80. Visser, A., et al., *Group medical consultations (GMCs) and tablet-based online support group sessions in the follow-up of breast cancer: a multicenter randomized controlled trial.* Breast (Edinburgh, Scotland), 2018. **40**: p. 181‐188.

81. Hoek, P.D., et al., *The effect of weekly specialist palliative care teleconsultations in patients with advanced cancer -a randomized clinical trial.* BMC medicine, 2017. **15**(1): p. 119.

82. Duursma, F., et al., *Study protocol: optimization of complex palliative care at home via telemedicine. A cluster randomized controlled trial.* BMC palliative care, 2011. **10**(1): p. 1-8.

83. van den Berg, M., et al., *Development and testing of a tailored online fertility preservation decision aid for female cancer patients.* Cancer Med, 2021. **10**(5): p. 1576-1588.

84. Sungur, H., et al., *Development and Evaluation of a Digital Intervention for Fulfilling the Needs of Older Migrant Patients With Cancer: User-Centered Design Approach.* Journal of Medical Internet Research, 2020. **22**(10).

85. Yılmaz, N.G., et al., *Enhancing patient participation of older migrant cancer patients: needs, barriers, and eHealth.* Ethn Health, 2020: p. 1-24.
