# Supplementary material for: eHealth Interventions for Dutch Cancer Care: Systematic Review Using the Triple Aim Lens
Source: JMIR Cancer. 2022 Jun 14;8(2):e37093. doi: 10.2196/37093 (PMC9240931; doi:10.2196/37093)
Supplement: Multimedia Appendix 4 [file cancer_v8i2e37093_app4.docx]

# Multimedia Appendix 4. Overview of funding sources per included study

| **Study** | **Source of funding** |
| --- | --- |
| 1. Boele, F.W., et al., *Internet-based guided self-help for glioma patients with depressive symptoms: design of a randomized controlled trial.* BMC neurology, 2014. 14: p. 81. | A grant from the Dutch Cancer  Society, Alpe d’HuZes (VU 2010–4808). The first author was supported by a Niels Stensen Fellowship and a Yorkshire Cancer  Research University Academic Fellowship |
| 1. Boele, F.W., et al., *Internet-based guided self-help for glioma patients with depressive symptoms: a randomized controlled trial.* Journal of neuro-oncology, 2018. 137(1): p. 191‐203. | A grant from the Dutch Cancer  Society, Alpe d’HuZes (VU 2010–4808). The first author was supported by a Niels Stensen Fellowship and a Yorkshire Cancer  Research University Academic Fellowship |
| 1. Bruggeman-Everts, F.Z., et al., *Effectiveness of two web-based interventions for chronic cancer-related fatigue compared to an active control condition: results of the “Fitter na kanker” randomized controlled trial.* Journal of medical Internet research, 2017. 19(10): p. e336. | Alpe d'Huzes/KWF Foundation (project number 2011-5264) and extra funding by the Innovatiefonds Zorgverzekeraars and Stichting Roparun for the health care costs of eMBCT |
| 1. Wolvers, M., et al., *Effectiveness, Mediators, and Effect Predictors of Internet Interventions for Chronic Cancer-Related Fatigue: The Design and an Analysis Plan of a 3-Armed Randomized Controlled Trial.* JMIR Res Protoc, 2015. 4(2): p. e77. | Alpe d’HuZes/KWF-fonds (project number 2011-5264) |
| 1. van der Lee, M., *Online mindfulness-based cognitieve therapie bij kanker.* Tijdschrift voor Gedragstherapie, 2021. 2019(2). | No information provided |
| 1. Duursma, F., et al., *Study protocol: optimization of complex palliative care at home via telemedicine. A cluster randomized controlled trial.* BMC palliative care, 2011. 10(1): p. 1-8. | The Netherlands Organisation for  Scientific Research (NWO) |
| 1. Hoek, P.D., et al., *The effect of weekly specialist palliative care teleconsultations in patients with advanced cancer -a randomized clinical trial.* BMC medicine, 2017. 15(1): p. 119. | The Netherlands Organisation for Scientific Research (NWO) |
| 1. Willems, R.A., et al., *Long-term effectiveness and moderators of a web-based tailored intervention for cancer survivors on social and emotional functioning, depression, and fatigue: randomized controlled trial.* Journal of cancer survivorship, 2017. 11(6): p. 691‐703. | The Dutch Cancer Society (grant number NOU2011-5151) |
| 1. Willems, R.A., et al., *Working mechanisms of a web-based self-management intervention for cancer survivors: A randomised controlled trial.* Psychology & Health, 2017. 32(5): p. 605-625. | Dutch Cancer Society (grant number NOU2011-5151). |
| 1. Willems, R.A., et al., *Short-term effectiveness of a web-based tailored intervention for cancer survivors on quality of life, anxiety, depression, and fatigue: randomized controlled trial.* Psychooncology, 2017. 26(2): p. 222-230. | No information provided |
| 1. Willems, R.A., et al., *The Kanker Nazorg Wijzer (Cancer Aftercare Guide) protocol: the systematic development of a web-based computer tailored intervention providing psychosocial and lifestyle support for cancer survivors.* BMC Cancer, 2015. 15: p. 580. | Dutch Cancer Society (grant number NOU2011-5151) |
| 1. Kanera, I.M., et al., *Lifestyle-related effects of the web-based Kanker Nazorg Wijzer (Cancer Aftercare Guide) intervention for cancer survivors: a randomized controlled trial.* Journal of cancer survivorship, 2016. 10(5): p. 883‐897. | Dutch Cancer Society (grant number NOU2011-5151) |
| 1. Kanera, I.M., et al., *Long-term effects of a web-based cancer aftercare intervention on moderate physical activity and vegetable consumption among early cancer survivors: a randomized controlled trial.* International journal of behavioral nutrition and physical activity, 2017. 14(1): p. 19. | Dutch Cancer Society (grant  number NOU2011-5151) |
| 1. Kanera, I.M., et al., *Use and Appreciation of a Tailored Self-Management eHealth Intervention for Early Cancer Survivors: process Evaluation of a Randomized Controlled Trial.* Journal of medical Internet research, 2016. 18(8): p. e229. | Dutch Cancer Society (grant number NOU2011-5151) |
| 1. Duineveld, L.A., et al., *Primary care-led survivorship care for patients with colon cancer and the use of eHealth: a qualitative study on perspectives of general practitioners.* BMJ Open, 2016. 6(4): p. e010777. | KWF/Alpe D’Huzes (grant BMA 5954) |
| 1. Duman-Lubberding, S., et al., *An eHealth application in head and neck cancer survivorship care: health care professionals' perspectives.* Journal of medical Internet research, 2015. 17(10): p. e4870. | No information available |
| 1. Duman-Lubberding, S., et al., *Feasibility of an eHealth application “OncoKompas” to improve personalized survivorship cancer care.* Supportive care in cancer, 2016. 24(5): p. 2163-2171. | No information available |
| 1. Lubberding, S., et al., *Improving access to supportive cancer care through an e Health application: a qualitative needs assessment among cancer survivors.* Journal of clinical nursing, 2015. 24(9-10): p. 1367-1379. | Dutch Cancer Society (grantnumber 2010.02.023) |
| 1. van der Hout, A., et al., *Cost-utility of an eHealth application ‘Oncokompas’ that supports cancer survivors in self-management: results of a randomised controlled trial.* Journal of Cancer Survivorship, 2021. 15(1): p. 77-86. | Dutch Cancer Society (KWF Kankerbestrijding) (grant number VU 2014-7202) |
| 1. van der Hout, A., et al., *Efficacy, cost-utility and reach of an eHealth self-management application 'Oncokompas' that helps cancer survivors to obtain optimal supportive care: study protocol for a randomised controlled trial.* Trials, 2017. 18(1): p. 228. | Dutch Cancer Society/Alpe d’HuZes Fund, Pink  Ribbon, Achmea Health Care Assurance, Nutricia and Redkite (all institutional  funding) |
| 1. Schuit, A., et al., *Efficacy and cost-utility of the ehealth selfmanagement application 'oncokompas' tailored to patients with incurable cancer: study protocol of a randomized controlled trial.* Supportive care in cancer, 2019. 27(1): p. S183. | ZonMw, The Netherlands Organization for Health  Research and Development (project number: 844001105) |
| 1. Duineveld, L.A., et al., *Improving care after colon cancer treatment in The Netherlands, personalised care to enhance quality of life (I CARE study): study protocol for a randomised controlled trial.* Trials, 2015. 16(1): p. 1-9. | KWF/Stichting Alpe d’HuZes (grant BMA 5954) |
| 1. Boele, F.W., et al., *Attitudes and preferences toward monitoring symptoms, distress, and quality of life in glioma patients and their informal caregivers.* Supportive Care in Cancer, 2016. 24(7): p. 3011-3022. | No financial support |
| 1. Melissant, H.C., et al., *'Oncokompas', a web-based self-management application to support patient activation and optimal supportive care: a feasibility study among breast cancer survivors.* Acta Oncol, 2018. 57(7): p. 924-934. | Pink Ribbon (Grant 2012.PS19.C134) |
| 1. van der Hout, A., et al., *Role of eHealth application Oncokompas in supporting self-management of symptoms and health-related quality of life in cancer survivors: a randomised, controlled trial.* Lancet Oncol, 2020. 21(1): p. 80-94. | Dutch Cancer Society (KWF Kankerbestrijding) |
| 1. Van der Hout, A., et al., *The eHealth self-management application ‘Oncokompas’ that supports cancer survivors to improve health-related quality of life and reduce symptoms: which groups benefit most?* Acta Oncologica, 2021. 60(4): p. 403-411. | Dutch Cancer Society (KWF  Kankerbestrijding). Grant VU 2014-7202. |
| 1. van den Brink, J.L., et al., *Impact on quality of life of a telemedicine system supporting head and neck cancer patients: a controlled trial during the postoperative period at home.* J Am Med Inform Assoc, 2007. 14(2): p. 198-205. | OntwikkelingsBedrijf Rotterdam and  Stichting Roparun |
| 1. van den Brink, J.L., et al., *Involving the patient: a prospective study on use, appreciation and effectiveness of an information system in head and neck cancer care.* International journal of medical informatics, 2005. 74(10): p. 839-849. | OntwikkelingsBedrijf Rotterdam and  Stichting Roparun |
| 1. van den Brink, J.L., et al., *An information system to support the care for head and neck cancer patients.* Supportive care in cancer, 2003. 11(7): p. 452-459. | OntwikkelingsBedrijf Rotterdam and  Stichting Roparun |
| 1. Keikes, L., et al., *Implementation, participation and satisfaction rates of a web-based decision support tool for patients with metastatic colorectal cancer.* Annals of Oncology, 2017. 28: p. v201-v202. | Dutch Digestive Foundation |
| 1. Schook, R.M., et al., *Website visitors asking questions online to lung cancer specialists: what do they want to know?* Interactive journal of medical research, 2013. 2(2): p. e1749. | No information available |
| 1. Linssen, C., et al., *A web site on lung cancer: who are the users and what are they looking for?* J Thorac Oncol, 2007. 2(9): p. 813-8. | No information available |
| 1. Mujcic, A., et al., *Internet-based self-help smoking cessation and alcohol moderation interventions for cancer survivors: a study protocol of two RCTs.* BMC cancer, 2018. 18(1) (no pagination). | The Dutch Cancer Society (KWF Kankerbestrijding). Grant #TBOS2014–7169 |
| 1. van de Wiel, H.J., et al., *(Cost-)effectiveness of an internet-based physical activity support program (with and without physiotherapy counselling) on physical activity levels of breast and prostate cancer survivors: design of the PABLO trial.* BMC Cancer, 2018. 18(1): p. 1073. | The Dutch Cancer Society (KWF  Kankerbestrijding). |
| 1. Kuijpers, W., et al., *An interactive portal to empower cancer survivors: a qualitative study on user expectations.* Supportive care in cancer, 2015. 23(9): p. 2535-2542. | Alpe d'HuZes, a foundation which is part of the Dutch Cancer Society (KWF Kankerbestrijding). Grant number NKI 2010 – 4854 |
| 1. Groen, W.G., et al., *Supporting lung cancer patients with an interactive patient portal: feasibility study.* JMIR cancer, 2017. 3(2): p. e7443. | Alpe d'HuZes, a foundation that is part of the Dutch Cancer Society (KWF Kankerbestrijding). |
| 1. Kuijpers, W., et al., *eHealth for breast cancer survivors: use, feasibility and impact of an interactive portal.* JMIR cancer, 2016. 2(1): p. e5456. | Alpe d'HuZes, a foundation that is part of the Dutch Cancer Society (KWF Kankerbestrijding). |
| 1. Kuijpers, W., et al., *Development of MijnAVL, an interactive portal to empower breast and lung cancer survivors: an iterative, multi-stakeholder approach.* JMIR research protocols, 2015. 4(1): p. e3796. | Alpe d'HuZes, a foundation that is part of the Dutch Cancer Society (KWF Kankerbestrijding). |
| 1. Cuypers, M., et al., *A global, incremental development method for a web-based prostate cancer treatment decision aid and usability testing in a Dutch clinical setting.* Health Informatics J, 2019. 25(3): p. 701-714. | No financial support |
| 1. Cuypers, M., et al., *Impact of a web-based prostate cancer treatment decision aid on patient-reported decision process parameters: results from the Prostate Cancer Patient Centered Care trial.* Supportive Care in Cancer, 2018. 26(11): p. 3739-3748. | CZ Fund, a Dutch not for profit health insurer (Grant 2013-00070) and Delectus Foundation, a Dutch non-profit foundation aimed to initiate and stimulate research into shared decision-making. |
| 1. Lamers, R.E., et al. *How do patients choose between active surveillance, radical prostatectomy, and radiotherapy? The effect of a preference-sensitive decision aid on treatment decision making for localized prostate cancer*. in *Urologic Oncology: Seminars and Original Investigations*. 2017. Elsevier. | No information available |
| 1. Cuypers, M., et al., *Impact of a web-based treatment decision aid for early-stage prostate cancer on shared decision-making and health outcomes: study protocol for a randomized controlled trial.* Trials, 2015. 16: p. 231. | CZ Innovation Fund (grant 2013–00070), Delectus Foundation funded the decision aid development |
| 1. Cuypers, M., et al., *Uptake and usage of an online prostate cancer treatment decision aid in Dutch clinical practice: A quantitative analysis from the Prostate Cancer Patient Centered Care trial.* Health Informatics J, 2019. 25(4): p. 1498-1510. | CZ Innovation Fund (grant 2013–00070), Delectus Foundation funded the decision aid development |
| 1. Compen, F., et al., *Face-to-face and internet-based mindfulness-based cognitive therapy compared with treatment as usual in reducing psychological distress in patients with cancer: a multicenter randomized controlled trial.* 2018. | Pink Ribbon (2012.WO14.C153) |
| 1. Compen, F., et al., *Study protocol of a multicenter randomized controlled trial comparing the effectiveness of group and individual internet-based Mindfulness-Based Cognitive Therapy with treatment as usual in reducing psychological distress in cancer patients: the BeMind study.* BMC psychology, 2015. 3(1): p. 1-10. | Pink Ribbon (2012.WO14.C153) |
| 1. Wolvers, M. and M.M. Vollenbroek-Hutten. *An mHealth Intervention Strategy for Physical Activity Coaching in Cancer Survivors*. in *UMAP Workshops*. 2015. | Alpe d’HuZes/KWF-fonds, administered by the Dutch Cancer Society |
| 1. van den Berg, S.W., et al., *BREATH: web-based self-management for psychological adjustment after primary breast cancer--results of a multicenter randomized controlled trial.* 2015. | Pink Ribbon (Grant No. 2009-2013) |
| 1. van den Berg, S.W., et al., *Usage of a generic web-based self-management intervention for breast cancer survivors: substudy analysis of the BREATH trial.* Journal of medical internet research, 2013. 15(8): p. e170. | Pink Ribbon |
| 1. van den Berg, S.W., et al., *Rationale of the BREAst cancer e-healTH [BREATH] multicentre randomised controlled trial: an internet-based self-management intervention to foster adjustment after curative breast cancer by decreasing distress and increasing empowerment.* BMC cancer, 2012. 12: p. 394. | Pink Ribbon |
| 1. van Helmondt, S.J., et al., *No effect of CBT‐based online self‐help training to reduce fear of cancer recurrence: First results of the CAREST multicenter randomized controlled trial.* Psycho‐Oncology, 2020. 29(1): p. 86-97. | Pink Ribbon, Grant/Award Number: 2012.  WO43.C158 |
| 1. van Helmondt, S.J., M.L. van der Lee, and J. de Vries, *Study protocol of the CAREST-trial: a randomised controlled trial on the (cost-) effectiveness of a CBT-based online self-help training for fear of cancer recurrence in women with curatively treated breast cancer.* BMC cancer, 2016. 16(1). | Pink Ribbon |
| 1. Drijver, A.J., et al., *A Web-Based Lifestyle Intervention Aimed at Improving Cognition in Patients With Cancer Returning to Work in an Outpatient Setting: Protocol for a Randomized Controlled Trial.* JMIR Res Protoc, 2021. 10(4): p. e22670. | AM Tydeman Fonds |
| 1. Abrahams, H.J., et al., *A randomized controlled trial of web-based cognitive behavioral therapy for severely fatigued breast cancer survivors (CHANGE-study): study protocol.* BMC cancer, 2015. 15: p. 765. | Pink Ribbon (project number 2012.WO26.C139) |
| 1. Ter Stege, J.A., et al., *The impact of an online patient decision aid for women with breast cancer considering immediate breast reconstruction: study protocol of a multicenter randomized controlled trial.* BMC medical informatics and decision making, 2019. 19(1): p. 1-12. | Dutch Cancer Society  (grant number A6C/NKI 2014–7031) |
| 1. Garvelink, M.M., et al., *Development of a decision aid about fertility preservation for women with breast cancer in the Netherlands.* Journal of Psychosomatic Obstetrics & Gynecology, 2013. 34(4): p. 170-178. | DSW Health Insurance and  Pink Ribbon |
| 1. Golsteijn, R.H.J., et al., *Development of a computer-tailored physical activity intervention for prostate and colorectal cancer patients and survivors: oncoActive.* BMC cancer, 2017. 17(1). | Dutch Cancer Society (grant number NOU2012–5585). |
| 1. Golsteijn, R.H.J., et al., *Short-term efficacy of a computer-tailored physical activity intervention for prostate and colorectal cancer patients and survivors: a randomized controlled trial.* International journal of behavioral nutrition and physical activity, 2018. 15(1): p. 106. | Dutch Cancer Society (grant number NOU2012–5585). |
| 1. van Bruinessen, I.R., et al., *Active patient participation in the development of an online intervention.* JMIR Res Protoc, 2014. 3(4): p. e59. | Alpe d’HuZes program of the Dutch Cancer Society |
| 1. van Bruinessen, I.R., et al., *An Integrated Process and Outcome Evaluation of a Web-Based Communication Tool for Patients With Malignant Lymphoma: randomized Controlled Trial.* Journal of medical Internet research, 2016. 18(7): p. e206. | Alpe d’HuZes program of the Dutch Cancer Society (grant number NIVEL 2010-4747) |
| 1. Tamminga, S.J., et al., *Enhancing the Return to Work of Cancer Survivors: Development and Feasibility of the Nurse-Led eHealth Intervention Cancer@Work.* JMIR Res Protoc, 2016. 5(2): p. e118. | Stichting Alpe D’HuZes/KWF  (Dutch Cancer Society), 2011-5061 |
| 1. Tamminga, S.J., et al., *Cancer@Work - a nurse-led, stepped-care, e-health intervention to enhance the return to work of patients with cancer: study protocol for a randomized controlled trial.* Trials, 2016. 17(1): p. 453. | KWF Dutch Cancer Fund/ Alpe d’HuZes UVA 2011-5061 |
| 1. Noordman, J., et al., *Evaluation and Implementation of ListeningTime: A Web-Based Preparatory Communication Tool for Elderly Patients With Cancer and Their Health Care Providers.* JMIR Cancer, 2019. 5(1): p. e11556. | Dutch Cancer Society (KWF Kankerbestrijding: NIVEL 2014-7271) |
| 1. Noordman, J., et al., *ListeningTime; participatory development of a web-based preparatory communication tool for elderly cancer patients and their healthcare providers.* Internet interventions, 2017. 9: p. 51-56. | Dutch Cancer Society (KWF  Kankerbestrijding: NIVEL 2014-7271). |
| 1. Arts, L.P.J., et al., *Lymphoma InterVEntion (LIVE) - patient-reported outcome feedback and a web-based self-management intervention for patients with lymphoma: study protocol for a randomised controlled trial.* Trials, 2017. 18(1): p. 199. | Jonker-Driessen Foundation.  The internal funding reference number is 20011 |
| 1. Arts, L., et al., *Participation and characterization of patients with lymphoma in a web-based selfmanagement intervention.* Psycho-oncology. Conference: 2016 world congress of psycho-oncology. Ireland. Conference start: 20161017. Conference end: 20161021, 2016. 25: p. 108. | n.a. |
| 1. Admiraal, J.M., et al., *Web-Based Tailored Psychoeducation for Breast Cancer Patients at the Onset of the Survivorship Phase: A Multicenter Randomized Controlled Trial.* Journal of Pain and Symptom Management, 2017. 54(4): p. 466-475. | Pink Ribbon Foundation (grant number PR0806) |
| 1. Hummel, S.B., et al., *Efficacy of internet-based cognitive behavioral therapy in improving sexual functioning of breast cancer survivors: results of a randomized controlled trial.* Journal of Clinical Oncology, 2017. 35(12): p. 1328-1340. | The Netherlands  Cancer Institute |
| 1. Hummel, S.B., et al., *Internet-based cognitive behavioral therapy realizes long-term improvement in the sexual functioning and body image of breast cancer survivors.* Journal of sex & marital therapy, 2018. 44(5): p. 485-496. | The Netherlands  Cancer Institute |
| 1. Hummel, S.B., et al., *Internet-based cognitive behavioral therapy for sexual dysfunctions in women treated for breast cancer: design of a multicenter, randomized controlled trial.* BMC cancer, 2015. 15(1): p. 1-12. | Dutch Cancer Society (grant number NKI 2012-5388),  Pink Ribbon Foundation (grant number 2012.WO21.C138) and The  Netherlands Cancer Institute |
| 1. van Eenbergen, M.C., et al., *Usability of an online application for reporting the burden of side effects in cancer patients.* Support Care Cancer, 2019. 27(9): p. 3411-3419. | No information available |
| 1. den Bakker, C.M., et al., *Electronic Health Program to Empower Patients in Returning to Normal Activities After Colorectal Surgical Procedures: Mixed-Methods Process Evaluation Alongside a Randomized Controlled Trial.* J Med Internet Res, 2019. 21(1): p. e10674. | ZonMw (project number 837002409) |
| 1. den Bakker, C.M., et al., *Electronic health program to empower patients in returning to normal activities after general surgical and gynecological procedures: Intervention mapping as a useful method for further development.* Journal of medical Internet research, 2019. 21(2): p. e9938. | ZonMw (project number 837002409) |
| 1. Knegtmans, M.F., et al., *Home Telemonitoring Improved Pain Registration in Patients With Cancer.* Pain Pract, 2020. 20(2): p. 122-128. | No information available |
| 1. Atema, V., et al., *Efficacy of internet-based cognitive behavioral therapy for treatment-induced menopausal symptoms in breast cancer survivors: results of a randomized controlled trial.* Journal of Clinical Oncology, 2019. 37(10): p. 809-822. | Dutch Cancer Society (Grant No. NKI 2014-6788) and  The Netherlands Cancer Institute |
| 1. Atema, V., et al., *An Internet-based cognitive behavioral therapy for treatment-induced menopausal symptoms in breast cancer survivors: results of a pilot study.* Menopause, 2017. 24(7): p. 762-767. | Dutch Cancer Society (grant number NKI 2014-6788) and The Netherlands Cancer Institute |
| 1. Atema, V., et al., *Design of a randomized controlled trial of Internet-based cognitive behavioral therapy for treatment-induced menopausal symptoms in breast cancer survivors.* BMC cancer, 2016. 16(1): p. 1-12. | Dutch Cancer Society (grant number NKI 2014-6788) and The Netherlands Cancer Institute |
| 1. Verbeek, J.G.E., et al., *Cost-utility, cost-effectiveness, and budget impact of Internet-based cognitive behavioral therapy for breast cancer survivors with treatment-induced menopausal symptoms.* Breast cancer research and treatment, 2019. | Dutch Cancer Society (Grant No NKI 2014-6788) and the Netherlands Cancer Institute |
| 1. Kaal, S.E., et al., *Online support community for adolescents and young adults (AYAs) with cancer: user statistics, evaluation, and content analysis.* Patient Prefer Adherence, 2018. 12: p. 2615-2622. | Alpe d’Huzes (grant no 2011–5346) |
| 1. van Veen, M.R., et al., *Development of a Website Providing Evidence-Based Information About Nutrition and Cancer: Fighting Fiction and Supporting Facts Online.* JMIR Res Protoc, 2015. 4(3): p. e110. | KWF/Alpe d’Huzes |
| 1. Gehring, K., et al., *A pilot randomized controlled trial of exercise to improve cognitive performance in patients with stable glioma: a proof of concept.* Neuro-oncology, 2020. 22(1): p. 103-115. | Dutch Cancer Society  (UvT2010-4642) |
| 1. Gehring, K., et al., *Feasibility of a home-based exercise intervention with remote guidance for patients with stable grade II and III gliomas: a pilot randomized controlled trial.* Clin Rehabil, 2018. 32(3): p. 352-366. | Dutch Cancer Society (UvT2010-4642) |
| 1. Visser, A., et al., *Group medical consultations (GMCs) and tablet-based online support group sessions in the follow-up of breast cancer: a multicenter randomized controlled trial.* Breast (Edinburgh, Scotland), 2018. 40: p. 181‐188. | GlaxoSmithKline |
| 1. van den Berg, M., et al., *Development and testing of a tailored online fertility preservation decision aid for female cancer patients.* Cancer Med, 2021. 10(5): p. 1576-1588. | Radboud University Medical Center |
| 1. Sungur, H., et al., *Development and Evaluation of a Digital Intervention for Fulfilling the Needs of Older Migrant Patients With Cancer: User-Centered Design Approach.* Journal of Medical Internet Research, 2020. 22(10). | No information available |
| 1. Yılmaz, N.G., et al., *Enhancing patient participation of older migrant cancer patients: needs, barriers, and eHealth.* Ethn Health, 2020: p. 1-24. | KWF Kankerbestrijding |
