# Supplementary material for: eHealth Interventions for Dutch Cancer Care: Systematic Review Using the Triple Aim Lens
Source: JMIR Cancer. 2022 Jun 14;8(2):e37093. doi: 10.2196/37093 (PMC9240931; doi:10.2196/37093)
Supplement: Multimedia Appendix 5 [file cancer_v8i2e37093_app5.docx]

# Multimedia Appendix 5. List of excluded studies in the full-text screening stage

Studies are sorted by the reason for exclusion.

**No Dutch adult cancer patients or survivors**

1. Esser, P., Borchmann, P., Kuba, K., Müller, H., Görgen, H., Kreissl, S., ... & Mehnert, A. (2018). Adaptation of a web-based cognitive-behavioral therapy on fatigue for survivors of Hodgkin's lymphoma. *PPmP-Psychotherapie· Psychosomatik· Medizinische Psychologie*, *68*(08), 316.
2. Matthijs de Wit, L., van Uden-Kraan, C. F., Lissenberg-Witte, B. I., Melissant, H. C., Fleuren, M. A., Cuijpers, P., & Verdonck-de Leeuw, I. M. (2019). Adoption and implementation of a web-based self-management application “Oncokompas” in routine cancer care: a national pilot study. *Supportive Care in Cancer*, *27*(8), 2911-2920.
3. Nguyen, M. H., Smets, E. M., Bol, N., Loos, E. F., van Laarhoven, H. W., Geijsen, D., ... & Van Weert, J. C. (2019). Tailored web-based information for younger and older patients with cancer: randomized controlled trial of a preparatory educational intervention on patient outcomes. *Journal of Medical Internet Research*, *21*(10), e14407.
4. Schuit, A. S., Holtmaat, K., Hooghiemstra, N., Jansen, F., Lissenberg-Witte, B. I., Coupé, V. M., & Verdonck-de Leeuw, I. M. (2020). Efficacy and cost-utility of the eHealth self-management application'Oncokompas', helping partners of patients with incurable cancer to identify their unmet supportive care needs and to take actions to meet their needs: a study protocol of a randomized controlled trial. *Trials*, *21*(1), 1-10.
5. Spahrkäs, S. S., Looijmans, A., Sanderman, R., & Hagedoorn, M. (2020). Beating cancer-related fatigue with the untire mobile app: protocol for a waiting list randomized controlled trial. *JMIR research protocols*, *9*(2), e15969.

**Full text article not available**

1. Arts, L., Oerlemans, S., van den Berg, S., Prins, J., & van de Poll-Franse, L. (2016, October). Participation and characterization of patients with lymphoma in a web-based self-management intervention. In *Psycho-Oncology, 25*(3), p. 108.
2. Bouma, G., De Vries, E., Wymenga, M., & Walenkamp, A. (2014). Web-Based Tailored Information and Support for Patients with a Neuroendocrine Tumor. In *Neuroendocrinology*, *18*(3-4), 301.
3. Kanera, I. M., Willems, R. A., Bolman, C. A., Mesters, I., & Lechner, L. (2016). Evaluation of the use, appreciation, and adherence to a personalized module referral system of a web-based self-management intervention for early cancer survivors. *Psycho-Oncology*, *25*(S3), 20-20.
4. Van den Berg, S. W., Gielissen, M. F., Van der Graaf, W. T., Ottevanger, P. O., & Prins, J. B. (2013, November). Distress Reduction With an Unguided Self-Management Website for Women After Curative Breast Cancer Treatment: A Multicentre Randomised Controlled Trial. In *Psycho-Oncology, 22,* 112-113.
5. Van de Poll-Franse, L., Arts, L., & Oerlemans, S. (2019). Factors associated with participation in a web-based self-management intervention for lymphoma survivors: findings from an RCT embedded in the population-based PROFILES registry. In *Quality of Life Research* (Vol. 28, pp. S99-S99).

**No e-health**

1. Batenburg, A., & Das, E. (2014). Emotional approach coping and the effects of online peer-led support group participation among patients with breast cancer: a longitudinal study. *Journal of medical Internet research*, *16*(11), e3517.
2. Van Lent, L. G., Stoel, N. K., van Weert, J., van Gurp, J., de Jonge, M. J., Lolkema, M. P., ... & van der Rijt, C. C. (2019). Realizing better doctor-patient dialogue about choices in palliative care and early phase clinical trial participation: towards an online value clarification tool (OnVaCT). *BMC palliative care*, *18*(1), 1-10.
3. Van Uden-Kraan, C. F., Drossaert, C. H., Taal, E., Lebrun, C. E. I., Drossaers-Bakker, K. W., Smit, W. M., ... & van de Laar, M. A. (2008). Coping with somatic illnesses in online support groups: do the feared disadvantages actually occur?. *Computers in human behavior*, *24*(2), 309-324.

**Excluded study design**

1. Abacioglu, U. (2014). Interview with Florien Boele, MSc, and Martin Klein, MD, VU university medical center, Amsterdam, about the randomised trial on internet-based treatment of depressive symptoms in Glioma patients. *European Association of Neurooncology magazine, 4*(2), 90‐91.

**Uncompleted trial**

1. Van Helmondt, S. (2013). Investigating an online self-help training for fear of cancer recurrence in breast cancer patients. Identification No. NTR4119. Retrieved from: <http://www.who.int/trialsearch/Trial2.aspx?TrialID=NTR4119>.
2. Van Weert, J.C.M. (2016). Tailoring information to older colorectal cancer patients: Effects of using a web-based patient-directed tool. Identification No. NTR5919. Retrieved from: <http://www.who.int/trialsearch/Trial2.aspx?TrialID=NTR5919>.

**Interventions aimed at (pre-)diagnosis or prediction**

1. de Glas, N. A., van de Water, W., Engelhardt, E. G., Bastiaannet, E., de Craen, A. J., Kroep, J. R., ... & Liefers, G. J. (2014). Validity of Adjuvant! Online program in older patients with breast cancer: a population-based study. *The Lancet Oncology*, *15*(7), 722-729.
