# Supplementary material for: eHealth Interventions for Dutch Cancer Care: Systematic Review Using the Triple Aim Lens
Source: JMIR Cancer. 2022 Jun 14;8(2):e37093. doi: 10.2196/37093 (PMC9240931; doi:10.2196/37093)
Supplement: Multimedia Appendix 6 [file cancer_v8i2e37093_app6.docx]

# **Multimedia Appendix 6. Quality appraisal of the empirical evaluation studies**

| Intervention | Selection bias | Study design | Confounders | Blinding | Data collection | Withdrawals and dropouts | Global rating |
| --- | --- | --- | --- | --- | --- | --- | --- |
| Cancer Aftercare Guide (Kanker Nazorg Wijzer) |  |  |  |  |  |  |  |
| Study 1 [1] | weak | strong | strong | moderate | strong | moderate | moderate |
| Study 2 [2] | weak | strong | strong | moderate | strong | moderate | moderate |
| Study 3 [3] | weak | strong | strong | moderate | strong | moderate | moderate |
| Study 4 [4] | weak | strong | strong | moderate | strong | moderate | moderate |
| OncoCompass (OncoKompas) |  |  |  |  |  |  |  |
| Study 1 [5] | weak | strong | strong | moderate | strong | moderate | moderate |
| Study 2 [6] | weak | strong | strong | moderate | strong | moderate | moderate |
| Transmural Oncologal Support (TOS) |  |  |  |  |  |  |  |
| Study 1 [7] | moderate | weak | weak | moderate | weak | weak | weak |
| Study 2 [8] | strong | strong | strong | moderate | weak | strong | moderate |
| Everything under control (Alles onder controle) [9] | weak | strong | strong | moderate | strong | weak | weak |
| Prostate cancer decision aid (Prostaatkanker keuzehulp) [10] | moderate | strong | strong | moderate | strong | strong | strong |
| Less tired (Minder Moe) [11] | weak | strong | strong | moderate | strong | moderate | moderate |
| Less tired for anxiety/ depression complaints [12] | weak | strong | strong | moderate | strong | strong | moderate |
| BREATH [13] | moderate | strong | strong | moderate | strong | strong | strong |
| Less fear after cancer (Minder angst bij kanker) [14] | moderate | strong | strong | moderate | strong | moderate | strong |
| OncoActive [15] | weak | strong | weak | moderate | strong | strong | weak |
| PatientTIME [16] | moderate | strong | weak | moderate | strong | strong | moderate |
| ENCOURAGE [17] | moderate | strong | strong | moderate | strong | strong | strong |
| Cancer, Intimacy and Sexuality (Kanker, Intimiteit en Seksualiteit) |  |  |  |  |  |  |  |
| Study 1 [18] | weak | strong | strong | moderate | strong | strong | moderate |
| Study 2[19] | weak | strong | strong | moderate | strong | moderate | moderate |
| No name. *Home monitoring tool for adequate pain treatment* [20] | moderate | weak | strong | moderate | strong | strong | moderate |
| EvaOnline |  |  |  |  |  |  |  |
| Study 1 [21] | weak | strong | strong | moderate | strong | strong | moderate |
| Study 2 [22] | weak | strong | weak | moderate | strong | strong | moderate |
| No name. *Home-based exercise intervention* |  |  |  |  |  |  |  |
| Study 1 [23] | weak | strong | strong | moderate | weak | strong | weak |
| Study 2 [24] | weak | strong | strong | moderate | strong | strong | moderate |
| My-GMC [25] | weak | strong | strong | moderate | strong | moderate | moderate |
| No name. *Teleconsultation for patients receiving palliative home care* [26] | weak | strong | strong | moderate | strong | weak | weak |

## References

1. Willems RA, Bolman CA, Mesters I, Kanera IM, Beaulen AA, Lechner L. Short-term effectiveness of a web-based tailored intervention for cancer survivors on quality of life, anxiety, depression, and fatigue: randomized controlled trial. Psychooncology. 2017;26(2):222-30. PMID: 26988800.

2. Willems RA, Mesters I, Lechner L, Kanera IM, Bolman CAW. Long-term effectiveness and moderators of a web-based tailored intervention for cancer survivors on social and emotional functioning, depression, and fatigue: randomized controlled trial. Journal of cancer survivorship. 2017;11(6):691‐703. PMID: 28698999.

3. Kanera IM, Bolman CA, Willems RA, Mesters I, Lechner L. Lifestyle-related effects of the web-based Kanker Nazorg Wijzer (Cancer Aftercare Guide) intervention for cancer survivors: a randomized controlled trial. Journal of cancer survivorship. 2016;10(5):883‐97. PMID: 26984534.

4. Kanera IM, Willems RA, Bolman CA, Mesters I, Verboon P, Lechner L. Long-term effects of a web-based cancer aftercare intervention on moderate physical activity and vegetable consumption among early cancer survivors: a randomized controlled trial. International journal of behavioral nutrition and physical activity. 2017;14(1):19. PMID: 28187725.

5. van der Hout A, van Uden-Kraan CF, Holtmaat K, Jansen F, Lissenberg-Witte BI, Nieuwenhuijzen GAP, et al. Role of eHealth application Oncokompas in supporting self-management of symptoms and health-related quality of life in cancer survivors: a randomised, controlled trial. Lancet Oncol. 2020;21(1):80-94. PMID: 31838009.

6. van der Hout A, Jansen F, van Uden-Kraan CF, Coupé V, Holtmaat K, Nieuwenhuijzen G, et al. Cost-utility of an eHealth application ‘Oncokompas’ that supports cancer survivors in self-management: results of a randomised controlled trial. Journal of Cancer Survivorship. 2021;15(1):77-86. PMID: 32656739.

7. van den Brink JL, Moorman PW, de Boer MF, Pruyn JF, Verwoerd CD, van Bemmel JH. Involving the patient: a prospective study on use, appreciation and effectiveness of an information system in head and neck cancer care. International journal of medical informatics. 2005;74(10):839-49. PMID: 16043392.

8. van den Brink JL, Moorman PW, de Boer MF, Hop WC, Pruyn JF, Verwoerd CD, et al. Impact on quality of life of a telemedicine system supporting head and neck cancer patients: a controlled trial during the postoperative period at home. J Am Med Inform Assoc. 2007;14(2):198-205. PMID: 17213498.

9. Boele FW, Klein M, Verdonck-de Leeuw IM, Cuijpers P, Heimans JJ, Snijders TJ, et al. Internet-based guided self-help for glioma patients with depressive symptoms: a randomized controlled trial. Journal of neuro-oncology. 2018;137(1):191‐203. PMID: 29236238.

10. Cuypers M, Lamers RED, Kil PJM, van de Poll-Franse LV, de Vries M. Impact of a web-based prostate cancer treatment decision aid on patient-reported decision process parameters: results from the Prostate Cancer Patient Centered Care trial. Supportive care in cancer. 2018;26(11):3739‐48. PMID: 29752528.

11. Bruggeman-Everts FZ, Wolvers MD, Van de Schoot R, Vollenbroek-Hutten MM, Van der Lee ML. Effectiveness of two web-based interventions for chronic cancer-related fatigue compared to an active control condition: results of the “Fitter na kanker” randomized controlled trial. Journal of medical Internet research. 2017;19(10):e336. PMID: 29051138.

12. Compen F, Bisseling E, Schellekens M, Donders R, Carlson L, Lee M, et al. Face-to-face and internet-based mindfulness-based cognitive therapy compared with treatment as usual in reducing psychological distress in patients with cancer: a multicenter randomized controlled trial. 2018. PMID: 29953304.

13. van den Berg SW, Gielissen MF, Custers JA, van der Graaf WT, Ottevanger PB, Prins JB. BREATH: web-based self-management for psychological adjustment after primary breast cancer--results of a multicenter randomized controlled trial. 2015. PMID: 26169621.

14. van Helmondt SJ, van der Lee ML, van Woezik RAM, Lodder P, de Vries J. No effect of CBT‐based online self‐help training to reduce fear of cancer recurrence: First results of the CAREST multicenter randomized controlled trial. Psycho‐Oncology. 2020;29(1):86-97. PMID: 31595627.

15. Golsteijn RHJ, Bolman C, Volders E, Peels DA, de Vries H, Lechner L. Short-term efficacy of a computer-tailored physical activity intervention for prostate and colorectal cancer patients and survivors: a randomized controlled trial. International journal of behavioral nutrition and physical activity. 2018;15(1):106. PMID: 30376857.

16. van Bruinessen IR, van Weel-Baumgarten EM, Gouw H, Zijlstra JM, van Dulmen S. An Integrated Process and Outcome Evaluation of a Web-Based Communication Tool for Patients With Malignant Lymphoma: randomized Controlled Trial. Journal of medical Internet research. 2016;18(7):e206. PMID: 27473173.

17. Admiraal JM, van der Velden AWG, Geerling JI, Burgerhof JGM, Bouma G, Walenkamp AME, et al. Web-Based Tailored Psychoeducation for Breast Cancer Patients at the Onset of the Survivorship Phase: A Multicenter Randomized Controlled Trial. Journal of Pain and Symptom Management. 2017;54(4):466-75. PMID: 28711750.

18. Hummel SB, van Lankveld JJ, Oldenburg HS, Hahn DE, Kieffer JM, Gerritsma MA, et al. Efficacy of internet-based cognitive behavioral therapy in improving sexual functioning of breast cancer survivors: results of a randomized controlled trial. Journal of Clinical Oncology. 2017;35(12):1328-40. PMID: 28240966.

19. Hummel SB, Van Lankveld JJ, Oldenburg HS, Hahn DE, Kieffer JM, Gerritsma MA, et al. Internet-based cognitive behavioral therapy realizes long-term improvement in the sexual functioning and body image of breast cancer survivors. Journal of sex & marital therapy. 2018;44(5):485-96. PMID: 29297781.

20. Knegtmans MF, Wauben L, Wagemans MFM, Oldenmenger WH. Home Telemonitoring Improved Pain Registration in Patients With Cancer. Pain Pract. 2020;20(2):122-8. PMID: 31419371.

21. Atema V, Van Leeuwen M, Kieffer JM, Oldenburg HS, Van Beurden M, Gerritsma MA, et al. Efficacy of internet-based cognitive behavioral therapy for treatment-induced menopausal symptoms in breast cancer survivors: results of a randomized controlled trial. Journal of Clinical Oncology. 2019;37(10):809-22. PMID: 30763176.

22. Verbeek JGE, Atema V, Mewes JC, van Leeuwen M, Oldenburg HSA, van Beurden M, et al. Cost-utility, cost-effectiveness, and budget impact of Internet-based cognitive behavioral therapy for breast cancer survivors with treatment-induced menopausal symptoms. Breast cancer research and treatment. 2019. PMID: 31451978.

23. Gehring K, Kloek CJ, Aaronson NK, Janssen KW, Jones LW, Sitskoorn MM, et al. Feasibility of a home-based exercise intervention with remote guidance for patients with stable grade II and III gliomas: a pilot randomized controlled trial. Clin Rehabil. 2018;32(3):352-66. PMID: 28882061.

24. Gehring K, Stuiver MM, Visser E, Kloek C, van den Bent M, Hanse M, et al. A pilot randomized controlled trial of exercise to improve cognitive performance in patients with stable glioma: a proof of concept. Neuro-oncology. 2020;22(1):103-15. PMID: 31755917.

25. Visser A, Prins JB, Jansen L, Radema SA, Schlooz MS, van Dalen T, et al. Group medical consultations (GMCs) and tablet-based online support group sessions in the follow-up of breast cancer: a multicenter randomized controlled trial. Breast (Edinburgh, Scotland). 2018;40:181‐8. PMID: 29906741.

26. Hoek PD, Schers HJ, Bronkhorst EM, Vissers KCP, Hasselaar JGJ. The effect of weekly specialist palliative care teleconsultations in patients with advanced cancer -a randomized clinical trial. BMC medicine. 2017;15(1):119. PMID: 28625164.
