# Supplementary material for: eHealth Interventions for Dutch Cancer Care: Systematic Review Using the Triple Aim Lens
Source: JMIR Cancer. 2022 Jun 14;8(2):e37093. doi: 10.2196/37093 (PMC9240931; doi:10.2196/37093)
Supplement: Multimedia Appendix 7 [file cancer_v8i2e37093_app7.docx]

# Multimedia Appendix 7. Overview of the outcome measurements and found effects per empirical evaluation study

| **Intervention** | **Primary and secondary outcome measurements** | **Results** |
| --- | --- | --- |
| **RCT studies** |  |  |
| Cancer Aftercare Guide (Kanker Nazorg Wijzer) |  |  |
| Study 1 [1] | Emotional and social functioning (EORTC QLQ-C30); Anxiety and depression (HADS); Fatigue (CIS). | *e*: After six months: Emotional functioning *sig** (β=3.47, P=0.022, f²=0.013, d=0.15). Social functioning *sig** (β=3.95, P=0.011, f²=0.017, d=0.15); MT *sig.  g*: After six months: Depression *sig*** (β=-0.63, P=0.007, f²=0.019, d=0.21); MT *sig;* ITT *sig**. Fatigue *sig** (β=-4.36, P=0.020, f²=0.013, d=0.21); MT *sig;* ITT *sig*. h:* Participants in the IC who completed the six months measurement on average used 2.2 modules. Loss to follow-up in the IC was 16.2%. |
| Study 2 [2] | Emotional and social functioning (EORTC QLQ-C30); Depression (HADS); Fatigue (CIS). | *e*: After twelve months: Emotional functioning *n.s.* (β=2.65, SE=1.60, P=.096, d=0.08). Social functioning *n.s.* (β=1.31, SE=1.67, P=.435, d=0.02). g: After twelve months: Depression *n.s.* (β=−0.25, SE=0.21, P=.227, d=0.10). Fatigue *n.s.* (β=−1.01, SE=1.98, P=.611, d=0.04). *h:* Overall appreciation of the KNW is 7.48 (10 point scale)*.* |
| Study 3 [3] | Physical activity (SQUASH); Dietary behaviour (Standard Questionnaire on Food Consumption); Smoking behaviour (Measuring Instruments for Research on Smoking and Smoking Cessation). | *c*: After six months: Moderate PA *sig** (β=117.738, P=.037, d=–0.25, f²=.007); MT *n.s*. Vegetable consumption *sig** (β=9.15, P=.027, d=−0.37, f²=−.013); ITT *sig** (β=9.57, P=.023); MT *n.s.* Other PA outcomes *n.s.* (d=0.01 to 0.10; f²=.000 to .006)*;* MT *n.s.* Other dietary outcomes *n.s.* (d=-.28 to -.15, f²=.000 to .004). Smoking behaviour *n.*s. (X²=1.42, P=.233, OR 2.89).  *h*: Loss to follow-up after six months was low (11.5%) vs mean percentage of dropouts (19.7%) of web-based trials for cancer survivors. |
| Study 4 [4] | Moderate physical activity (SQUASH); Vegetable consumption (Dutch Standard Questionnaire on Food Consumption). | *c*: After twelve months: Moderate physical activity *sig*** (β=128.475, P=.010, d=.35). Vegetable consumption *n.s.* (β=5.860, P=.121). *h:* Loss to follow-up in the IC was 45.5%. |
| OncoCompass (OncoKompas) |  |  |
| Study 1 [5] | Patient's knowledge, skills and confidence for self-management (Patient Activation Measure); HRQL (EORTC QLQ-C30); Mental adjustment to cancer (mental adjustment to cancer scale); Need for supportive care (Supportive Care Needs Survey Short); Self-efficacy (General Self-Efficacy scale); Personal control (Pearlin & Schooler Mastery Scale); Perceived efficacy in patient-physician interaction (Perceived Efficacy Patient-Physician Interactions scale); Tumour-specific symptoms (EORTC tumour-specific questionnaires). | *b*: The course of symptoms in head and neck cancer survivors (pain in the mouth (P=0.010; difference at six months follow-up -8.6 [95% CI -14.2;3.1]), social eating (P=0.038; difference at six months follow-up -9.6 [95% CI -18.2; 1.0]), swallowing (P=0.045; difference at six months follow-up -6.2 [95% CI -12.5; 0.2]), coughing (P=0.017; difference at six months follow-up -7.2 [95% CI -14.2; -0.2]) and trismus (P=0.046; difference at six months follow-up -11.9 [95% CI -21.5; -2.4])), colorectal cancer survivors (weight (P=0.028; difference at six months follow-up -10.7 [95% CI -18.1; -3.3])) and high-grade non-Hodgkin lymphoma survivors (emotional impacts (P=0.049; difference at six months follow-up -3.2 [95% CI -12.4; 6.0])) *sig*.* The course of symptoms in BC survivors *n.s.* (P=0.31 to 0.98; difference at six months follow up ranged from -3.3 to 5.2). *e:* HRQOL *sig** (P=0.048; difference at six months follow-up 2.3 [95% CI 0.0; 4.5]). *g*: Course of mental adjustment to cancer *n.s.* (P=0.77 to 1.00; difference at six months follow up ranged from -1.1 to 0.5).  *h:* Course of supportive care needs *n.s.* (P=0.18 to 0.50; difference at six months follow up ranged from -5.2 to -1.0). Patient-physician interaction over time *n.s.* (P=0.22; difference at six months follow-up 0.4 [95% CI -0.1; 0.9]). Self-efficacy *n.s.* (P= 0.31, difference at six months follow up 0.5 [95% CI -0.4; 1.4]). Personal control *n.s.* (P= 0.68, difference at six months follow up 0.9 [95% CI 0.0; 1.7]). Patient activation *n.s*. (difference at six months follow-up 1.7 [95% CI−0.8; 4.1; P=0.41]). In the IC, 78% activated their account, and 52% used the intervention as intended. |
| Study 2 [6] | Direct medical and direct non-medical costs (iMTA iMCQ); Indirect non-medical costs (iPCQ); Productivity losses (absenteeism and presenteeism in the past three months); HRQoL (EQ-5D); Utility score (Dutch index tariff); Intervention costs (total cost divided by users per year). | *h:* The loss to follow up in the IC was 36%. *l*: OncoCompass is likely to be equally effective on utilities and not more expensive than usual care. |
| Everything under control (Alles onder controle) [7] | Depressive symptoms (CES-D); Fatigue (CIS); HRQL (SF-36); Disease-specific HRQoL (EORTC Brain Cancer Module); Cognitive functioning (MOS cognitive functioning scale); Use of supportive care (TIC-P); Patient satisfaction (study-specific questionnaire). | *e*: After six and twelve weeks (GI vs GWL group and Total glioma group vs Non-CNS cancer group): Physical health *n.s.  g*: After six weeks: Depression (GI vs GWL group and Total glioma group vs Non-CNS cancer group) *n.s.* Fatigue (GI vs GWL group) *sig** (P= 0.054, d=0.306). After twelve weeks: depression *n.s.* Fatigue *n.s.* Other measures (GI vs GWL group) *n.s. h*: Most patients said they had benefitted from participating (73% glioma; 67% non-CNS), and the program was useful (92% in both groups) and informative (86% glioma; 92% non-CNS). The participation rate was 40%. The adherence of the IC was 85% for the introduction and 77, 52, 40, 37 and 35% for modules one through five, respectively. |
| Prostate cancer decision aid (Prostaatkanker keuzehulp) [8] | Decisional conflict (DCS); Patient’s perceived role during decision-making (Problem-Solving Decision-Making Scale); Perceived preparedness to make treatment decision (Preparation for Decision-making Scale); Pca knowledge (perceived knowledge level per treatment and objective test based on the Pca Decision Quality Instrument); Satisfaction with timing and format of the information (SCIP-B). | *h:* Satisfaction with information *sig** (β=-0.25, P=0.04). Involvement *n.s.*  Decisional conflict *n.s.* Knowledge scores *n.s.* Subjective knowledge *sig*** (β=0.43, P=0.01). Objective knowledge *n.s.* |
| Less tired (Minder Moe) [9] | Fatigue severity (CIS-FS); Positive and negative affect (Positive and Negative Affect Schedule); Psychic complaints (HADS). | *g*: Fatigue severity (AAF vs psycho-education X²=28.28, P<0.001; eMBCT vs psycho-education X²=10.89, P=0.004) *sig*.* Psychic complaints (AAF vs psycho-education and eMBCT vs psycho-education) *n.s.* Positive and negative affect (AAF vs psycho-education and eMBCT vs psycho-education) *n.s.  h:* The proportion of participants who dropped out before completing six weeks of the protocol was 18% in the AAF condition, 38% in the eMBCT and 6% in the psycho-education condition. |
| Less tired for anxiety/ depression complaints [10] | Psychological distress (HADS); Psychiatric diagnosis (Structured Clinical Interview for DSM-IV-TR Axis I Disorders); Fear of cancer recurrence (Fear of Cancer Recurrence Inventory); Rumination (Rumination and Reflection Questionnaire); HRQoL (SF using Dutch norms); Mindfulness skills (Five Facet Mindfulness Questionnaire-SF); Positive mental health (Mental Health Continuum-SF). | *b:* Psychiatric diagnosis *n.s.* (MBCT: 32% v 16%; x2 [1, n=126]=4.73;P=.030; and eMBCT: 29% v 16%; x2 [1, n=138]=3.15; P=.076). *c*: TAU vs MBCT and TAU vs EMBCT: Mindfulness skills *sig** (Cohen’s d, .47 and .82, respectively).  *e:* Mental HRQoL *sig** (Cohen’s d, .59 and.67, respectively)*.* Positive mental health *sig** (Cohen’s d, .12 and .44, respectively). Physical HRQoL *n.s.* (Cohen’s d, .35 and .24, respectively). *g*: Psychological distress *sig*** (Cohen’s d, .45 and .71, respectively; MBCT vs TAU (36% v 14%; x2 [1, n=134]=8.44; P=.004) and eMBCT vs TAU (37% v 14%; x2 [1, n=145]=9.95; P=.002)*.* Fear of cancer recurrence *sig** (Cohen’s d, .27 and .53, respectively)*.* Rumination *sig* (*Cohen’s d, .42 and .51, respectively). *h:* 90.9% started MBCT, and 92.2% completed four or more sessions. 91.1% started eMBCT, and 71 completed four or more sessions. The dropout rate was higher in EMBCT than in the MBCT. |
| BREATH [11] | Psychological distress (SCL-90); Psychological empowerment (CEQ); Negative adjustment: general  and cancer-specific distress (DT), fatigue (CIS-fatigue), helplessness (Illness Cognition Questionnaire) and fear of cancer recurrence (CWS and CAS) and positive adjustment: self-efficacy (SES), remoralization (RS), personal control (Mastery Scale), quality of life (QLQ-C30 and QLQ-BR23), fulfilment (PAQ), re-evaluation (PAQ), new ways of living (PAQ) and valuing life (PAQ). | *g*: At T1: Distress *sig** (-7.79 [95% CI, -14.31; -1.27], P=.02; d=0.33). Five out of seven negative adjustment variables (general and cancer-specific distress, fatigue, and two fear of cancer recurrence outcomes) and three out of ten positive adjustment variables (self-efficacy, remoralization, new ways of living) *sig** (d= 0.37 to 0.55)*.* Clinically significant improvement *sig** (More CAU+BREATH participants (39 of 70 [56%], [95% CI, 44.1; 66.8]) than CAU-alone participants (32 of 80 [40%], [95% CI, 30.0; 51.0]) showed clinically significant improvement (P=.03). At T2 and T3*:* Distress *n.s.* One negative adjustment variable (Fear of cancer recurrence) *sig**(F[3, 120= 4.563; P=.005)*.* One positive adjustment outcome (Acceptance) *sig*** (significantly improved in both groups (ICQ; F[3, 120]=8.531; P= .000)). All other outcomes *n.s. h:* At T1: Empowerment *n.s*. (-1.71, [95% CI 5.20; -1.79], P=.34). The frequency of logins ranged from 0 to 45. Total duration ranged from 0 to 2.324 minutes. |
| Less fear after cancer (Minder angst bij kanker) [12] | Fear of cancer recurrence (FCRI) | *g*: Fear of cancer recurrence *n.s.* (χ²= .23, P=.63). *h:* The dropout rate in the IC was 30%*.* |
| OncoActive [13] | PA (SQUASH and an ActiGraph GT3X-BT accelerometer); Fatigue (CIS); Distress (HADS); HRQoL (EORTC QLQ-C30); Global health status (2 items on a 7-point scale); Physical functioning (four items on a 5-point scale). | *c*: At three months: PA *sig** (β=133.55, P=.04)*;* ITT *sig.*  *e*: At three months: Physical functioning *sig*** (β=2.61, P=.003)*;* ITT *sig.* HRQoL *n.s.* (β=0.18, P=.82). At six months follow-up: physical functioning *sig** (β=1.86, P=.04); ITT *n.s.* HRQoL *n.s.* (β=1.09, P=.37). *g*: At three months follow-up: Fatigue *sig** (β=− 3.57, P=.02). At six months follow-up: Fatigue *sig*** (β=− 4.16, P=009). Depression *sig*** (β=− 0.64, P=.005), ITT sig. Anxiety *n.s.* (β=0.14, P=.54). *h:* Dropout rates were 4.4% at three months follow-up and 7.3% at six months follow-up. |
| PatientTIME [14] | Patients’ confidence in their ability to communicate with a healthcare provider (PEPPI); Usability (System Usability Scale). | *h*: System usability scale: 73 points (100-point scale), considered “good”. At T1 and T2*:* PEPPI score *n.s.* (P=.78). The participation rate was 90%. |
| ENCOURAGE [15] | Increased optimism and control over the future (CEO); Distress (DT/PL); QoL (EORTC QLQ-C30 and QLQ-BR23). | *e*: QoL at T2 *n.s.  g*: At T1: Increased acceptance *n.s.* Other primary outcomes *n.s.* At T2: All outcomes *n.s.  h*: Usefulness score of the program 3.75 (5-point scale) At T1: Being better-informed *sig** (group difference -0.31 [95% CI -0.57; -0.05], P=0.020). At T2*: n.s.* 61% of the patients logged in more than once. |
| Cancer, Intimacy and Sexuality (Kanker, Intimiteit en Seksualiteit) |  |  |
| Study 1 [16] | Sexual functioning (FSFI and SAQ); Sexual distress (FSDS-R); Relationship intimacy (PAIR); Body image (QLQ-BR23); Marital functioning [MMQ]); Menopausal symptoms (FACT-ES); Psychological distress (HADS); HRQoL (SF-36). | *e*: At T1: Sexual desire *sig*** (respectively ES=.48, P=<0.001). Sexual pleasure *sig*** (respectively ES=.32, P=0.10). Discomfort during sex *sig*** (ES=.49, P=0.003). Orgasmic function *n.s.* Sexual satisfaction *n.s.* Sex frequency *n.s.* Relationship intimacy *n.s.* Marital functioning *n.s.* Health-related quality of life *n.s.* At T2: Overall sexual functioning *sig** (ES=.43, P=0.009). Sexual desire *sig** (*ES=.72, P=<0.001). Sexual arousal *sig*** (ES=.50, P=0.002). Vaginal lubrication *sig** (ES=.46, P=0.004). Sexual pleasure (ES=.62, P=<0.001). Discomfort during sex *sig*** (ES=.66, P=0.001). Orgasmic function *n.s.* Sexual satisfaction *n.s.* Sex frequency *n.s.* Relationship intimacy *n.s.* Marital functioning *n.s.* Health-related quality of life *n.s. g*: At T1: Menopausal symptoms *sig*** (ES=.45, P=0.005). Body image *sig*** (ES=.45, P=.005). Psychological distress *n.s*. (ES=.01, P=.946). At T2: Menopausal symptoms *n.s.* (ES=.26, P=.103). Body image *sig*** (ES=.45, P=.005). Psychological distress *n.s*. (ES=.04, P=.690).  *h:* The CBT was completed by 61.9% of women. |
| Study 2 [17] | Sexual functioning (FSFI and SAQ); Sexual distress (FSDS-R); Relationship intimacy (PAIR); Body image (QLQ-BR23); Marital functioning [MMQ]); Menopausal symptoms (FACT-ES); Psychological distress (HADS); HRQoL (SF-36). | *a*: *Only time-effect was taken into account as T3 and T4 assessments were completed only by the IC*. At T3 and T4: general health *positive* *effect maintained.  e:* At T3 and T4: Sexual functioning (P=.238), sexual desire (P= .246), vaginal lubrication (P=.069), sexual satisfaction (P=.455), discomfort during sex (P=.264), sexual distress (P=.977), marital sexual satisfaction *positive* *effect maintained.* Sex frequency (P=.028), intellectual intimacy (P=.003) and sexual pleasure (P=.044) *decreased over time*. Marital satisfaction and other health-related quality of life domains *n.s. time effect. g:* At T3 and T4: Menopausal symptoms (P=.001) and body image (P=.003) *positive effect was maintained,* quadratic effect *n.s. time effect.* Distress *n.s. time effect. h:* The CBT was completed by 61.9% of women. |
| EvaOnline |  |  |
| Study 1 [18] | Hot flushes and night sweats problem or HF/NS (HFRS); Overall levels of menopausal symptoms (FACT-ES); Sexual functioning (SAQ); Sleep quality (GSQS); Hot flush frequency (HFRS); Psychological distress (HADS); HRQoL (SF-36 Health Survey). | *e*: Sexual functioning in both IC groups (guided and self-managed) *n.s.* (ES=. HRQOL *n.s.  g*: At T1: Both IC groups (guided and self-managed) perceived impact of HF/NS *sig*** (ES= .63 and .56, respectively; both P<.001). Guided group overall levels of menopausal symptoms *sig*** (ES=.33, P=.003)*.* Both IC groups’ sleep quality *sig*** (ES=.57 and .41; both P<.001). Guided hot flush frequency *sig.* Guided group night sweats frequency *sig*** (ES=.64, P=<.001). Psychological distress *n.s.* (ES=.345 and .231 respectively, P=.10 and .13). *h:* Minimum compliance rate was 90.6% for the guided and 78.8% for the self-managed ICs. |
| Study 2 [19] | HRQoL (SF-36); Utility score (EQ-5D); QALYs (utility scores multiplied with years of life); Menopause-specific measures (FACT-ES); Perceived impact of HF/NS (HFRS); Measurement and valuation of costs (online platform and therapist support); Valuations of the resources used (cost information provided by two potential Dutch providers of iCBT and invoices obtained during RCT); Direct healthcare costs (iMCQ); Valuation of visits to healthcare providers (Dutch costing manual for economic evaluations). | l: The guided and self-managed iCBT are cost-effective. Self-managed iCBT is the most cost-effective strategy. |
| No name. *Home-based exercise intervention* |  |  |
| Study 1 [20] | Self-reported physical activity (IPAQ); Physical outcomes (exercise tests to calculate Vo2 peak). | *c*: Self-reported physical activity at six months *sig** (average increase was 126% in the IC vs 23% in the UC). BMI at six months *n.s.* Mean absolute Vo2 peak at six months *n.s.* Aerobic fitness at six months *sig* (+158.9mL/min; [95% CI 44.8; 362.5], and 2.0mL/kg/min [95% CI: −0.4; 4.4, d=0.24]). *h:* 16 patients (84%) evaluated the physical exercise program as good or excellent, and four as moderately/sufficiently satisfactory. Mean adherence was 79%. |
| Study 2 [21] | Attention (Stroop Color-Word Test, Letter Digit Substitution Test, WAIS-R Digit span and Test of Everyday Attention); Memory (Visual Verbal Learning Test and WMS-III Verbal Paired Associates); Executive function (Concept Shifting Test, GIT Letter Fluency, GIT Category Fluency and Test of Everyday Attention); Subjective cognitive functioning (MOS Cognitive Functioning Scale and Cognitive Failure Questionnaire); Subjective fatigue and sleep (Multidimensional Fatigue Inventory and Pittsburgh Sleep Quality Index); Mood (Profile of Mood States); QoL (HRQL questionnaire and MOS Short-Form 36). | *e*: For attention, four measures (attentional inhibition (β=+0.52 [95% CI=0.05;0.99]), attention span (β=+0.57 [95% CI=-0.01; 1.14]), auditory selective attention (β=+0.51 [95% CI=0.03; 0.98]), working memory (β=+0.51 [95% CI=0.03; 0.98]) *sig.* Information processing speed *sig*. (β=+0.47 [95% CI=−0.16; 1.10). Sustained selective attention *n.s.* For memory, immediate verbal recall *sig* (β=+0.29 [95% CI=−0.56; 1.13]). Two measures of executive function (auditory working memory (β=+0.25 [95% CI=−0.23; 0.73]), alternating attention (β=+0.35 [95% CI=−0.30; 0.99]) *sig.* One of two measures of cognitive functioning *sig* (β=+0.20 [95% CI=−0.45; 0.86]). Mood *sig* (β=+0.50 [95% CI=0.04; 0.96]). Mental health-related quality of life *sig* (β=+0.63 [95% CI=−0.16; 1.41]). Brain cancer-specific health-related quality of life scales *n.s. h:* Loss to follow up in the IC was 8.7%. *g*: Two scales of fatigue *sig* (physical fatigue (β= +0.52 [95% CI=−0.23; 1.27]) and reduced activity (β=+0.63 [95% CI=−0.17; 1.43])*.* Sleep *sig* (β=+0.34 [95% CI=−0.23; 0.91]). |
| My-GMC [22] | Psychological distress (SCL-90); Empowerment (Empowerment Questionnaire for BC patients); Fear of recurrence (CWS); QoL (EORTC-QLQC30 and EORTC-BR23); Medication adherence (MARS); Patient and professional satisfaction (5-point scale); | *c*: Medication adherence at T2 *sig (*adj. mean difference=4.298 [95% CI .0; 1.1], F=.042).  *e*: Quality of life at all time points *n.s. g:* Distress at all time points *n.s. C*ancer worry at all time points *n.s. h*: Satisfaction with the online app was rated 2.8 (5-point scale). Professional satisfaction with the video GMCs was 2.7 (5-point scale). Empowerment at all time points *n.s.* The participation rate was 35%. |
| No name. *Teleconsultation for patients receiving palliative home care* [23] | Patient-experienced symptom burden (ESAS and HADS). | *b:* Symptom burden *n.s. g*: Anxiety *n.s. (*adjusted anxiety scores were significantly higher for IC; β=1.40 [95% CI 0.14; 2.66]; P=0.03). Depression *n.s.* (β=0.30 [95% CI −1.39; 1.99, P=0.73). All three subscales for continuity of care *n.s.* (β=0.15 to 0.29, P=0.13 to 0.39).  *h*: Study outcome measures regarding GP contacts and complex interventions *n.s.* Mean number of unmet needs *n.s.* (β= –0.01 [95% CI −0.07; 0.04, P=0.67). The attrition rates were 61% in the IC and 53% in the CG.  *m:* Mean number of hospital admissions *n.s. (*IC group= 0.47, UC group= 0.38, P=0.60). |
| **PCT studies** |  |  |
| Transmural Oncologal Support (TOS) |  |  |
| Study 1 [24] | Evaluation of the system (1. analysing the log files and evaluating all messages and e-mail alerts; 2. using a paper-based questionnaire addressing the use and appreciation of the system). | *h:* The average score of all patients for the monitoring function was 8.0 (10-point scale). The average score rated by seven GPs of the electronic health information support system was 5.6 (10-point scale). The participation rate was 66%. All patients used the system. |
| Study 2 [25] | QoL dimensions (model of coping with cancer developed by Van den Borne and Pruyn and three subscales were developed additionally). | *e*: After the intervention: Five of the 22 QoL subscales (“state anxiety,” (-4.53; std. error 1.82; P=0.01) “fear related to specific head and neck problems,” (-2.59, std. error 1.82, P=0.02) “physical self-efficacy,” (2.39; std. error 1.07, P=0.03) “perceived abilities in swallowing and food intake,” (2.63; std. error 1.29; P=0.04) and “general physical complaints.” (-1.27; std. error 0.52, P= 0.02) *sig.* At three months, one subscale (physical self-efficacy) *sig** (3.08, std. error 1.29, P=0.02)*.* Other subscales *n.s.  h:* The participation rate in the IC was 66%, and 35 out of 39 patients completed all questionnaires. |
| **Before and after design studies** |  |  |
| No name. *Home monitoring tool for adequate pain treatment* [26] | Medical data and pain-related data (medical record data). | *g:* Total number of “pain registrations” in the medical records *sig** (37%; P=0.034). |

## References

1. Willems, R.A., et al., *Short-term effectiveness of a web-based tailored intervention for cancer survivors on quality of life, anxiety, depression, and fatigue: randomized controlled trial.* Psychooncology, 2017. **26**(2): p. 222-230.

2. Willems, R.A., et al., *Long-term effectiveness and moderators of a web-based tailored intervention for cancer survivors on social and emotional functioning, depression, and fatigue: randomized controlled trial.* Journal of cancer survivorship, 2017. **11**(6): p. 691‐703.

3. Kanera, I.M., et al., *Lifestyle-related effects of the web-based Kanker Nazorg Wijzer (Cancer Aftercare Guide) intervention for cancer survivors: a randomized controlled trial.* Journal of cancer survivorship, 2016. **10**(5): p. 883‐897.

4. Kanera, I.M., et al., *Long-term effects of a web-based cancer aftercare intervention on moderate physical activity and vegetable consumption among early cancer survivors: a randomized controlled trial.* International journal of behavioral nutrition and physical activity, 2017. **14**(1): p. 19.

5. van der Hout, A., et al., *Role of eHealth application Oncokompas in supporting self-management of symptoms and health-related quality of life in cancer survivors: a randomised, controlled trial.* Lancet Oncol, 2020. **21**(1): p. 80-94.

6. van der Hout, A., et al., *Cost-utility of an eHealth application ‘Oncokompas’ that supports cancer survivors in self-management: results of a randomised controlled trial.* Journal of Cancer Survivorship, 2021. **15**(1): p. 77-86.

7. Boele, F.W., et al., *Internet-based guided self-help for glioma patients with depressive symptoms: a randomized controlled trial.* Journal of neuro-oncology, 2018. **137**(1): p. 191‐203.

8. Cuypers, M., et al., *Impact of a web-based prostate cancer treatment decision aid on patient-reported decision process parameters: results from the Prostate Cancer Patient Centered Care trial.* Supportive care in cancer, 2018. **26**(11): p. 3739‐3748.

9. Bruggeman-Everts, F.Z., et al., *Effectiveness of two web-based interventions for chronic cancer-related fatigue compared to an active control condition: results of the “Fitter na kanker” randomized controlled trial.* Journal of medical Internet research, 2017. **19**(10): p. e336.

10. Compen, F., et al., *Face-to-face and internet-based mindfulness-based cognitive therapy compared with treatment as usual in reducing psychological distress in patients with cancer: a multicenter randomized controlled trial.* 2018.

11. van den Berg, S.W., et al., *BREATH: web-based self-management for psychological adjustment after primary breast cancer--results of a multicenter randomized controlled trial.* 2015.

12. van Helmondt, S.J., et al., *No effect of CBT‐based online self‐help training to reduce fear of cancer recurrence: First results of the CAREST multicenter randomized controlled trial.* Psycho‐Oncology, 2020. **29**(1): p. 86-97.

13. Golsteijn, R.H.J., et al., *Short-term efficacy of a computer-tailored physical activity intervention for prostate and colorectal cancer patients and survivors: a randomized controlled trial.* International journal of behavioral nutrition and physical activity, 2018. **15**(1): p. 106.

14. van Bruinessen, I.R., et al., *An Integrated Process and Outcome Evaluation of a Web-Based Communication Tool for Patients With Malignant Lymphoma: randomized Controlled Trial.* Journal of medical Internet research, 2016. **18**(7): p. e206.

15. Admiraal, J.M., et al., *Web-Based Tailored Psychoeducation for Breast Cancer Patients at the Onset of the Survivorship Phase: A Multicenter Randomized Controlled Trial.* Journal of Pain and Symptom Management, 2017. **54**(4): p. 466-475.

16. Hummel, S.B., et al., *Efficacy of internet-based cognitive behavioral therapy in improving sexual functioning of breast cancer survivors: results of a randomized controlled trial.* Journal of Clinical Oncology, 2017. **35**(12): p. 1328-1340.

17. Hummel, S.B., et al., *Internet-based cognitive behavioral therapy realizes long-term improvement in the sexual functioning and body image of breast cancer survivors.* Journal of sex & marital therapy, 2018. **44**(5): p. 485-496.

18. Atema, V., et al., *Efficacy of internet-based cognitive behavioral therapy for treatment-induced menopausal symptoms in breast cancer survivors: results of a randomized controlled trial.* Journal of Clinical Oncology, 2019. **37**(10): p. 809-822.

19. Verbeek, J.G.E., et al., *Cost-utility, cost-effectiveness, and budget impact of Internet-based cognitive behavioral therapy for breast cancer survivors with treatment-induced menopausal symptoms.* Breast cancer research and treatment, 2019.

20. Gehring, K., et al., *Feasibility of a home-based exercise intervention with remote guidance for patients with stable grade II and III gliomas: a pilot randomized controlled trial.* Clin Rehabil, 2018. **32**(3): p. 352-366.

21. Gehring, K., et al., *A pilot randomized controlled trial of exercise to improve cognitive performance in patients with stable glioma: a proof of concept.* Neuro-oncology, 2020. **22**(1): p. 103-115.

22. Visser, A., et al., *Group medical consultations (GMCs) and tablet-based online support group sessions in the follow-up of breast cancer: a multicenter randomized controlled trial.* Breast (Edinburgh, Scotland), 2018. **40**: p. 181‐188.

23. Hoek, P.D., et al., *The effect of weekly specialist palliative care teleconsultations in patients with advanced cancer -a randomized clinical trial.* BMC medicine, 2017. **15**(1): p. 119.

24. van den Brink, J.L., et al., *Involving the patient: a prospective study on use, appreciation and effectiveness of an information system in head and neck cancer care.* International journal of medical informatics, 2005. **74**(10): p. 839-849.

25. van den Brink, J.L., et al., *Impact on quality of life of a telemedicine system supporting head and neck cancer patients: a controlled trial during the postoperative period at home.* J Am Med Inform Assoc, 2007. **14**(2): p. 198-205.

26. Knegtmans, M.F., et al., *Home Telemonitoring Improved Pain Registration in Patients With Cancer.* Pain Pract, 2020. **20**(2): p. 122-128.
